# Bimekizumab safety and efficacy in patients with psoriatic arthritis: 3-year results from two phase 3 studies

**DOI:** 10.1093/rheumatology/keag118

**Published:** 2026-03-16

**Authors:** Laure Gossec, Laura C Coates, Robert B M Landewé, Philip J Mease, Joseph F Merola, Christopher T Ritchlin, Yoshiya Tanaka, Akihiko Asahina, Fabian Proft, Nadine Goldammer, Myriam Manente, Barbara Ink, Rajan Bajracharya, Jason Coarse, Iain B McInnes

**Affiliations:** INSERM, Institut Pierre Louis d‘Epidémiologie et de Santé Publique, Sorbonne Université, Paris, France; APHP, Rheumatology Department, Pitié-Salpêtrière Hospital, Paris, France; Nuffield Department of Orthopaedics, Rheumatology and Musculoskeletal Diseases, University of Oxford and Oxford Biomedical Research Centre, Oxford University Hospitals NHS Trust, Oxford, UK; Amsterdam Rheumatology & Clinical Immunology Center, Amsterdam, and Zuyderland MC, Heerlen, The Netherlands; Department of Rheumatology, Providence-Swedish Medical Center and University of Washington, Seattle, WA, USA; Department of Dermatology and Department of Medicine, Division of Rheumatology, UT Southwestern Medical Center, Dallas, TX, USA; Allergy, Immunology & Rheumatology Division, University of Rochester Medical School, Rochester, NY, USA; Department of Molecular Targeted Therapeutics, University of Occupational and Environmental Health, Kitakyushu, Japan; Department of Dermatology, The Jikei University School of Medicine, Tokyo, Japan; Department of Gastroenterology, Infectiology and Rheumatology (including Nutrition Medicine), Charité – Universitätsmedizin Berlin, corporate member of Freie Universität Berlin and Humboldt-Universität zu Berlin, Berlin, Germany; UCB, Monheim am Rhein, Germany; UCB, Braine-l’Alleud, Belgium; UCB, Slough, UK; UCB, Slough, UK; UCB, Morrisville, NC, USA; College of Medical Veterinary and Life Sciences, University of Glasgow, Glasgow, UK

**Keywords:** psoriatic arthritis, bimekizumab, efficacy, safety, open-label extension, IL-17 inhibition

## Abstract

**Objectives:**

Bimekizumab, a monoclonal IgG1 antibody that selectively inhibits IL-17F in addition to IL-17A, has demonstrated tolerability and clinical efficacy in patients with PsA. Here, we report an additional year of safety and efficacy of bimekizumab treatment to 3 years.

**Methods:**

BE OPTIMAL [NCT03895203; biologic DMARD (bDMARD)-naïve] and BE COMPLETE [NCT03896581; prior inadequate response/intolerance to TNF inhibitors (TNFi-IR)] assessed s.c. bimekizumab 160 mg every 4 weeks in patients with PsA. Study completers could enrol in the BE VITAL open-label extension (NCT04009499). Outcomes were reported as observed, or using modified non-responder or multiple imputation, to 3 years.

**Results:**

Overall, 546/299 (76.7/74.8%) bDMARD-naïve/TNFi-IR patients randomized to bimekizumab or placebo at baseline (Bimekizumab Total group) completed year 3. Treatment-emergent adverse event rates [exposure-adjusted incidence rate/100 patient-years (95% CI)] for bimekizumab-treated patients through 3 years were 164.2 (152.7–176.3) in bDMARD-naïve and 88.6 (79.1–98.9) in TNFi-IR patients, consistent with those at year 1 with no new safety signals identified. Response rates for efficacy outcomes were sustained up to 3 years; at year 1 and year 3, respectively, 56.1/50.4% and 53.2/55.2% of bDMARD-naïve/TNFi-IR patients achieved ACR50, 61.8/58.2% and 59.5/59.1% achieved swollen joint count resolution, and 64.7/66.2% and 61.9/67.5% had 100% improvement from baseline in Psoriasis Area and Severity Index. Responses for other efficacy measures were similarly sustained and consistent in bDMARD-naïve and TNFi-IR patients.

**Conclusion:**

Bimekizumab demonstrated sustained high levels of efficacy and tolerability to 3 years, supporting its suitability for long-term treatment in bDMARD-naïve and TNFi-IR patients with PsA.

**Trial registration:**

BE OPTIMAL: NCT03895203; BE COMPLETE: NCT03896581; BE VITAL: NCT04009499.

## Graphical Abstract

**Graphical Abstract:**
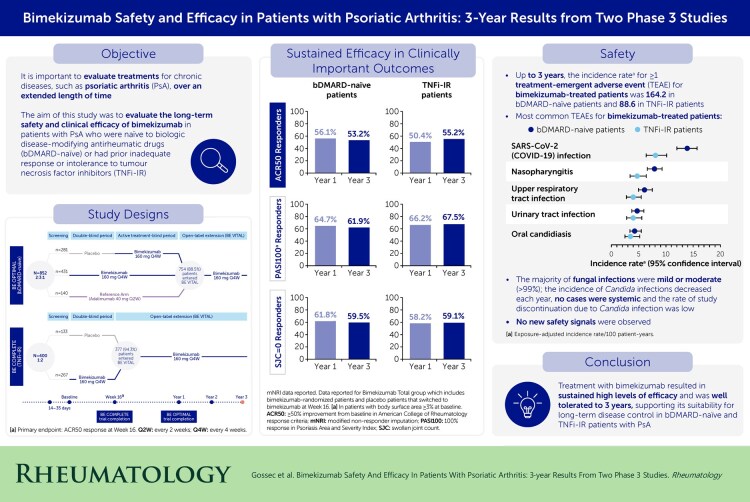

Rheumatology key messages

- Bimekizumab is well suited for long-term treatment of patients with active psoriatic arthritis (PsA).
- We found there were no new safety signals in patients with PsA after an additional year of bimekizumab treatment.
- High levels of efficacy were sustained to 3 years in both biologic-naïve and biologic-experienced patients.

## Introduction

PsA is a chronic, inflammatory disease that affects multiple domains, including joints, skin, and nails [1, 2]. PsA can have a marked detrimental impact on patients’ functional ability and quality of life over an extended period [3]. As randomized controlled trials (RCTs) typically span relatively short time frames, the Group for Research and Assessment of Psoriasis and Psoriatic Arthritis (GRAPPA) and EULAR have highlighted the importance of collecting and reporting longer-term follow-up data for chronic diseases [2, 4].

Together with an acceptable safety profile, sustained control of inflammation is essential for the treatment of patients with PsA to prevent or minimize cumulative joint damage and functional impairment throughout their lifetime [2]. IL-17 plays a pivotal role in the chronic inflammation and associated tissue modulation and comorbidities seen in patients with PsA [5–7]. IL-17A and IL-17F are drivers of joint and skin inflammation that share ∼50% homology and also overlap in their pro-inflammatory function [1, 8]. While IL-17A is more potent than IL-17F, IL-17F is produced in higher quantities, which is thought to be associated with chronic inflammation in the joints [1, 9]. Dual neutralization of both of these ILs may therefore be important in maintaining long-term treatment response [8, 10].

Bimekizumab is a humanized, monoclonal IgG1 antibody that selectively inhibits IL-17F in addition to IL-17A [11]. Previous analyses have demonstrated consistent levels of clinical response with bimekizumab treatment for up to 2 years in patients with PsA who were biologic DMARD (bDMARD)-naïve and those had experienced inadequate response or intolerance to TNF inhibitors (TNFi-IR) in the phase 3 BE OPTIMAL and BE COMPLETE studies, and their open-label extension (OLE) BE VITAL [12–14].

The objective of the present study was to assess the safety and clinical efficacy of bimekizumab in patients with active PsA who were bDMARD-naïve or TNFi-IR, from the BE VITAL OLE study to 3 years.

## Methods

### Study designs

Complete methodologies were reported to week 16 and 24 in the BE OPTIMAL and BE COMPLETE primary publications [15, 16], respectively; further details can be found in the subsequent 1- and 2-year publications [12–14]. All studies assessed s.c. bimekizumab 160 mg every 4 weeks (Q4W) in patients with active PsA who met the CASPAR criteria.

In brief, BE OPTIMAL (NCT03895203; ClinicalTrials.gov) was a 52-week, phase 3, randomized, double‑blind, placebo‑controlled study of bDMARD-naïve patients with active PsA. A reference arm [adalimumab 40 mg every 2 weeks (Q2W)] was included to provide a standard-of-care reference for bimekizumab treatment. BE COMPLETE (NCT03896581; ClinicalTrials.gov) was a 16-week, phase 3, randomized, double-blind, placebo-controlled study of patients with active PsA and prior TNFi-IR. BE VITAL (NCT04009499; ClinicalTrials.gov) is an OLE study of BE OPTIMAL and BE COMPLETE. All patients who entered BE VITAL received bimekizumab 160 mg Q4W, regardless of their prior randomization. The study designs can be found in Supplementary Fig. S1.

### Patients

Inclusion and exclusion criteria for BE OPTIMAL and BE COMPLETE, and eligibility criteria for enrolment in BE VITAL, have been reported previously [13, 15, 16]. Patients were eligible for enrolment into the BE VITAL OLE study if they completed week 52 of BE OPTIMAL or week 16 of BE COMPLETE, met the eligibility criteria of the BE VITAL OLE, and provided separate informed consent. Decisions to withdraw patients for any reason, including but not limited to persistent active disease, adverse events (AEs), or patient decision, were taken at the discretion of the investigator.

### Outcomes

The primary objective of BE VITAL was to assess the long-term safety and tolerability of bimekizumab in adult patients with PsA over a period of 140 weeks following enrolment into the study. The secondary objective was to assess the long-term efficacy of bimekizumab.

Here, safety and efficacy outcomes are reported up to 3 years from the baseline of BE OPTIMAL and BE COMPLETE. Results for patients originally enrolled in BE OPTIMAL or BE COMPLETE are referred to throughout as ‘BE OPTIMAL’ or ‘BE COMPLETE’, respectively. Safety outcomes are presented for patients who received at least one dose of bimekizumab. The BE OPTIMAL and BE COMPLETE Bimekizumab Total groups included bimekizumab-randomized patients and patients who switched from placebo to bimekizumab at week 16 (placebo/bimekizumab); equivalent to all patients in BE COMPLETE. The BE OPTIMAL All Patients group included bimekizumab-randomized patients, placebo/bimekizumab patients, and patients who switched from the reference arm (adalimumab) to bimekizumab at week 52. Only events after the switch from placebo or reference arm to bimekizumab are included. Efficacy outcomes are presented for bimekizumab-randomized patients and placebo/bimekizumab patients separately to year 1, and then for the Bimekizumab Total groups from year 1 to year 3.

Safety outcomes, reported to 3 years (week 156 in BE OPTIMAL and BE COMPLETE), include treatment-emergent AEs (TEAEs), serious TEAEs, and study discontinuations due to TEAEs. Other safety results reported include drug-related TEAEs, severe TEAEs, study and permanent treatment discontinuations due to TEAEs, deaths, and other safety topics of interest [adjudicated major adverse cardiovascular events (MACE), neutropenia, serious infections, fungal infections, hypersensitivity, injection site reactions, adjudicated suicidal ideation and behaviour, malignancies, adjudicated IBD, uveitis, and liver enzyme elevations]. MACE, suicidal ideation and behaviour, hepatic events, and IBD events were adjudicated by external committees.

Clinical efficacy outcomes, reported through 3 years (week 160 in BE OPTIMAL and week 156 in BE COMPLETE), include improvements from baseline of ≥20%, ≥50%, and ≥70% in the ACR response criteria (ACR20/50/70) [17], resolution of swollen joint count (SJC), improvements from baseline in Psoriasis Area and Severity Index (PASI) of ≥75%, ≥90% and 100% [PASI75/90/100; in patients with psoriasis affecting ≥3% body surface area (BSA) at baseline] [18], and resolution of nail psoriasis [modified Nail Psoriasis Severity Index (mNAPSI)=0 in patients with nail psoriasis (mNAPSI > 0) at baseline].

Additional clinical outcomes include achievement of minimal and very low disease activity criteria (MDA, VLDA) [19] reported to 3 years (week 160 in BE OPTIMAL and week 156 in BE COMPLETE), as well as the proportion of patients achieving improvements from baseline in ACR50+PASI100 (in patients with psoriasis affecting ≥3% BSA at baseline). Other reported efficacy end points: PASI ≤1 or BSA ≤3% responders, Disease Activity Index for Psoriatic Arthritis (DAPSA) [20] remission (REM) and low disease activity (LDA), Psoriatic Disease Activity Score (PASDAS) [21] REM and LDA, enthesitis [Leeds Enthesitis Index (LEI)=0] [22], dactylitis [Leeds Dactylitis Index (LDI)=0] [23], and high-sensitivity CRP (hs-CRP) change from baseline (CfB) and normalization.

Patient-reported outcomes (PROs) reported through 3 years (week 148 or 160 in BE OPTIMAL and week 156 in BE COMPLETE) include the Psoriatic Arthritis Impact of Disease 12-item (PsAID-12) questionnaire total score clinically meaningful improvement (decrease from baseline of ≥3 in patients with PsAID-12 ≥ 3 at baseline) and CfB [24], HAQ-DI minimal clinically important difference (MCID; decrease from a baseline of ≥0.35 in patients with a baseline score of ≥0.35) and CfB [25, 26], the BASDAI total score CfB [27], the Pain VAS CfB and achievement of substantial improvement in pain (≥50% improvement in Pain VAS) [28, 29], the Functional Assessment of Chronic Illness Therapy-Fatigue (FACIT-Fatigue) CfB and MCID (increase from baseline of ≥4 in patients with a FACIT-Fatigue subscale of ≤48 at baseline) [30].

### Statistical analysis

Statistical powering and sample size determination were reported in the previous publications [13, 15, 16]. Descriptive statistics are used to provide an overview of the safety and efficacy results. Baseline values for efficacy variables were determined from baseline values of the respective initial studies (week 0 in BE OPTIMAL and BE COMPLETE), as per the EULAR guidance for reporting clinical trial extension data [4].

The Safety Set consisted of all randomized patients who received at least one dose of bimekizumab. Safety variables were analysed for all patients in the Safety Set. AEs were coded according to the Medical Dictionary for Regulatory Activities (MedDRA version 19.0). Safety outcomes are reported as exposure-adjusted incidence rates (EAIRs) per 100 patient-years (PY) of exposure, with associated 95% CIs.

The Randomized Set consisted of all enrolled patients randomized in the respective initial study. Efficacy variables were analysed for all patients in the Randomized Set and reported here for the bimekizumab-randomized patients and placebo/bimekizumab patients to year 1, and the Bimekizumab Total groups from year 1 to year 3.

Modified non-responder imputation (mNRI) and non-responder imputation (NRI) were used to impute missing data for binary outcomes. mNRI considered all visits following discontinuation due to AEs or lack of efficacy as non-response; all other missing data were imputed with multiple imputation (MI) and the response derived from the imputed values [31]. MI was used to impute missing data for continuous outcomes. Data were imputed using baseline patient numbers from the initial studies. Any patients who did not enter the BE VITAL OLE were imputed as non-responders, as per EULAR guidance for reporting clinical trial extension data [4]. Observed case (OC) data are also reported. All analyses were done with SAS, version 9.3 or higher.

## Results

### Patient disposition and baseline characteristics

Following completion of BE OPTIMAL at week 52, 88.9% (633/712) of patients in the Bimekizumab Total group (bimekizumab-randomized patients and placebo/bimekizumab patients) entered BE VITAL; 76.7% (546/712) completed to week 160. Patients were encouraged to stay in the study even if they discontinued treatment. Ten (1.4%) patients in BE OPTIMAL completed study visits to week 160 not on randomized treatment. Of 140 patients in the reference arm, 121 (86.4%) entered BE VITAL and 77.1% (108/140) completed to week 160. Following completion of BE COMPLETE at week 16, 94.3% (377/400) of patients in the Bimekizumab Total group entered BE VITAL; 74.8% (299/400) completed to week 156. Four (1.0%) patients completed study visits to week 156 not on randomized treatment.

The full Safety Set for BE OPTIMAL included 823 patients in the All Patients group, and 702 patients in the Bimekizumab Total group. The Safety Set for BE COMPLETE included 388 patients in the Bimekizumab Total group. The Randomized Set included 712 patients in the BE OPTIMAL Bimekizumab Total group, 400 patients in the BE COMPLETE Bimekizumab Total group and 140 patients in the BE OPTIMAL reference arm.

To week 160 in BE OPTIMAL and week 156 in BE COMPLETE, 79/712 and 43/400 patients in the respective Bimekizumab Total groups discontinued treatment due to AEs or lack of efficacy; these patients were considered as non-responders in mNRI analyses.

Patient disposition by randomization group at baseline is provided in Supplementary Fig. S2. Patient demographics and baseline characteristics have been reported previously and were representative of patients with moderate-to-severe PsA with long-standing disease [12, 14–16]. In general, TNFi-IR patients had a longer time since diagnosis and greater disease severity compared with bDMARD-naïve patients (Supplementary Table S1).

### Safety

Patients treated with bimekizumab in the All Patients Safety Set in BE OPTIMAL and Bimekizumab Total Safety Set in BE COMPLETE up to 3 years had a TEAE incidence rate (EAIR/100 PY) of 164.2 (95% CI: 152.7–176.3) and 88.6 (79.1–98.9), respectively (Table 1). The incidence rates of serious TEAEs were 6.5 (5.4–7.8) and 5.7 (4.2−7.4) in BE OPTIMAL and BE COMPLETE, respectively. The incidence rates of study discontinuations due to TEAEs were similar between trials. The three most common TEAEs to 3 years in both trials were SARS-CoV-2 (COVID-19) infection, nasopharyngitis, and upper respiratory tract infection. Four deaths occurred in patients treated with bimekizumab; three occurred before 2 years, as previously described [12, 14]. One death occurred due to cardiac arrest between year 2 and year 3 in a 66 year-old patient randomized to bimekizumab at baseline of BE OPTIMAL. The patient had a history of hypertension, heart failure, chronic obstructive pulmonary disease, and aortic aneurysm, and concomitant medications including MTX, folic acid, amlodipine, beclometasone dipropionate, formoterol fumarate, montelukast sodium, diclofenac sodium, acetylsalicylic acid, rosuvastatin, methylprednisolone, and ketoconazole. All deaths occurring during the study periods were reported by the study investigators as unrelated to treatment. TEAEs by year are provided in Supplementary Table S2. The incidence rates of any TEAEs, study discontinuations due to TEAEs, and drug-related TEAEs decreased each year in both studies.

**Table 1:** Safety overview to 3 years.

	**BE OPTIMAL** (bDMARD-naïve)		**BE COMPLETE** (TNFi-IR)
*n* ^a^ (%) [EAIR/100 PY; 95% CI]	BKZ 160 mg Q4W Total^b^ *n* = 702 (1794.3 PY)	BKZ 160 mg Q4W All patients^c^ *N* = 823 (2022.1 PY)	BKZ 160 mg Q4W Total^b^ *N* = 388 (985.3 PY)
Any TEAE	650 (92.6) [168.1; 155.5–181.6]	755 (91.7) [164.2; 152.7–176.3]	318 (82.0) [88.6; 79.1–98.9]
Serious TEAEs^d^	114 (16.2) [6.9; 5.7–8.3]	122 (14.8) [6.5; 5.4–7.8]	52 (13.4) [5.7; 4.2–7.4]
Study discontinuations due to TEAEs	60 (8.5) [3.4; 2.6–4.4]	65 (7.9) [3.3; 2.5–4.2]	27 (7.0) [2.8;1.8–4.0]
Permanent treatment discontinuations due to TEAEs	65 (9.3) [3.7; 2.8–4.7]	70 (8.5) [3.5; 2.7–4.4]	29 (7.5) [3.0; 2.0–4.3]
Drug-related TEAEs	324 (46.2) [27.4; 24.5–30.5]	365 (44.3) [26.9; 24.2–29.8]	130 (33.5) [17.1; 14.3–20.3]
Severe TEAEs	61 (8.7) [3.5; 2.7–4.6]	66 (8.0) [3.4; 2.6–4.3]	35 (9.0) [3.7; 2.6–5.2]
Death	2 (0.3)^e^^,^^f^ [0.1; 0.0–0.4]	3 (0.4)^e^^,^^f^ [0.2; 0.0–0.4]	1 (0.3)^e^^,^^g^ [0.1; 0.0–0.6]
**Most frequently reported TEAEs (five most common TEAEs in any BKZ-treated group at the year 3 data cut)**			
SARS-CoV-2 (COVID-19) infection^h^	205 (29.2) [13.3; 11.5–15.2]	240 (29.2) [13.9; 12.2–15.7]	72 (18.6) [8.1; 6.4–10.2]
Nasopharyngitis	125 (17.8) [8.0; 6.7–9.6]	139 (16.9) [7.8; 6.6–9.3]	44 (11.3) [4.8; 3.5–6.5]
Upper respiratory tract infection	102 (14.5) [6.2; 5.1–7.6]	113 (13.7) [6.1; 5.0–7.4]	38 (9.8) [4.1; 2.9–5.6]
Urinary tract infection	83 (11.8) [5.0; 4.0–6.2]	90 (10.9) [4.8; 3.8–5.9]	37 (9.5) [4.0; 2.8–5.6]
Oral candidiasis	73 (10.4) [4.3; 3.4–5.5]	82 (10.0) [4.3; 3.4–5.4]	34 (8.8) [3.6; 2.5–5.1]
**Safety topics of interest**			
Serious infections	25 (3.6) [1.4; 0.9–2.1]	28 (3.4) [1.4; 0.9–2.0]	13 (3.4) [1.3; 0.7–2.3]
Opportunistic infections^i^	18 (2.6) [1.0; 0.6–1.6]	19 (2.3) [1.0; 0.6–1.5]	3 (0.8) [0.3; 0.1–0.9]
Active tuberculosis	0	0	0
Fungal infections	144 (20.5) [9.2; 7.8–10.9]	163 (19.8) [9.2; 7.9–10.8]	52 (13.4) [5.8; 4.3–7.6]
* Candida* infection	97 (13.8) [5.9; 4.8–7.2]	106 (12.9) [5.7; 4.7–6.9]	37 (9.5) [4.0; 2.8–5.5]
Oral candidiasis	73 (10.4) [4.3; 3.4–5.5]	82 (10.0) [4.3; 3.4–5.4]	34 (8.8) [3.6; 2.5–5.1]
Fungal infections NEC	61 (8.7) [3.6; 2.8–4.6]	71 (8.6) [3.7; 2.9–4.7]	18 (4.6) [1.9; 1.1–3.0]
Any neutropenia	22 (3.1)^j^ [1.3; 0.8–1.9]	22 (2.7)^j^ [1.1; 0.7–1.7]	13 (3.4)^k^ [1.4; 0.7–2.3]
Serious hypersensitivity reactions	0	0	1 (0.3)^l^ [0.1; 0.0–0.6]
Any administration/injection site reaction^m^	24 (3.4) [1.4; 0.9–2.0]	28 (3.4) [1.4; 0.9–2.1]	8 (2.1) [0.8; 0.4–1.6]
Elevated liver enzymes, *n*/*N* (%)^n^	71/702 (10.1) [4.3; 3.3–5.4]	80/823 (9.7) [4.2; 3.4–5.3]	38/388 (9.8) [4.1; 2.9–5.7]
>3× ULN ALT/AST	34/701 (4.9) [2.0; 1.4–2.7]	39/822 (4.7) [2.0; 1.4–2.7]	17/388 (4.4) [1.8; 1.0–2.8]
>5× ULN ALT/AST	9/701 (1.3) [0.5; 0.2–1.0]	10/822 (1.2) [0.5; 0.2–0.9]	8/388 (2.1) [0.8; 0.4–1.6]
Adjudicated MACE	7 (1.0) [0.4; 0.2–0.8]	9 (1.1) [0.5; 0.2–0.9]	2 (0.5) [0.2; 0.0–0.7]
Malignancies,^o^ excluding non-melanoma skin cancer	9 (1.3) [0.5; 0.2–1.0]	9 (1.1) [0.5; 0.2–0.9]	10 (2.6) [1.0; 0.5–1.9]
Adjudicated suicidal ideation and behaviour^p^	2 (0.3) [0.1; 0.0–0.4]	2 (0.2) [0.1; 0.0–0.4]	0
Adjudicated IBD^q^	5 (0.7)^r^ [0.3; 0.1–0.7]	7 (0.9)^r^ [0.4; 0.1–0.7]	1 (0.3)^s^ [0.1; 0.0–0.6]
Uveitis^t^	4 (0.6) [0.2; 0.1–0.6]	4 (0.5) [0.2; 0.1–0.5]	0

Safety set. Data reported to 3 years (week 156).

a ‘*n*’ denotes the number of patients reporting at least one of the respective TEAEs;

b Bimekizumab Total group includes bimekizumab-randomized patients and placebo-randomized patients who switched to bimekizumab at week 16; includes events after switch only;

c All Patients group includes all patients who had received at least one dose of bimekizumab, including patients who switched to bimekizumab from the reference arm (adalimumab) at week 52; includes events after switch only;

d Serious TEAEs met one or more of the following criteria: death, life‑threatening event, significant or persistent disability/incapacity, congenital anomaly/birth defect (including in a foetus), important medical event, or initial inpatient hospitalization or prolonged hospitalization;

e Considered not related to the study drug;

f Earlier deaths as previously described [13]; one additional death between year 2 and year 3 due to cardiac arrest in a 66-year old patient randomized to bimekizumab at baseline with a history of cardiovascular disease and multiple concomitant medications;

g Sudden death in year 1 as previously described [13];

h Specific terms for SARS-CoV-2 (COVID-19) infections were not available in the MedDRA v19.0; confirmed or suspected cases were identified using the preferred terms ‘corona virus infection’ and ‘coronavirus test positive’;

i No cases of histoplasmosis, blastomycosis, or coccidioidomycosis were reported;

j Includes 20 patients with neutropenia and 2 patients with neutrophil count decreased;

k Includes 8 patients with neutropenia and 6 patients with neutrophil count decreased;

l One case of dermatitis classed as serious due to the patient requiring hospitalization;

m Identified using the high-level terms ‘administration site reactions NEC’ and ‘injection site reactions’;

n Elevated liver enzymes included the following preferred terms reported as adverse events: increased/abnormal levels of ALT, AST, blood bilirubin, gamma-glutamyltransferase, hepatic enzyme, liver function test, total bile acids, or transaminases;

o Malignancies were reported in an additional 5 patients in BE OPTIMAL and 1 patient in BE COMPLETE when including non-melanoma skin cancer;

p No cases of completed suicide reported;

q Cases deemed definite or probable IBD by the investigator;

r One patient with prior history of IBD;

s Patient did not have prior history of IBD;

t Uveitis TEAEs identified using the preferred terms ‘autoimmune uveitis’, ‘iridocyclitis’, ‘iritis’, and ‘uveitis’.

ALT: alanine aminotransferase; AST: aspartate aminotransferase; bDMARD: biologic DMARD; BKZ: bimekizumab; EAIR: exposure-adjusted incidence rate; MACE: major adverse cardiac event; NEC: not elsewhere classified; PBO: placebo; PY: patient-years; Q4W: every 4 weeks; TEAE: treatment-emergent adverse event; TNFi-IR: prior inadequate response or intolerance to TNF inhibitors; ULN: upper limit of normal.

The incidence rates of fungal infections reported by the investigators to 3 years were 9.2 (7.9–10.8) in BE OPTIMAL and 5.8 (4.3−7.6) in BE COMPLETE (Table 1). The rates of *Candida* infections were 5.7 (4.7−6.9) and 4.0 (2.8–5.5) in BE OPTIMAL and BE COMPLETE, respectively. Most of these cases were oral candidiasis. There was one case of serious oropharyngeal candidiasis in BE OPTIMAL, no cases of serious candidiasis in BE COMPLETE, and no cases of systemic *Candida* infections in either study. Study discontinuation due to *Candida* infection was low, and there were no instances of patients discontinuing bimekizumab treatment due to fungal infection recurrence. Further breakdown of fungal infections can be found in Supplementary Table S3. Incidence rates of fungal infections, particularly *Candida* infections, decreased each year in BE OPTIMAL and decreased after year 1 and remained low in BE COMPLETE (Supplementary Table S3; Supplementary Fig. S3).

The incidence rate of malignancies to 3 years was low and similar between studies (Table 1); a further breakdown of malignancies can be found in Supplementary Table S4. In addition, the incidence rates to 3 years were low and similar between studies for events adjudicated as MACE, neutropenia, and administration/injection site reactions (Table 1; Supplementary Table S5). There were no cases of serious hypersensitivity in BE OPTIMAL, and one case of dermatitis classified as serious due to the patient requiring hospitalization in BE COMPLETE (which was resolved). There were low incidence rates of events adjudicated as suicidal ideation and behaviour, IBD, and uveitis in both studies (Table 1; Supplementary Table S6). Incidence rates of elevated liver enzymes were also low (Table 1) and decreased each year in both studies (Supplementary Table S2).

### Efficacy

**Figure 1:**
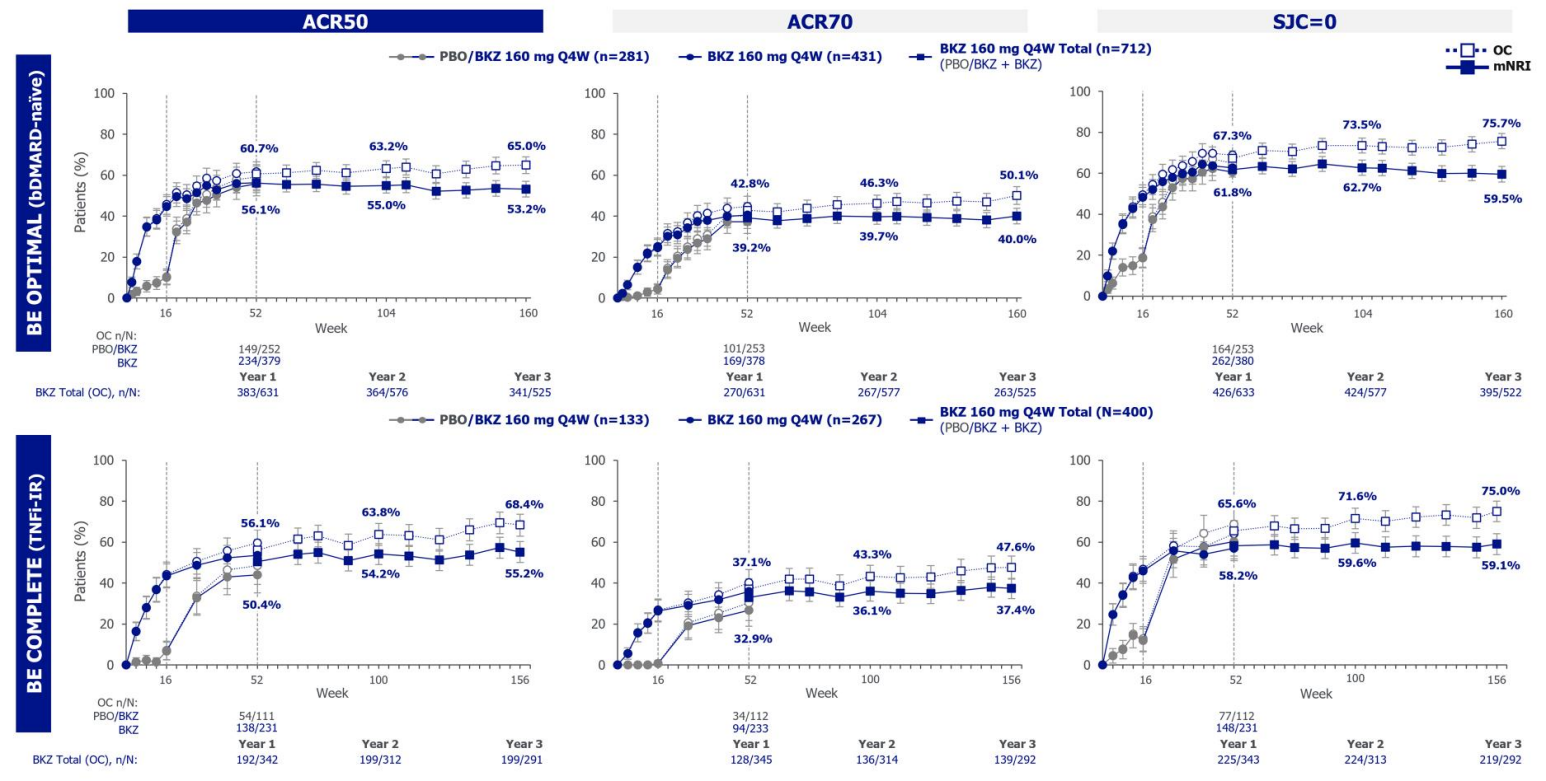
Joint outcomes to 3 years (mNRI, OC). Randomized set. Bimekizumab Total group includes bimekizumab-randomized patients and placebo-randomized patients who switched to bimekizumab at week 16. Data reported to 3 years (week 160 in BE OPTIMAL and week 156 in BE COMPLETE). mNRI considered all visits following discontinuation due to AEs or lack of efficacy as non-response; all other missing data were imputed with multiple imputation and the response derived from the imputed values. Error bars represent 95% CIs. ACR50/70: ≥50/70% improvement from baseline in ACR response criteria; AE: adverse event; bDMARD: biologic DMARD; BKZ: bimekizumab; mNRI: modified non-responder imputation; OC: observed case; PBO: placebo; Q4W: every 4 weeks; SJC: swollen joint count; TNFi-IR: prior inadequate response or intolerance to TNF inhibitors

**Figure 2:**
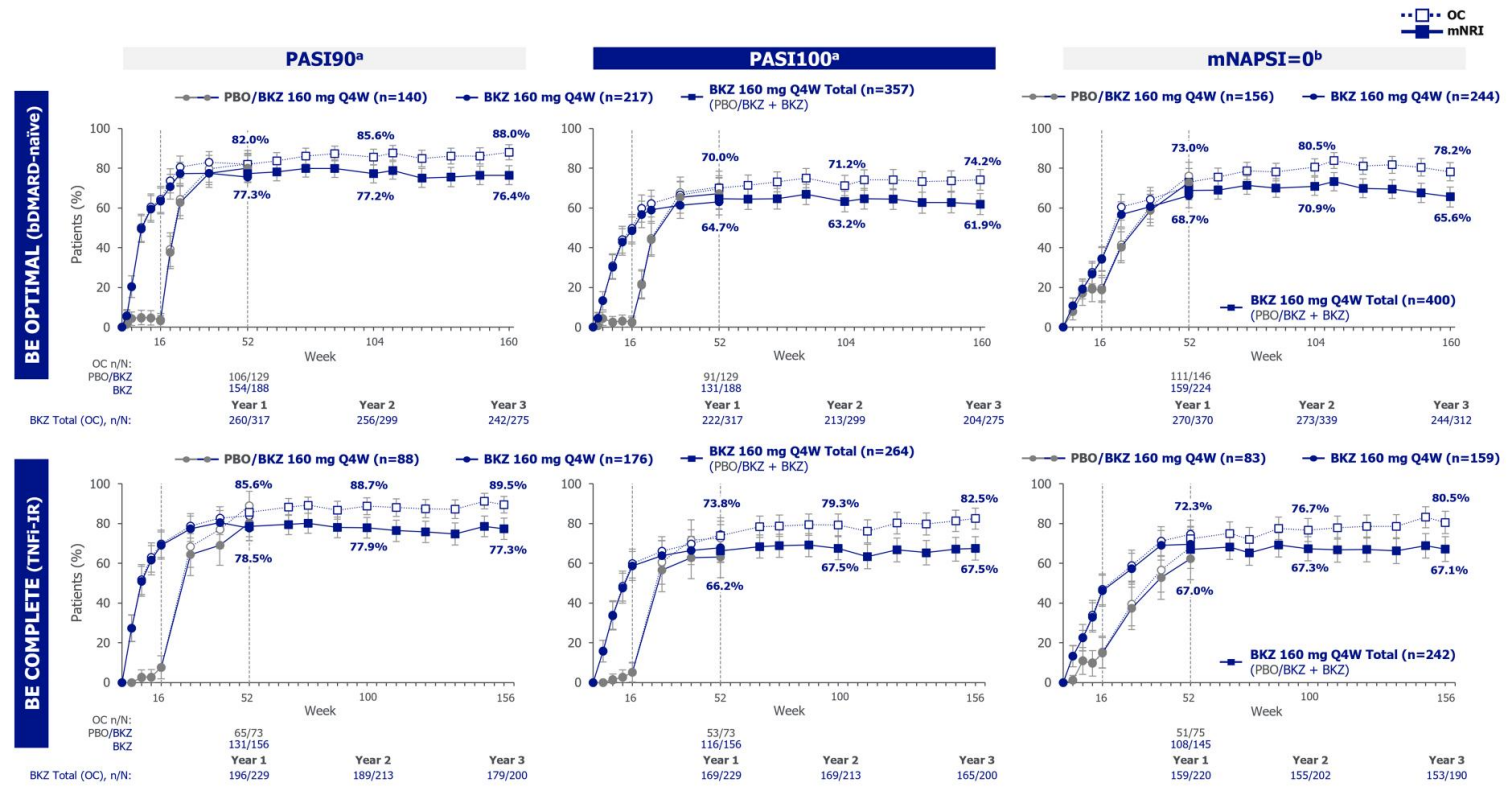
Skin and nail outcomes to 3 years (mNRI, OC). Randomized set. Bimekizumab Total group includes bimekizumab-randomized patients and placebo-randomized patients who switched to bimekizumab at week 16. Data reported to 3 years (week 160 in BE OPTIMAL and week 156 in BE COMPLETE). mNRI considered all visits following discontinuation due to AEs or lack of efficacy as non-response; all other missing data were imputed with multiple imputation and the response derived from the imputed values. ^a^In patients with ≥3% BSA at baseline; ^b^In patients with nail psoriasis (mNAPSI >0) at baseline. Error bars represent 95% CIs. AE: adverse event; bDMARD: biologic DMARD; BKZ: bimekizumab; BSA: body surface area; mNAPSI: modified Nail Psoriasis Severity Index; mNRI: modified non-responder imputation; OC: observed case; PASI90/100: ≥90/100% improvement from baseline in Psoriasis Area and Severity Index; PBO: placebo; Q4W: every 4 weeks; TNFi-IR: prior inadequate response or intolerance to TNF inhibitors

**Figure 3:**
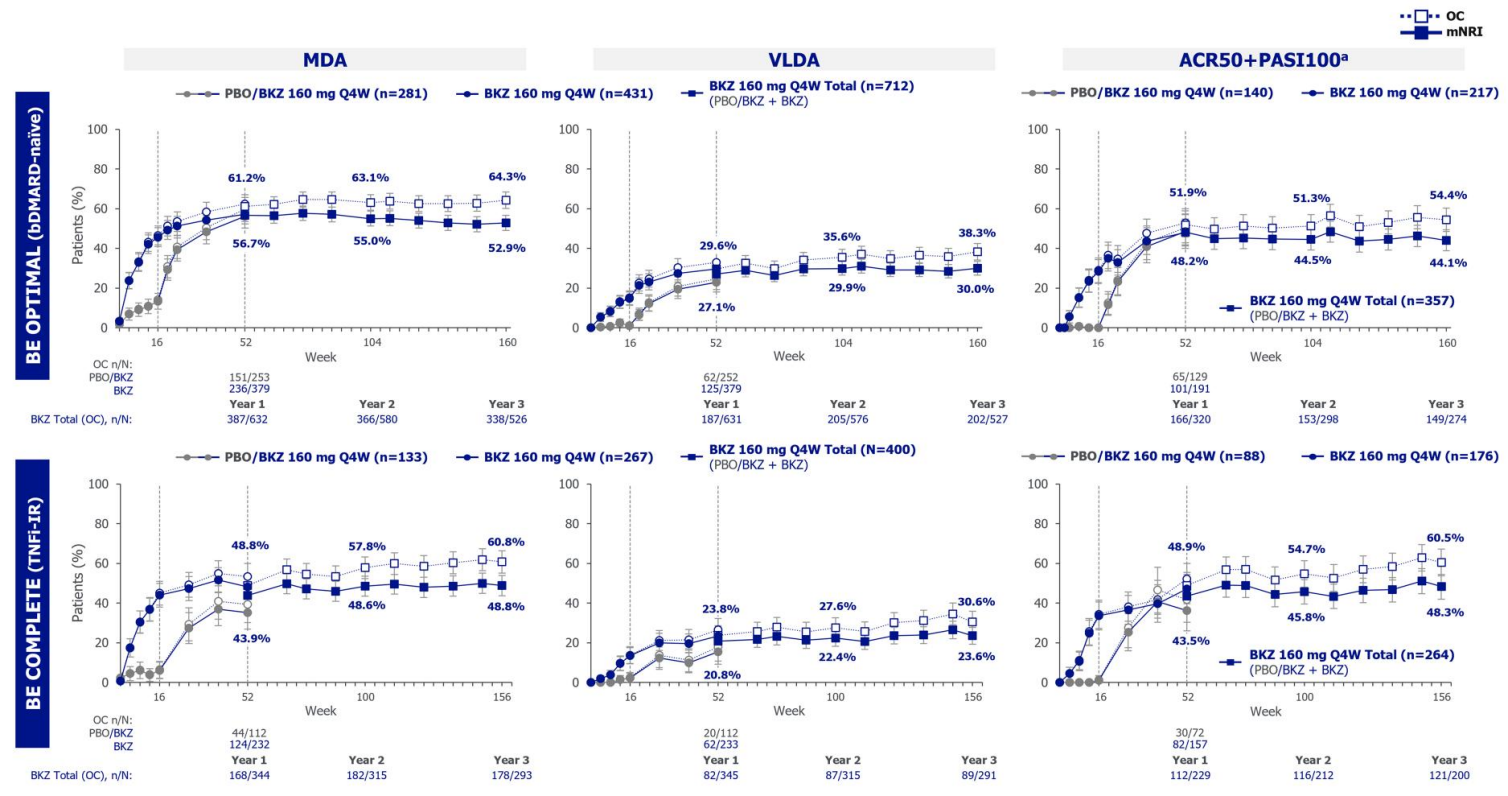
Composite outcomes to 3 years (mNRI, OC). Randomized set. Bimekizumab Total group includes bimekizumab-randomized patients and placebo-randomized patients who switched to bimekizumab at week 16. Data reported to 3 years (week 160 in BE OPTIMAL and week 156 in BE COMPLETE). mNRI considered all visits following discontinuation due to AEs or lack of efficacy as non-response; all other missing data were imputed with multiple imputation and the response derived from the imputed values. Error bars represent 95% CIs. ^a^In patients with ≥3% BSA at baseline. ACR50+PASI100: ≥50% improvement from baseline in ACR response criteria+100% improvement from baseline in Psoriasis Area and Severity Index; AE: adverse event; bDMARD: biologic DMARD; BKZ: bimekizumab; BSA: body surface area; MDA: minimal disease activity; mNRI: modified non-responder imputation; OC: observed case; PBO: placebo; Q4W: every 4 weeks; TNFi-IR: prior inadequate response or intolerance to TNF inhibitors; VLDA: very low disease activity

The proportions of bDMARD-naïve or TNFi-IR patients in the Bimekizumab Total groups achieving clinically important outcomes were sustained from 1 to 3 years, including ACR20/50/70, SJC resolution, PASI75/90/100, mNAPSI resolution, MDA, VLDA, and ACR50+PASI100 (Figs 1–3, Supplementary Fig. S4; mNRI; OC data also shown). Proportions of bDMARD-naïve and TNFi-IR patients achieving ACR50 were 56.1% and 50.4% at year 1 and 53.2% and 55.2% at year 3, respectively. Proportions achieving SJC resolution were 61.8% and 58.2% at year 1 and 59.5% and 59.1% at year 3, respectively (Fig. 1; mNRI; OC data also shown). Similarly, 64.7% and 66.2% of bDMARD-naïve and TNFi-IR patients achieved PASI100 at year 1, sustained to 61.9% and 67.5% at year 3, respectively (Fig. 2; mNRI; OC data also shown).

**Figure 4:**
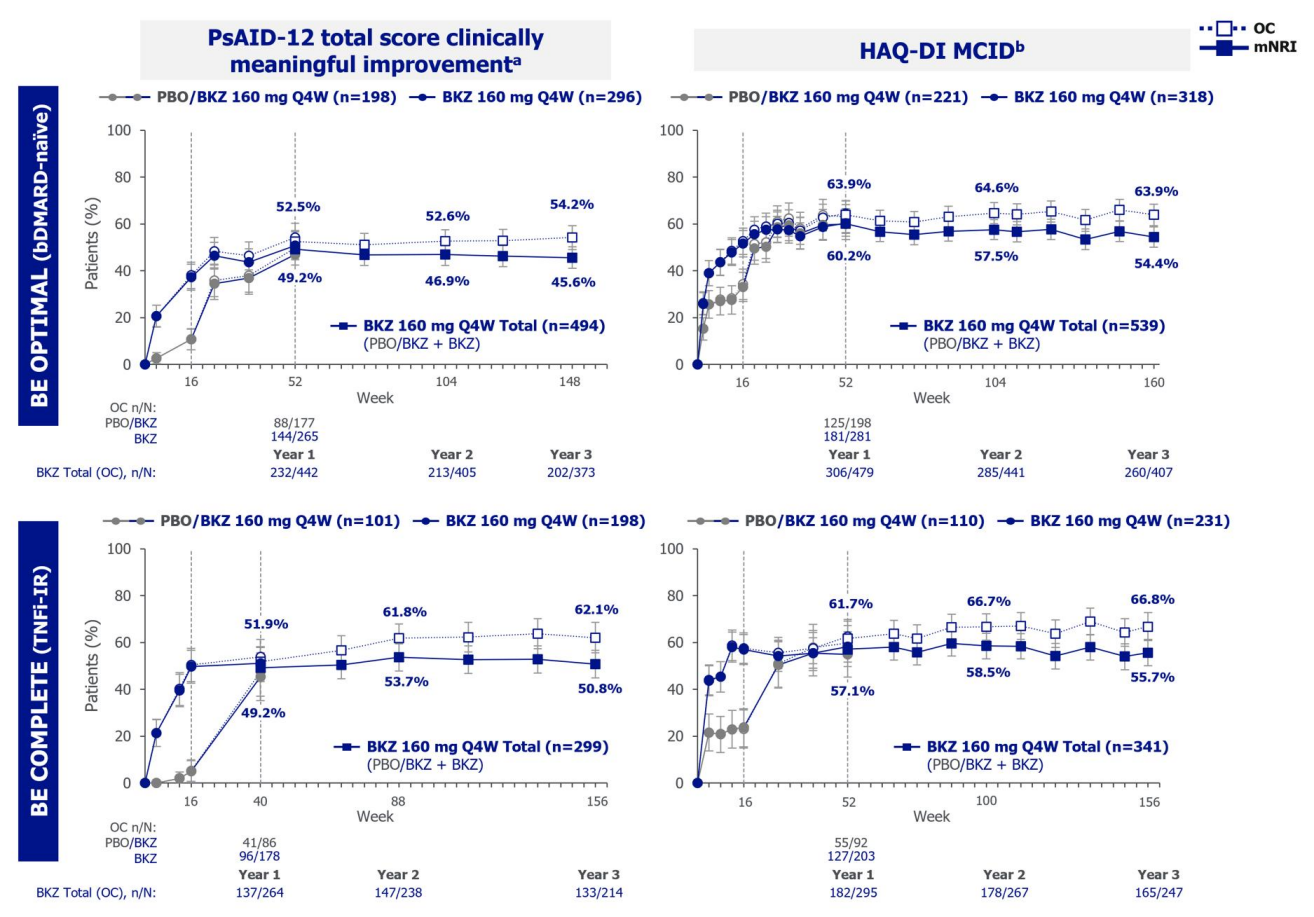
Patient-reported outcomes to 3 years (mNRI, OC). Randomized set. Bimekizumab Total group includes bimekizumab-randomized patients and placebo-randomized patients who switched to bimekizumab at week 16. Data reported to 3 years (week 148 for PsAID-12 or week 160 for HAQ-DI in BE OPTIMAL and week 156 in BE COMPLETE). mNRI considered all visits following discontinuation due to AEs or lack of efficacy as non-response; all other missing data were imputed with multiple imputation and the response derived from the imputed values. Error bars represent 95% CIs. ^a^PsAID-12 clinically meaningful improvement defined as decrease from baseline of ≥3 in patients with PsAID-12 ≥3 at baseline; data reported at week 40 (year 1) and week 88 (year 2) for BE COMPLETE; ^b^HAQ-DI MCID defined as decrease from baseline of ≥0.35 in patients with HAQ-DI of ≥0.35 at baseline; data reported at week 100 (year 2) for BE COMPLETE. AE: adverse event; bDMARD: biologic DMARD; BKZ: bimekizumab; HAQ-DI: Health Assessment Questionnaire-Disability Index; MCID: minimal clinically important difference; mNRI: modified non-responder imputation; OC: observed case; PBO: placebo; PsAID-12: Psoriatic Arthritis Impact of Disease 12-item questionnaire; Q4W: every 4 weeks; TNFi-IR: prior inadequate response or intolerance to TNF inhibitors

The proportions of patients in the Bimekizumab Total group achieving clinically meaningful improvements in PROs, including PsAID-12, HAQ-DI (Fig. 4; mNRI; OC data also shown), and pain (pain VAS ≥50% improvement; Table 2; mNRI/OC), as well as enthesitis and dactylitis resolution, and DAPSA and PASDAS REM/LDA, were also sustained from 1 to 3 years in both bDMARD-naïve and TNFi-IR patients (Table 2, Supplementary Figs S5–S6; mNRI/NRI/MI; OC data also shown).

**Table 2:** Additional efficacy outcomes and patient-reported outcomes at 3 years (mNRI, NRI, OC).

	**BE OPTIMAL** (bDMARD-naïve)				**BE COMPLETE** (TNFi-IR)			
	BKZ 160 mg Q4W Total^a^ *n* = 712				BKZ 160 mg Q4W Total^a^ *N* = 400			
	Year 1		Year 3		Year 1		Year 3	
	mNRI, %	OC, *n*/*N* (%)	mNRI, %	OC, *n*/*N* (%)	mNRI, %	OC, *n*/*N* (%)	mNRI, %	OC, *n*/*N* (%)
LEI = 0^b^	63.1	131/185 (70.8)	59.6	120/157 (76.4)	58.9	81/117 (69.2)	59.9	79/102 (77.5)
LDI = 0^c^	83.1; NRI	74/80 (92.5)	66.3; NRI	59/61 (96.7)	85.4; NRI	41/44 (93.2)	70.8; NRI	34/36 (94.4)
hs-CRP								
Normalization (CRP <5 mg/l)	70.2	477/633 (75.4)	63.3	398/526 (75.7)	68.1	251/335 (74.9)	60.6	207/284 (72.9)
Absolute median (Q1, Q3), mg/l	2.2 (0.9, 5.4); MI	2.1 (1.0, 4.9); n=633	2.3 (0.8, 6.2); MI	2.2 (1.0, 4.9); n=526	2.6 (1.1, 6.2); MI	2.3 (1.1, 5.2); n=335	2.6 (0.9, 7.0); MI	2.3 (1.0, 5.5); n=284
PASI ≤1^d^ or BSA ≤3%	86.2; NRI	614/629 (97.6)	73.7; NRI	525/531 (98.9)	83.3; NRI	333/347 (96.0)	71.5; NRI	286/295 (96.9)
DAPSA								
LDA+REM	71.4; MI	464/628 (73.9)	71.5; MI	401/514 (78.0)	62.8; MI	156/226 (69.0)	67.3; MI	215/281 (76.5)
REM	34.4; MI	231/628 (36.8)	38.2; MI	239/514 (46.5)	29.6; MI	85/226 (37.6)	30.6; MI	106/281 (37.7)
PASDAS,^e^								
LDA+REM	69.7; MI	365/481 (75.9)	68.9; MI	392/527 (74.4)	57.8; MI	227/347 (65.4)	64.4; MI	206/280 (73.6)
REM	39.8; MI	192/481 (39.9)	41.9; MI	240/527 (45.5)	31.4; MI	117/347 (33.7)	38.1; MI	124/280 (44.3)
FACIT-Fatigue MCID^e^^,^^f^	57.2	346/572 (60.5)	51.5	288/483 (59.6)	58.4	200/330 (60.6)	54.2	171/270 (63.3)
Pain VAS^g^ ≥50% improvement	59.0	401/635 (63.1)	55.2	346/530 (65.3)	54.9	208/345 (60.3)	59.4	213/293 (72.7)

	**BE OPTIMAL** (bDMARD-naïve)				**BE COMPLETE** (TNFi-IR)			
	BKZ 160 mg Q4W Total^a^ *n* = 712				BKZ 160 mg Q4W Total^a^ *N* = 400			
	Year 1		Year 3		Year 1		Year 3	
	**MI, mean (SE)**				**MI, mean (SE)**			
SJC CfB	−7.7 (0.2)		−7.9 (0.2)		−8.1 (0.4)		−8.5 (0.4)	
TJC CfB	−12.3 (0.4)		−12.7 (0.4)		−13.4 (0.6)		−13.9 (0.7)	
mNAPSI CfB^h^	−3.5 (0.1)		−3.5 (0.1)		−3.6 (0.2)		−3.7 (0.2)	
BASDAI total score CfB^i^	−3.1 (0.1)		−3.1 (0.1)		−2.9 (0.1)		−3.1 (0.2)	
HAQ-DI CfB	−0.35 (0.02)		−0.33 (0.02)		−0.37 (0.03)		−0.41 (0.03)	
FACIT-Fatigue CfB^e^	5.4 (0.3)		4.5 (0.4)		5.4 (0.5)		5.3 (0.5)	
Pain VAS CfB^g^	−30.9 (1.1)		−30.9 (1.1)		−30.1 (1.5)		−32.7 (1.6)	
PsAID-12 total score CfB^e^	−2.3 (0.1)		−2.2 (0.1)		−2.4 (0.1)		−2.4 (0.1)	

Randomized set. Data reported at 3 years (week 160 in BE OPTIMAL and week 156 in BE COMPLETE) unless otherwise stated. mNRI and OC data reported unless otherwise stated. mNRI considered all visits following discontinuation due to AEs or lack of efficacy as non-response; all other missing data were imputed with multiple imputation and the response derived from the imputed values. In cases where MI did not converge and mNRI was not available, missing data were imputed using NRI.

a Bimekizumab Total group includes bimekizumab-randomized patients and placebo-randomized patients who switched to bimekizumab at week 16;

b In patients with enthesitis at baseline (LEI >0; BE OPTIMAL: *n* = 213; BE COMPLETE: *n* = 142);

c In patients with dactylitis at baseline (LDI >0; BE OPTIMAL *n* = 89; BE COMPLETE: *n* = 48);

d For patients with psoriasis affecting ≥3% BSA at baseline;

e Data reported at week 148 for year 3 in BE OPTIMAL and week 40 for year 1 in BE COMPLETE;

f FACIT-Fatigue MCID defined as an increase from baseline of ≥4 in patients with FACIT-Fatigue subscale ≤48 at baseline (BE OPTIMAL: *n* = 643; BE COMPLETE: *n* = 371);

g Pain VAS assessed using the Patient’s Assessment of Arthritis Pain VAS which ranges from 0 to 100, 0 representing ‘no pain’ and 100 ‘most severe pain’: pain VAS ≥50% represents a substantial improvement in patient-reported pain [28];

h In patients with nail psoriasis at baseline (mNAPSI >0; BE OPTIMAL: *n* = 400; BE COMPLETE: *n* = 242);

i In patients with a BASDAI total score of ≥4 at baseline (BE OPTIMAL: *n* = 524; BE COMPLETE: *n* = 300).

AE: adverse event; bDMARD: biologic DMARD; BKZ: bimekizumab; BSA: body surface area; CfB: change from baseline; DAPSA: Disease Activity in Psoriatic Arthritis; FACIT-Fatigue: Functional Assessment of Chronic Illness Therapy − Fatigue; HAQ-DI: Health Assessment Questionnaire-Disability Index; hs-CRP: high sensitivity CRP; LDA: low disease activity; LDI: Leeds Dactylitis Index; LEI: Leeds Enthesitis Index; MCID: minimal clinically important difference; MI: multiple imputation; mNAPSI: modified Nail Psoriasis Severity Index; mNRI: modified NRI; NRI: non-responder imputation; OC: observed case; PASDAS: Psoriatic Disease Activity Score; PASI: Psoriasis Area and Severity Index; PBO: placebo; PsAID-12: Psoriatic Arthritis Impact of Disease 12-item questionnaire; Q1: quartile 1; Q3: quartile 3; Q4W: every 4 weeks; REM: remission; SJC: swollen joint count; TJC: tender joint count; TNFi-IR: prior inadequate response or intolerance to TNF inhibitors; VAS: visual analogue scale.

In BE OPTIMAL, patients in the reference arm who switched to bimekizumab treatment at week 52 demonstrated similar sustained clinical responses across all efficacy outcomes to 3 years (reflecting 2 years of bimekizumab treatment) (Supplementary Table S7). Responses from 1 to 3 years were consistent across patients who switched from the reference arm to bimekizumab at week 52 and patients in the Bimekizumab Total group.

Efficacy data imputed using NRI can be found in Supplementary Figs S7–S10.

## Discussion

Results from the BE VITAL OLE demonstrated that bimekizumab treatment maintained a tolerable safety profile, while efficacy seen at year 1 and year 2 continued to be sustained up to 3 years in patients with active PsA who were bDMARD-naïve or TNFi-IR. Efficacy for patients treated with bimekizumab from baseline or week 16 was sustained across a range of PsA domains, including joints, skin, and nails, and was reflected in stringent composite outcomes such as MDA.

The longer-term safety profile, observed to 3 years, was generally consistent between bDMARD-naïve and TNFi-IR patients. Bimekizumab was well tolerated in patients with PsA, with no new safety signals observed with longer-term treatment compared with previous reports [12–14]. Incidence rates of TEAEs decreased with each year of exposure, and there were low incidences of TEAEs of interest, such as suicidal ideation and behaviour, uveitis, MACE, IBD, serious hypersensitivity, and elevated liver enzymes. Fungal infections were mostly mild or moderate, and none were systemic; the incidence of fungal infections did not increase with longer-term therapy. The incidence of *Candida* infections decreased each year and the incidence rate of study discontinuation due to *Candida* infections was low. This was consistent across both studies and aligns with longer-term follow-up data from phase 3 trials of bimekizumab in patients with psoriasis or axial SpA [32, 33].

Bimekizumab treatment resulted in consistent and sustained clinical efficacy to 3 years in bDMARD-naive and TNFi-IR patients with active PsA. The rapid improvements observed at week 16, and sustained or increased to year 1, remained consistent out to 3 years. Sustained improvements in skin outcomes for up to 3 years of bimekizumab treatment are notable, with the majority of patients achieving PASI100 at year 3 (BE OPTIMAL: 61.9%; BE COMPLETE: 67.5%). Previous trials have reported that IL-17A-specific inhibitors, in comparison with IL-23 inhibitors, lose efficacy in skin outcomes over time in patients with psoriatic disease, often leading to treatment discontinuation or cycling through multiple therapies [34–37]. Therefore, the sustained improvements in skin outcomes with bimekizumab treatment could be hypothesized to be due to the addition of IL-17F inhibition. Further research into the potential difference in sustained skin efficacy between IL-17A inhibitors and IL-17A/F inhibitors would be of value to support this hypothesis. Furthermore, patients with PsA who demonstrate simultaneous improvement in joint and skin symptoms following treatment have been shown to have greater overall treatment outcomes [38].

In the present analysis, the proportions of patients achieving composite measures of disease activity were sustained up to 3 years, with a relatively high proportion of patients achieving VLDA (BE OPTIMAL: 30.0%, BE COMPLETE: 23.6%), suggesting stringent disease control. Similar improvements in PROs and disease impact were observed to 3 years as previously reported at year 2 [13].

The consistent efficacy response observed to 3 years of bimekizumab treatment regardless of prior TNFi exposure is also an important finding, as clinical trial and registry data from patients with PsA treated with other bDMARDs, including selective IL-17A inhibitors, showed that TNFi-experienced patients were less likely to achieve treatment response targets than TNFi-naive patients [39, 40]. The sustained response in both bDMARD-naïve and TNFi-experienced patients reported here may be due to the inhibition of IL-17F in addition to IL-17A. Further research into the contribution of IL-17F inhibition to the consistent and sustained efficacy of bimekizumab in TNFi-naïve and TNFi-experienced patients would be of value to examine the hypothesis that long-term efficacy is improved with combined inhibition of IL-17F and IL-17A, compared with IL-17A inhibition alone.

bDMARD-naïve patients switching from reference arm treatment to bimekizumab at week 52 in BE OPTIMAL showed sustained efficacy in joint symptoms and improved efficacy in skin symptoms at up to 2 years after the switch, reflecting the improvements seen in patients receiving bimekizumab from baseline at 2 years [13].

### Strengths

In both studies, a high proportion of patients remained in the study to 3 years. While clinical trial retention rates are not directly comparable with drug persistence data from real-world clinical practice as they are influenced by different factors, the high retention rate in this study is noteworthy given the median persistence of biologic treatments in PsA is often reported to be <3 years [41–44]. BE VITAL evaluated bimekizumab treatment in both bDMARD-naïve and TNFi-IR populations over a longer-term treatment duration, with consistent responses observed in both populations. Furthermore, in BE OPTIMAL, patients who switched from the reference treatment to bimekizumab also maintained robust long-term efficacy responses with no additional safety signals observed. Importantly, this study enabled the evaluation of safety through 3 years from baseline randomization. The reporting of SJC resolution, a clinical measure of inflammation, alongside broad clinical and PROs to 3 years also provides further evidence to support the role of bimekizumab treatment in the long-term control of inflammation and achievement of outcomes important to patients.

### Limitations

BE COMPLETE was open-label from week 16, while BE OPTIMAL was open-label from week 52, so these studies are not directly comparable due to the difference in the blinding duration. However, both patient groups received open-label treatment for at least 2 years. The patient populations in BE OPTIMAL and BE COMPLETE also tended to have more severe disease compared with patients with PsA attending routine clinical practice, highlighting the need to collect real-world data to corroborate these findings in clinical practice [45].

While the proportions of patients remaining in these studies to 3 years were relatively high, the loss of patients to follow-up and subsequent role of imputation for missing values is greater at increasingly longer time points in clinical trials. As some patients discontinued treatment due to lack of efficacy or AEs, the study population at 3 years may have been responder-enriched, meaning patients who responded well and tolerated the treatment were more likely to have remained in the study. This can result in survivor bias, which is often seen in long-term clinical trials. However, the use of the mNRI method to account for missing binary data, which considers patients who discontinued due to lack of efficacy or AEs as non-responders, somewhat accounts for this survivor bias. The majority of the data reported in this study are binary and use the mNRI method. For missing continuous data, the MI method was used, which assumes data are missing at random and creates multiple plausible datasets from the observed data, analyzes each dataset separately, and combines the results to account for both within- and between-imputation uncertainty under missing-at-random assumptions. While this is a less conservative imputation method and may not reflect all potential reasons for the missing data, there is a limited amount of continuous data reported in this study, so further sensitivity analyses would be unlikely to affect the interpretation of the results.

### Future directions

Evaluation of the long-term safety and efficacy of bimekizumab in a real-world clinical setting would be valuable, alongside the examination of safety and efficacy in different patient subgroups, such as ‘difficult-to-treat’ patients [46] and patients with different disease durations, comorbidities, or co-medications. Future comparative trials, as well as further examination of the association between early resolution of inflammation, meaningful sustained improvements in patient-reported symptoms, prevention of structural progression and cumulative disease burden, would also be of value.

## Conclusions

Bimekizumab was well tolerated, with a safety profile consistent with that previously observed in studies of bimekizumab treatment in PsA during phase 3 studies [12–14]. Bimekizumab treatment resulted in sustained and consistent high levels of efficacy responses across the full spectrum of disease over 3 years in both bDMARD-naïve and TNFi-IR patients. These data support the suitability of bimekizumab for the long-term treatment of bDMARD-naïve and TNFi-IR patients with PsA.

## Supplementary Material

keag118_Supplementary_Data

## Data Availability

Data from this manuscript may be requested by qualified researchers 6 months after product approval in the USA and/or Europe, or global development is discontinued, and 18 months after trial completion. Investigators may request access to anonymized individual patient data and redacted study documents, which may include: raw datasets, analysis-ready datasets, study protocol, blank case report form, annotated case report form, statistical analysis plan, dataset specifications, and clinical study report. Prior to use of the data, proposals need to be approved by an independent review panel at www.Vivli.org and a signed data sharing agreement will need to be executed. All documents are available in English only, for a pre-specified time, typically 12 months, on a password-protected portal.
