# Supplementary material for: Bimekizumab safety and efficacy in patients with psoriatic arthritis: 3-year results from two phase 3 studies
Source: Rheumatology (Oxford). 2026 Mar 16;65(5):keag118. doi: 10.1093/rheumatology/keag118 (PMC13198880; doi:10.1093/rheumatology/keag118)
Supplement: keag118_Supplementary_Data [file keag118_supplementary_data.zip › rhe-25-2713-File003.docx]

**SUPPLEMENTARY APPENDIX**

**Supplementary Figure S1.** BE OPTIMAL and BE COMPLETE study designs


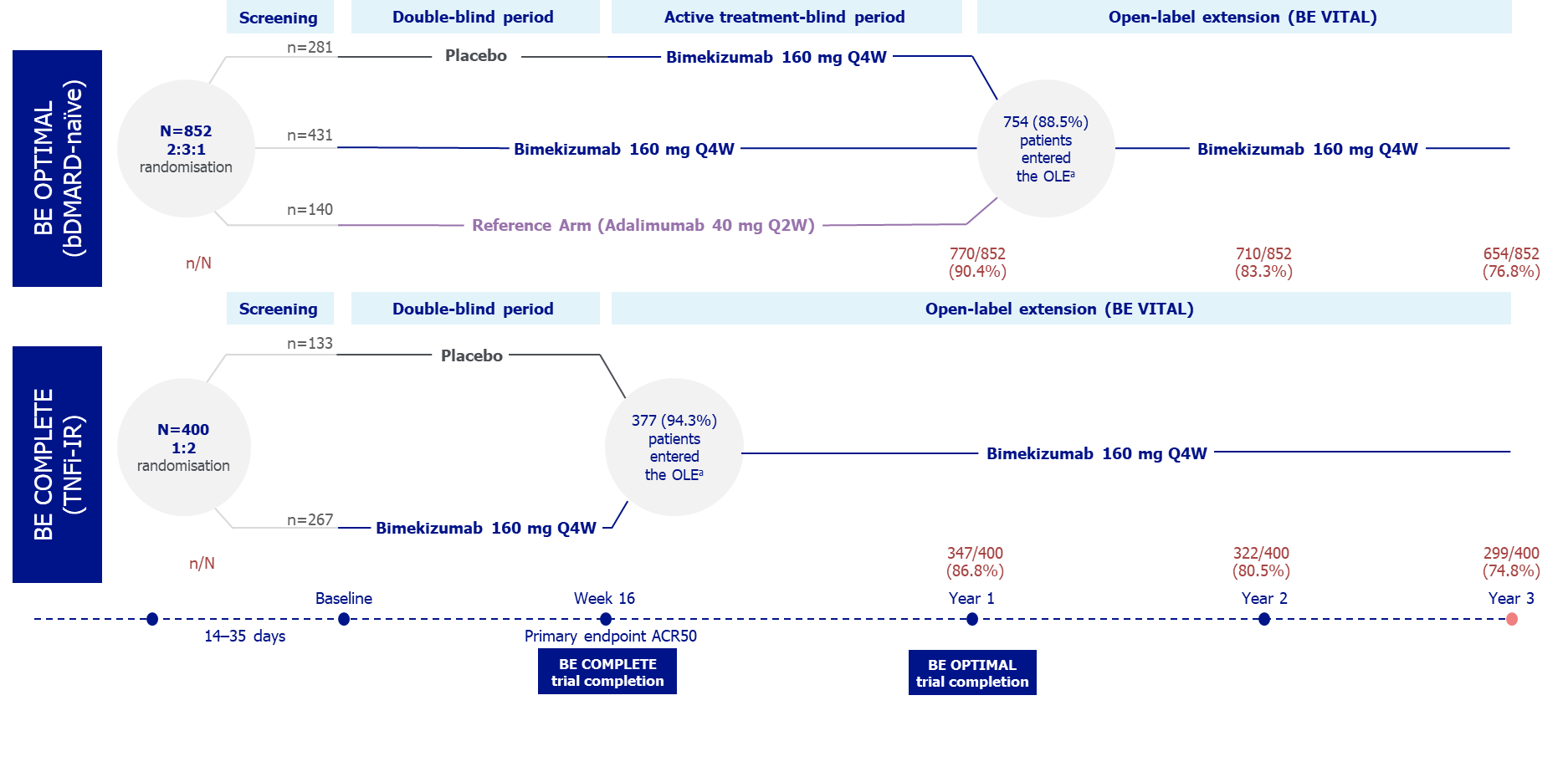


For BE OPTIMAL, the adalimumab 40 mg Q2W treatment arm served as an active reference. The BE OPTIMAL study was not powered for statistical comparisons of adalimumab to bimekizumab or placebo. Completion rates include patients that completed to Week 52/104/160 in BE OPTIMAL and Week 52/100/156 in BE COMPLETE not on randomized treatment (BE OPTIMAL Week 52: n=9 [1.1%], Week 104: n=8 [0.9%], Week 160: n=10 [1.2%]; BE COMPLETE Week 52: n=4 [1.0%], Week 100: n=2 [0.5%], Week 156: n=4 [1.0%]). 2 (0.5%) patients in BE COMPLETE were classified as ongoing at Week 52 as they did not have a visit for Week 52, but no formal discontinuation reason was reported. Permitted concomitant medications included, and were not limited to, non-steroidal anti-inflammatory drugs and conventional synthetic DMARDs within protocol guidelines; changes to permitted concomitant medications were allowed at any time after enrolment in BE VITAL, if deemed appropriate by the investigator. **[a]** Safety follow-up visit 20 weeks after the last dose for patients not enrolling in the extension study. **ACR50:** ≥50% improvement from baseline in American College of Rheumatology response criteria; **bDMARD:** biologic DMARD; **DMARD:** disease modifying anti‑rheumatic drug; **OLE:** open-label extension; **Q2W:** every 2 weeks; **Q4W:** every 4 weeks; **TNFi-IR:** prior inadequate response or intolerance to tumour necrosis factor inhibitors.

**Supplementary Figure S2.** Patient disposition in BE OPTIMAL and BE COMPLETE

1. BE OPTIMAL


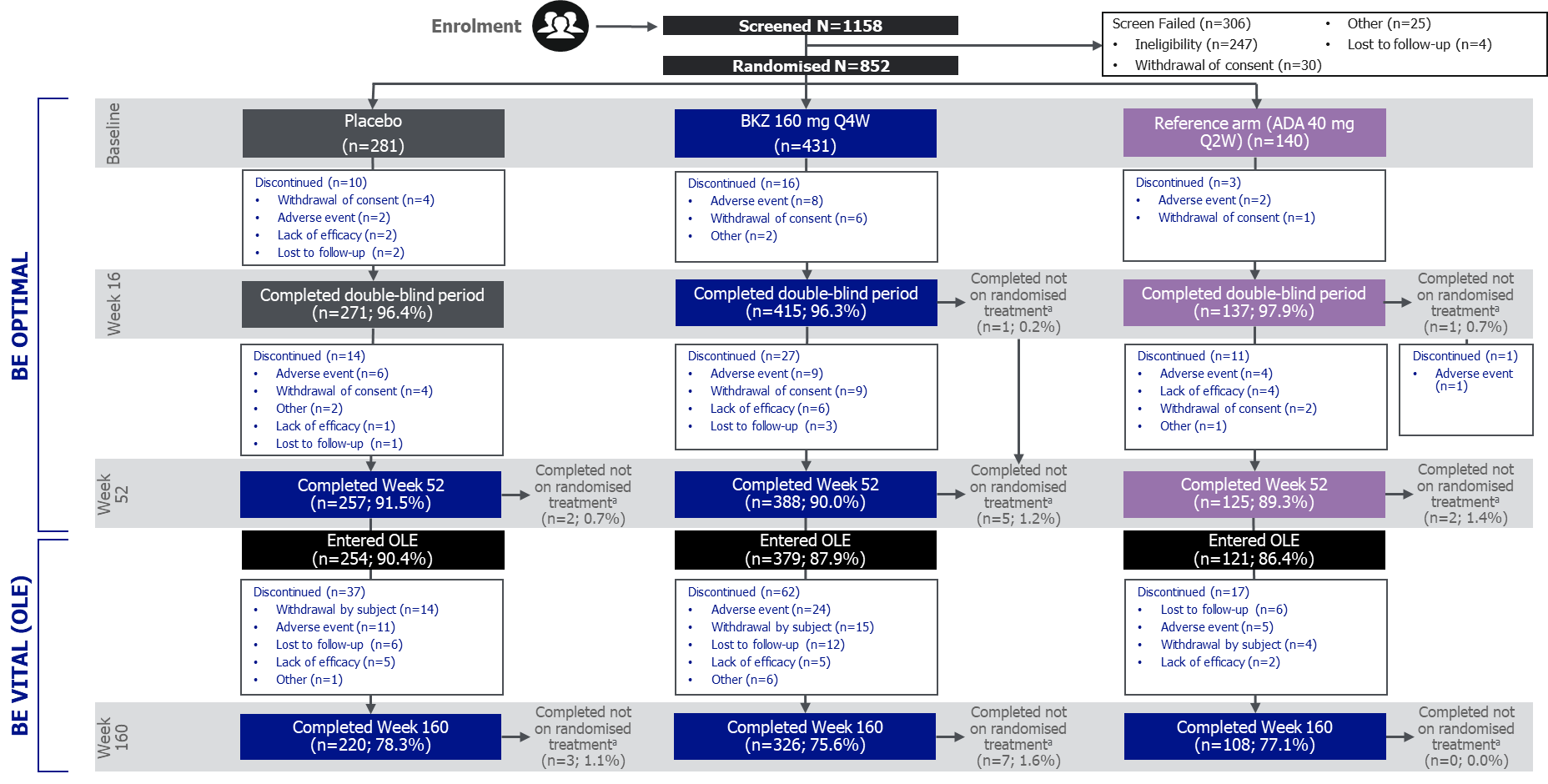


1. BE COMPLETE


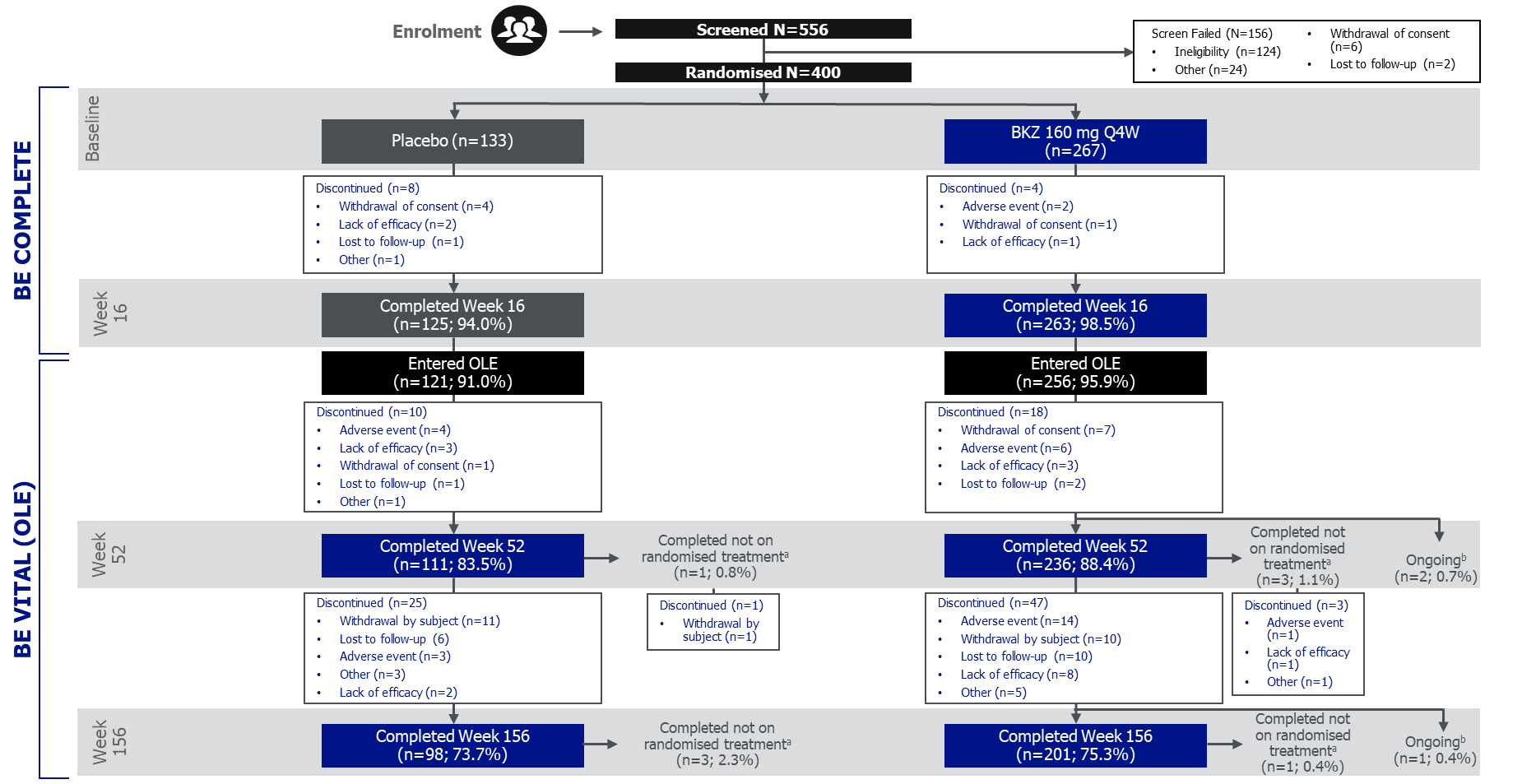


Patients who discontinued treatment but completed safety follow-up period: BE OPTIMAL placebo (n=25/61, 41.0%), bimekizumab (n=50/105, 47.6%), adalimumab (n=18/32, 56.3%); BE COMPLETE placebo (n=14/35, 40.0%); bimekizumab (n=31/65, 47.7%). Safety reported to Week 156. **[a]** Patients who withdrew from the study treatment or deviated from assigned randomized treatment but returned for all scheduled visits up to and including Week 16/52/160/156 were considered as having completed the treatment period not on randomized treatment; **[b]** Ongoing includes patients who did not have a Week 52/156 visit and no visits after in the BE VITAL OLE but have not discontinued within the timeframe. **ADA:** ‍adalimumab; **BKZ:** bimekizumab; **OLE:** ‍open-‍label extension.

**Supplementary Table S1.** Baseline demographics and disease characteristics

|  | **BE OPTIMAL**  (bDMARD-naïve) | **BE COMPLETE**  (TNFi-IR) |
| --- | --- | --- |
|  | **BKZ 160 mg Q4W Total^a^**  **(n=712)** | **BKZ 160 mg Q4W Total^a^**  **(N=400)** |
| **Patient demographics** | | |
| Age, years, mean (SD) | 48.6 (12.2) | 50.5 (12.5) |
| Sex, male, n (%) | 328 (46.1) | 190 (47.5) |
| BMI, kg/m^2^, mean (SD) | 29.4 (6.5) | 29.8 (6.2) |
| **Disease characteristics** | | |
| Time since PsA diagnosis,^b^ years, mean (SD) | 5.8 (7.0) | 9.5 (9.3) |
| Any csDMARD at baseline, n (%)  Concomitant methotrexate, n (%) | 495 (69.5)  415 (58.3) | 202 (50.5)  170 (42.5) |
| Prior TNFi exposure, n (%)  Inadequate response to 1 TNFi  Inadequate response to 2 TNFi  Intolerance to TNFi | -  -  - | 306 (76.5)  45 (11.3)  49 (12.3) |
| SJC (of 66 joints), mean (SD) | 9.2 (6.6) | 9.9 (7.7) |
| TJC (of 68 joints), mean (SD) | 16.9 (12.1) | 18.7 (13.8) |
| hs-CRP, mg/L  Median (Q1, Q3)  Normalization (CRP <5 mg/L), n (%) | 4.1 (1.5, 11.0)  399 (56.0) | 4.9 (1.8, 14.8)  203 (50.8) |
| ≥3% BSA affected by psoriasis, n (%) | 357 (50.1) | 264 (66.0) |
| PASI score,^c^ mean (SD) | 8.1 (6.4) | 9.6 (8.4) |
| Enthesitis (LEI >0),^d^ n (%)  LEI score,^e^ mean (SD) | 213 (29.9)  2.6 (1.5) | 142 (35.5)  2.7 (1.5) |
| Dactylitis (LDI >0),^f^ n (%)  LDI score,^g^ mean (SD) | 89 (12.5)  47.0 (49.6) | 48 (12.0)  70.9 (117.0) |
| Nail psoriasis (mNAPSI >0),^f^ n (%)  mNAPSI score,^h^ mean (SD) | 400 (56.2)  4.1 (2.3) | 242 (60.5)  4.4 (2.8) |
| HAQ-DI score,^i^ mean (SD) | 0.85 (0.59) | 0.99 (0.62) |
| BASDAI total score,^j^ mean (SD) | 6.2 (1.3) | 6.3 (1.3) |
| PhGA-PsA,^d^ mean (SD) | 57.3 (15.8) | 58.7 (17.8) |
| PGA-PsA,^i^ mean (SD) | 56.1 (23.5) | 61.4 (22.3) |
| Pain VAS,^i,k^ mean (SD) | 54.9 (23.9) | 59.5 (24.3) |
| PsAID-12 total score,^i^ mean (SD) | 4.0 (1.9) | 4.5 (2.0) |
| FACIT-Fatigue,^i^ mean (SD) | 37.1 (9.9) | 35.6 (10.3) |
| DAPSA,^i^ mean (SD) | 38.1 (18.8) | 41.7 (22.0) |
| PASDAS,^l^ mean (SD) | 5.3 (1.0) | 5.5 (1.0) |

Randomized set. **[a]** Bimekizumab Total group includes bimekizumab-randomized patients and placebo‑randomized patients who switched to bimekizumab at Week 16; **[b]** Data missing for 10 patients in BE OPTIMAL and 2 patients in BE COMPLETE; **[c]** In patients with ≥3% BSA affected by psoriasis at baseline (BE OPTIMAL: n=357; BE COMPLETE: n=264); **[d]** Data missing for 6 patients in BE OPTIMAL and 1 patient in BE COMPLETE; **[e]** In patients with enthesitis (LEI >0) at baseline; **[f]** Data missing for 7 patients in BE OPTIMAL and 1 patient in BE COMPLETE; **[g]** In patients with dactylitis (LDI >0) at baseline; **[h]** In patients with nail psoriasis (mNAPSI >0) at baseline; **[i]** Data missing for 1 patient in BE OPTIMAL; **[j]** In patients with a BASDAI total score ≥4 at baseline (BE OPTIMAL: n=524; BE COMPLETE: n=300); **[k]** Pain VAS assessed using the Patient’s Assessment of Arthritis Pain VAS which ranges from 0 to 100, 0 representing ‘no pain’ and 100 ‘most severe pain’; **[l]** Data missing for 8 patients in BE OPTIMAL and 1 patient in BE COMPLETE. **BASDAI:** Bath Ankylosing Spondylitis Disease Activity Index; **bDMARD:** biologic disease-modifying antirheumatic drug; **BKZ:**bimekizumab; **BMI:** body mass index; **BSA:** body surface area; **CRP:** C‑reactive protein; **csDMARD:** conventional synthetic disease-modifying antirheumatic drug; **DAPSA:** Disease Activity Index for Psoriatic Arthritis; **FACIT-Fatigue:** Functional Assessment of Chronic Illness Therapy-Fatigue; **HAQ-DI:** Health Assessment Questionnaire-Disability Index; **hs‑CRP:** high sensitivity C-reactive protein; **LDI:** Leeds Dactylitis Index; **LEI:** Leeds Enthesitis Index; **mNAPSI:** modified Nail Psoriasis Severity Index; **PASDAS:** Psoriatic Arthritis Disease Activity Score; **PASI:** Psoriasis Area and Severity Index; **PBO:** placebo; **PGA-PsA:** Patient’s Global Assessment of Psoriatic Arthritis; **PhGA-PsA:** Physician’s Global Assessment of Psoriatic Arthritis; **PsA:** psoriatic arthritis; **PsAID‑12:** Psoriatic Arthritis Impact of Disease 12‑item questionnaire; **Q1:** quartile 1; **Q3:** quartile 3; **Q4W:** every four weeks; **SD:** standard deviation; **SJC:** swollen joint count; **TJC:** tender joint count; **TNFi:** tumour necrosis factor inhibitors; **TNFi-IR:** prior inadequate response or intolerance to TNFi; **VAS:** visual analogue scale.

**Supplementary Table S2.** Safety overview to 3 years, split by year

|  | **BE OPTIMAL** (bDMARD-naïve) | | | **BE COMPLETE** (TNFi-IR) | | |
| --- | --- | --- | --- | --- | --- | --- |
|  | **Year 1** | **Year 2** | **Year 3** | **Year 1** | **Year 2** | **Year 3** |
| **n^a^ (%)**  **EAIR/100 PY (95% CI)** | **BKZ 160 mg Q4W Total^b^  n=702**  **(595.6 PY)** | **BKZ 160 mg Q4W Total^b^**  **n=652**  **(620.8 PY)** | **BKZ 160 mg Q4W Total^b^**  **n=607  (573.5 PY)** | **BKZ 160 mg Q4W Total^b^**  **N=388  (340.0 PY)** | **BKZ 160 mg Q4W Total^b^**  **N=361 (335.2 PY)** | **BKZ 160 mg Q4W Total^b^**  **N=329 (307.9 PY)** |
| Any TEAE | 554 (78.9)  223.0 (204.8–242.3) | 456 (69.9)  151.0 (137.5–165.5) | 410 (67.5)  138.8 (125.7–152.9) | 244 (62.9)  126.3 (110.9–143.1) | 191 (52.9)  87.8 (75.8–101.2) | 168 (51.1)  79.6 (68.1–92.6) |
| Serious TEAEs^c^ | 45 (6.4)  7.8 (5.7–10.4) | 53 (8.1)  8.9 (6.7–11.7) | 38 (6.3)  6.9 (4.8–9.4) | 23 (5.9)  7.0 (4.4–10.4) | 16 (4.4)  4.9 (2.8–7.9) | 21 (6.4)  7.0 (4.3–10.7) |
| Study discontinuations due to TEAEs | 24 (3.4)  4.1 (2.6–6.1) | 20 (3.1)  3.3 (2.0–5.0) | 17 (2.8)  3.0 (1.7–4.8) | 18 (4.6)  5.4 (3.2–8.5) | 6 (1.7)  1.8 (0.7–3.9) | 3 (0.9)  1.0 (0.2–2.9) |
| Permanent treatment discontinuations due to TEAEs | 27 (3.8)  4.6 (3.0–6.7) | 23 (3.5)  3.8 (2.4–5.6) | 16 (2.6)  2.8 (1.6–4.6) | 19 (4.9)  5.7 (3.4–8.9) | 7 (1.9)  2.1 (0.8–4.3) | 3 (0.9)  1.0 (0.2–2.9) |
| Drug-related  TEAEs | 222 (31.6)  47.8 (41.7–54.5) | 150 (23.0)  28.5 (24.2–33.5) | 109 (18.0)  21.3 (17.5–25.7) | 84 (21.6)  29.0 (23.2–36.0) | 52 (14.4)  16.9 (12.6–22.2) | 47 (14.3)  16.8 (12.4–22.4) |
| Severe TEAEs | 23 (3.3)  3.9 (2.5–5.9) | 24 (3.7)  3.9 (2.5–5.8) | 19 (3.1)  3.4 (2.0–5.3) | 16 (4.1)  4.8 (2.7–7.8) | 9 (2.5)  2.7 (1.2–5.1) | 14 (4.3)  4.7 (2.5–7.8) |
| Deaths | 1 (0.1)^d^  0.2 (0.0–0.9) | 0 | 1 (0.2)^e^  0.2 (0.0–1.0) | 1 (0.3)^f^  0.3 (0.0–1.6) | 0 | 0 |
| Most frequently reported TEAEs (5 most common TEAEs in any BKZ-treated group at the Year 3 data cut) | | | | | | |
| SARS-CoV-2 (COVID-19) infection^g^ | 21 (3.0)  3.6 (2.2–5.4) | 114 (17.5)  20.5 (16.9–24.6) | 88 (14.5)  16.9 (13.6–20.8) | 29 (7.5)  8.8 (5.9–12.6) | 25 (6.9)  7.7 (5.0–11.3) | 26 (7.9)  8.8 (5.8–13.0) |
| Nasopharyngitis | 84 (12.0)  15.4 (12.3–19.1) | 32 (4.9)  5.3 (3.6–7.5) | 40 (6.6)  7.3 (5.2–10.0) | 23 (5.9)  7.0 (4.5–10.6) | 14 (3.9)  4.3 (2.3–7.2) | 14 (4.3)  4.7 (2.6–7.8) |
| Upper respiratory tract infection | 50 (7.1)  8.8 (6.6–11.6) | 38 (5.8)  6.3 (4.5–8.6) | 33 (5.4)  6.0 (4.1–8.4) | 12 (3.1)  3.6 (1.9–6.3) | 15 (4.2)  4.6 (2.6–7.6) | 14 (4.3)  4.6 (2.5–7.8) |
| Oral candidiasis | 39 (5.6)  6.7 (4.8–9.2) | 35 (5.4)  5.8 (4.1–8.1) | 24 (4.0)  4.3 (2.7–6.4) | 25 (6.4)  7.6 (4.9–11.3) | 11 (3.0)  3.3 (1.7–5.9) | 9 (2.7)  3.0 (1.4–5.6) |
| Urinary tract infection | 42 (6.0)  7.3 (5.2–9.8) | 25 (3.8)  4.1 (2.7–6.1) | 36 (5.9)  6.5 (4.6–9.0) | 23 (5.9)  7.0 (4.5–10.5) | 13 (3.6)  4.0 (2.1–6.8) | 17 (5.2)  5.7 (3.3–9.1) |
| Safety topics of interest | | | | | | |
| Serious infections | 6 (0.9)  1.0 (0.4–2.2) | 11 (1.7)  1.8 (0.9–3.2) | 10 (1.6)  1.8 (0.8–3.2) | 7 (1.8)  2.1 (0.8–4.3) | 2 (0.6)  0.6 (0.1–2.2) | 4 (1.2)  1.3 (0.4–3.3) |
| Opportunistic infections^h^ | 9 (1.3)  1.5 (0.7–2.9) | 7 (1.1)  1.1 (0.5–2.3) | 3 (0.5)  0.5 (0.1–1.5) | 2 (0.5)  0.6 (0.1–2.1) | 1 (0.3)  0.3 (0.0–1.7) | 0 |
| Active tuberculosis | 0 | 0 | 0 | 0 | 0 | 0 |
| Fungal infections | 85 (12.1)  15.3 (12.2–18.9) | 61 (9.4)  10.4 (8.0–13.4) | 45 (7.4)  8.2 (5.9–10.9) | 38 (9.8)  11.9 (8.4–16.3) | 15 (4.2)  4.6 (2.6–7.5) | 14 (4.3)  4.6 (2.5–7.8) |
| *Candida* infection | 56 (8.0)  9.8 (7.4–12.7) | 42 (6.4)  7.1 (5.1–9.5) | 29 (4.8)  5.2 (3.5–7.5) | 26 (6.7)  8.0 (5.2–11.7) | 11 (3.0)  3.3 (1.7–5.9) | 11 (3.3)  3.6 (1.8–6.5) |
| Oral candidiasis | 39 (5.6)  6.7 (4.8–9.2) | 35 (5.4)  5.8 (4.1–8.1) | 24 (4.0)  4.3 (2.7–6.4) | 25 (6.4)  7.6 (4.9–11.3) | 11 (3.0)  3.3 (1.7–5.9) | 9 (2.7)  3.0 (1.4–5.6) |
| Fungal infections NEC | 30 (4.3)  5.2 (3.5–7.4) | 20 (3.1)  3.3 (2.0–5.1) | 18 (3.0)  3.2 (1.9–5.0) | 11 (2.8)  3.3 (1.6–5.9) | 3 (0.8)  0.9 (0.2–2.6) | 4 (1.2)  1.3 (0.4–3.4) |
| Any neutropenia^i^ | 10 (1.4)  1.7 (0.8–3.1) | 9 (1.4)  1.5 (0.7–2.8) | 6 (1.0)  1.1 (0.4–2.3) | 6 (1.5)  1.8 (0.7–3.9) | 7 (1.9)  2.1 (0.8–4.3) | 2 (0.6)  0.7 (0.1–2.4) |
| Serious hypersensitivity reactions | 0 | 0 | 0 | 0 | 1 (0.3)  0.3 (0.0–1.7)^j^ | 0 |
| Any administration or injection site reaction^k^ | 16 (2.3)  2.7 (1.6–4.4) | 10 (1.5)  1.6 (0.8–3.0) | 4 (0.7)  0.7 (0.2–1.8) | 6 (1.5)  1.8 (0.7–3.9) | 1 (0.3)  0.3 (0.0–1.7) | 1 (0.3)  0.3 (0.0–1.8) |
| Elevated liver enzymes,^l^ n/N (%) | 46/702 (6.6)  8.0 (5.9–10.7) | 20/652 (3.1)  3.3 (2.0–5.1) | 19/607 (3.1)  3.4 (2.0–5.3) | 23/388 (5.9)  7.0 (4.5–10.5) | 11/361 (3.0)  3.3 (1.7–6.0) | 8/329 (2.4)  2.6 (1.1–5.2) |
| >3× ULN ALT/AST | 21/701 (3.0)  3.6 (2.2–5.5) | 11/643 (1.7)  1.8 (0.9–3.2) | 8/594 (1.3)  1.4 (0.6–2.8) | 11/388 (2.8)  3.3 (1.6–5.9) | 8/354 (2.3)  2.4 (1.0–4.8) | 5/320 (1.6)  1.6 (0.5–3.8) |
| >5× ULN ALT/AST | 5/701 (0.7)  0.8 (0.3–2.0) | 3/643 (0.5)  0.5 (0.1–1.4) | 2/594 (0.3)  0.3 (0.0–1.3) | 4/388 (1.0)  1.2 (0.3–3.0) | 3/354 (0.8)  0.9 (0.2–2.6) | 2/320 (0.6)  0.7 (0.1–2.4) |
| Adjudicated MACE | 4 (0.6)  0.7 (0.2–1.7) | 1 (0.2)  0.2 (0.0–0.9) | 3 (0.5)  0.5 (0.1–1.5) | 2 (0.5)  0.6 (0.1–2.1) | 0 | 0 |
| Malignancies,^m^ excluding nonmelanoma skin cancer | 4 (0.6)  0.7 (0.2–1.7) | 3 (0.5)  0.5 (0.1–1.4) | 2 (0.3)  0.3 (0.0–1.3) | 3 (0.8)  0.9 (0.2–2.6) | 2 (0.6)  0.6 (0.1–2.2) | 5 (1.5)  1.6 (0.5–3.8) |
| Adjudicated suicidal ideation and behaviour^n^ | 0 | 1 (0.2)  0.2 (0.0–0.9) | 1 (0.2)  0.2 (0.0–1.0) | 0 | 0 | 0 |
| Adjudicated IBD^o^ | 4 (0.6)^p^  0.7 (0.2–1.7) | 1 (0.2)  0.2 (0.0–0.9) | 1 (0.2)  0.2 (0.0–1.0) | 0 | 0 | 1 (0.3)^q^  0.3 (0.0–1.8) |
| Uveitis^r^ | 1 (0.1)  0.2 (0.0–0.9) | 0 | 3 (0.5)  0.5 (0.1–1.5) | 0 | 0 | 0 |

Safety set. Data reported at Year 1 (Weeks 0–52), Year 2 (Weeks >52–104) and Year 3 (Weeks >104–156). **[a]** ‘n’ denotes the number of patients reporting at least one of the respective TEAE; **[b]** Bimekizumab Total group includes bimekizumab-randomized patients and placebo-randomized patients that switched to bimekizumab at Week 16; includes events after switch only; **[c]** Serious TEAEs met one or more of the following criteria: death, life‑threatening event, significant or persistent disability/incapacity, congenital anomaly/birth defect (including in a foetus), important medical event, or initial inpatient hospitalization or prolonged hospitalization; **[d]** One death as previously described [13]; **[e]** One death due to cardiac arrest in a 66-year old patient randomized to bimekizumab at baseline with a history of cardiovascular disease and multiple concomitant medications, considered not related to the study drug; **[f]** Sudden death as previously described [13]; **[g]** Specific terms for SARS-CoV-2 (COVID-19) infections were not available in the MedDRA v19.0; confirmed or suspected cases were identified using the preferred terms “Coronavirus infection” and “Coronavirus test positive”; **[h]** No cases of histoplasmosis, blastomycosis, or coccidioidomycosis were reported; **[i]** All cases of neutropenia except 1 patient with neutrophil count decreased in BE OPTIMAL Year 1 and Year 2 and BE COMPLETE Year 1, and 5 patients neutrophil count decreased in BE COMPLETE Year 2; no cases of neutrophil count decreased in Year 3 in either study; **[j]** One case of dermatitis classed as serious due to the patient requiring hospitalization; **[k]** Identified using the high-level terms “administration site reactions NEC” and “injection site reactions”; **[l]** Elevated liver enzymes included the following preferred terms reported as adverse events: increased/abnormal levels of ALT, AST, blood bilirubin, gamma-glutamyltransferase, hepatic enzyme, liver function test, total bile acids, or transaminases; **[m]** Malignancies reported in an additional 3 patients in Year 1 and 3 patients in Year 3 in BE OPTIMAL and an additional 1 patient in Year 2 in BE COMPLETE when including nonmelanoma skin cancer; **[n]** No cases of completed suicide; **[o]** Cases deemed definite or probable IBD by the investigator; **[p]** One patient had prior history of IBD; **[q]** Patient did not have prior history of IBD; **[r]** Uveitis TEAEs identified using the preferred terms “autoimmune uveitis”, “iridocyclitis”, “iritis”, and “uveitis”. **ALT:** alanine aminotransferase; **AST:** aspartate aminotransferase; **bDMARD:** biologic disease-modifying antirheumatic drug; **BKZ:** bimekizumab; **CI:** confidence interval; **EAIR:** exposure-adjusted incidence rate; **IBD:** inflammatory bowel disease; **MACE:** major adverse cardiac event; **NEC:** not elsewhere classified; **PBO:** placebo; **PY:** patient-years; **Q4W:** every four weeks; **TEAE:** treatment‑emergent adverse event; **TNFi-IR:** prior inadequate response or intolerance to tumour necrosis factor inhibitors; **ULN:** upper limit of normal

.

**Supplementary Table S3.** Fungal events to 3 years

|  | **BE OPTIMAL**  (bDMARD-naïve) | | **BE COMPLETE**  (TNFi-IR) |
| --- | --- | --- | --- |
| **n^a^ (%)**  **EAIR/100 PY (95% CI)** | **BKZ 160 mg Q4W Total^b^  n=702**  **(1,794.3 PY)** | **BKZ 160 mg Q4W  All Patients^c^**  **N=823**  **(2,022.1 PY)** | **BKZ 160 mg Q4W  Total^b^**  **N=388  (985.3 PY)** |
| Serious fungal infections | 1 (0.1)  0.1 (0.0–0.3)^d^ | 1 (0.1)  0.1 (0.0–0.3)^d^ | 0 |
| Treatment discontinuations due to fungal infections^e^ | 9 (1.3)  0.5 (0.2–1.0) | 9 (1.1)  0.5 (0.2–0.9) | 4 (1.0)  0.4 (0.1–1.0) |
| Treatment discontinuations due to *Candida* infection | 7 (1.0)  0.4 (0.2–0.8) | 7 (0.9)  0.4 (0.1–0.7) | 4 (1.0)  0.4 (0.1–1.0) |
| Fungal infections | 144 (20.5)  9.2 (7.8–10.9) | 163 (19.8)  9.2 (7.9–10.8) | 52 (13.4)  5.8 (4.3–7.6) |
| *Candida* infections | 97 (13.8)  5.9 (4.8–7.2) | 106 (12.9)  5.7 (4.7–6.9) | 37 (9.5)  4.0 (2.8–5.5) |
| Oral candidiasis | 73 (10.4)  4.3 (3.4–5.5) | 82 (10.0)  4.3 (3.4–5.4) | 34 (8.8)  3.6 (2.5–5.1) |
| Vulvovaginal candidiasis | 13 (1.9)  0.7 (0.4–1.3) | 13 (1.6)  0.7 (0.4–1.1) | 1 (0.3)  0.1 (0.0–0.6) |
| Skin candida | 8 (1.1)  0.5 (0.2–0.9) | 8 (1.0)  0.4 (0.2–0.8) | 0 |
| Oesophageal candidiasis | 7 (1.0)  0.4 (0.2–0.8) | 7 (0.9)  0.4 (0.1–0.7) | 2 (0.5)  0.2 (0.0–0.7) |
| Oropharyngeal candidiasis | 5 (0.7)  0.3 (0.1–0.7) | 5 (0.6)  0.3 (0.1–0.6) | 0 |
| *Candida* infection | 1 (0.1)  0.1 (0.0–0.3) | 1 (0.1)  0.1 (0.0–0.3) | 1 (0.3)  0.1 (0.0–0.6) |
| Otitis externa candida | 1 (0.1)  0.1 (0.0–0.3) | 1 (0.1)  0.1 (0.0–0.3) | 0 |
| Fungal infections NEC | 61 (8.7)  3.6 (2.8–4.6) | 71 (8.6)  3.7 (2.9–4.7) | 18 (4.6)  1.9 (1.1–3.0) |
| Oral fungal infection | 21 (3.0)  1.2 (0.7–1.8) | 26 (3.2)  1.3 (0.9–1.9) | 2 (0.5)  0.2 (0.0–0.7) |
| Fungal skin infection | 16 (2.3)  0.9 (0.5–1.5) | 20 (2.4)  1.0 (0.6–1.6) | 6 (1.5)  0.6 (0.2–1.3) |
| Vulvovaginal mycotic infection | 11 (1.6)  0.6 (0.3–1.1) | 12 (1.5)  0.6 (0.3–1.1) | 6 (1.5)  0.6 (0.2–1.3) |
| Tongue fungal infection | 5 (0.7)  0.3 (0.1–0.7) | 6 (0.7)  0.3 (0.1–0.7) | 2 (0.5)  0.2 (0.0–0.7) |
| Onychomycosis | 4 (0.6)  0.2 (0.1–0.6) | 5 (0.6)  0.3 (0.1–0.6) | 1 (0.3)  0.1 (0.0–0.6) |
| Fungal oesophagitis | 3 (0.4)  0.2 (0.0–0.5) | 3 (0.4)  0.2 (0.0–0.4) | 0 |
| Fungal infection | 1 (0.1)  0.1 (0.0–0.3) | 1 (0.1)  0.1 (0.0–0.3) | 0 |
| Fungal pharyngitis | 1 (0.1)  0.1 (0.0–0.3) | 1 (0.1)  0.1 (0.0–0.3) | 0 |
| Gastrointestinal fungal infection | 1 (0.1)  0.1 (0.0–0.3) | 1 (0.1)  0.1 (0.0–0.3) | 0 |
| Oropharyngitis fungal | 1 (0.1)  0.1 (0.0–0.3) | 1 (0.1)  0.1 (0.0–0.3) | 0 |
| Otitis media fungal | 1 (0.1)  0.1 (0.0–0.3) | 1 (0.1)  0.1 (0.0–0.3) | 0 |
| Laryngitis fungal | 1 (0.1)  0.1 (0.0–0.3) | 1 (0.1)  0.1 (0.0–0.3) | 0 |
| Upper respiratory fungal  infection | 1 (0.1)  0.1 (0.0–0.3) | 1 (0.1)  0.1 (0.0–0.3) | 0 |
| Eye infection, fungal | 0 | 0 | 1 (0.3)  0.1 (0.0–0.6) |
| *Tinea* infections | 11 (1.6)  0.6 (0.3–1.1) | 12 (1.5)  0.6 (0.3–1.1) | 6 (1.5)  0.6 (0.2–1.3) |
| *Tinea* pedis | 4 (0.6)  0.2 (0.1–0.6) | 5 (0.6)  0.3 (0.1–0.6) | 2 (0.5)  0.2 (0.0–0.7) |
| *Tinea* versicolour | 4 (0.6)  0.2 (0.1–0.6) | 4 (0.5)  0.2 (0.1–0.5) | 1 (0.3)  0.1 (0.0–0.6) |
| Body *Tinea* | 1 (0.1)  0.1 (0.0–0.3) | 1 (0.1)  0.1 (0.0–0.3) | 1 (0.3)  0.1 (0.0–0.6) |
| *Tinea* infection | 1 (0.1)  0.1 (0.0–0.3) | 1 (0.1)  0.1 (0.0–0.3) | 1 (0.3)  0.1 (0.0–0.6) |
| *Tinea* cruris | 1 (0.1)  0.1 (0.0–0.3) | 1 (0.1)  0.1 (0.0–0.3) | 1 (0.3)  0.1 (0.0–0.6) |
| *Tinea* capitis | 0 | 0 | 1 (0.3)  0.1 (0.0–0.6) |

Safety set. **[a]** ‘n’ denotes the number of patients reporting at least one of the respective TEAE; **[b]** Bimekizumab Total group includes bimekizumab‑randomized patients and placebo-randomized patients that switched to bimekizumab at Week 16; includes events after switch only; **[c]** All Patients group includes all patients that had received at least 1 dose of bimekizumab, including patients that switched to bimekizumab from the reference arm (adalimumab) at Week 52; includes events after switch only; **[d]** One case of oropharyngeal candidiasis; **[e]** There were no documented cases of recurrent fungal infections leading to study treatment discontinuation. **bDMARD:** biologic disease-modifying antirheumatic drug; **BKZ:** bimekizumab; **CI:** confidence interval; **EAIR:** exposure-adjusted incidence rate; **NEC:** not elsewhere classified; **PBO:** placebo; **PY:** patient‑years; **Q4W:** every four weeks; **TEAE:** treatment-emergent adverse event; **TNFi-IR:** prior inadequate response or intolerance to tumour necrosis factor inhibitors.

**Supplementary Figure S3.** Incidence rates of *Candida* infections by study year


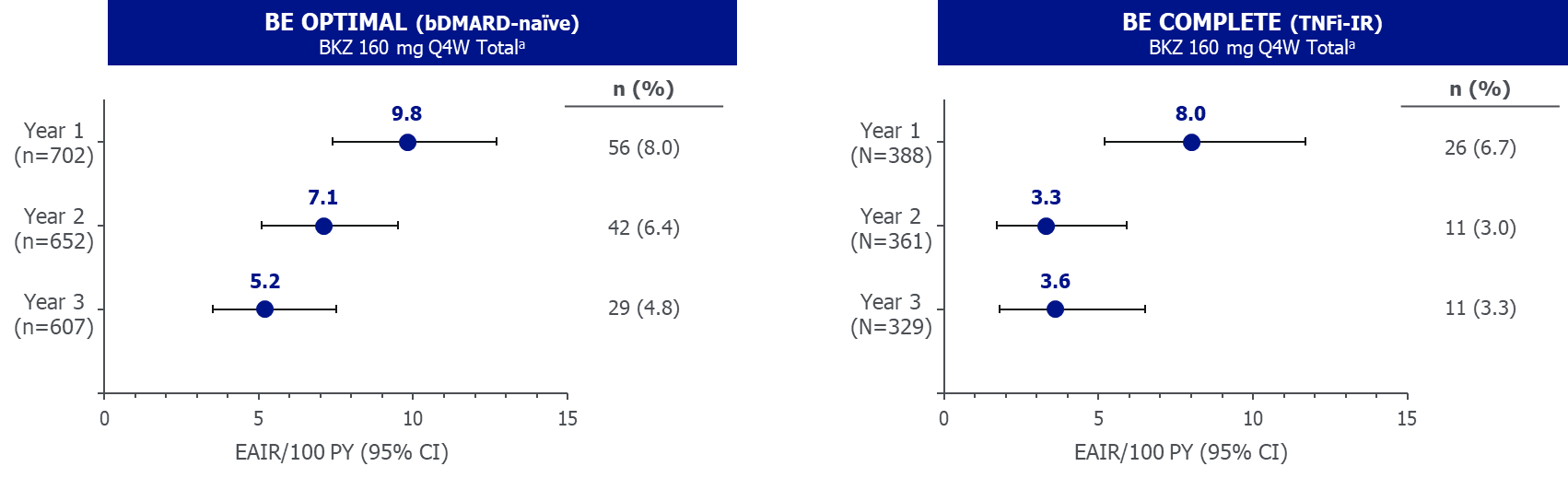


Safety set. Data reported at Year 1 (Weeks 0–52), Year 2 (Weeks >52–104) and Year 3 (Weeks >104–156). Error bars represent 95% CIs. **[a]** Bimekizumab Total group includes bimekizumab‑randomized patients and placebo-randomized patients that switched to bimekizumab at Week 16; includes events after switch only. **bDMARD:** biologic disease‑modifying antirheumatic drug; **BKZ:** bimekizumab; **CI:** confidence interval; **EAIR:** exposure-adjusted incidence rate; **PY:** patient-years; **Q4W:** every four weeks; **TNFi‑IR:** prior inadequate response or intolerance to tumour necrosis factor inhibitors.

**Supplementary Table S4.** Malignancies to 3 years

|  | **BE OPTIMAL**  (bDMARD-naïve) | | **BE COMPLETE**  (TNFi-IR) |
| --- | --- | --- | --- |
| **n^a^ (%)**  **EAIR/100 PY (95% CI)** | **BKZ 160 mg Q4W  Total^b^  n=702**  **(1,794.3 PY)** | **BKZ 160 mg Q4W  All Patients^c^**  **N=823**  **(2,022.1 PY)** | **BKZ 160 mg Q4W  Total^b^**  **N=388  (985.3 PY)** |
| Any malignancies | 14 (2.0)  0.8 (0.4–1.3) | 14 (1.7)  0.7 (0.4–1.2) | 11 (2.8)  1.1 (0.6–2.0) |
| Malignancies excluding nonmelanoma skin cancer | 9 (1.3)  0.5 (0.2–1.0) | 9 (1.1)  0.5 (0.2–0.9) | 10 (2.6)  1.0 (0.5–1.9) |
| Breast cancer stage 1 | 0 | 0 | 0 |
| Breast cancer | 1 (0.1)  0.1 (0.0–0.3) | 1 (0.1)  0.1 (0.0–0.3) | 2 (0.5)  0.2 (0.0–0.7) |
| Colon cancer | 1 (0.1)  0.1 (0.0–0.3) | 1 (0.1)  0.1 (0.0–0.3) | 0 |
| Chronic lymphocytic leukaemia | 0 | 0 | 1 (0.3)  0.1 (0.0–0.6) |
| Chronic lymphocytic leukaemia stage 0 | 1 (0.1)  0.1 (0.0–0.3) | 1 (0.1)  0.1 (0.0–0.3) | 0 |
| Papillary thyroid cancer | 1 (0.1)  0.1 (0.0–0.3) | 1 (0.1)  0.1 (0.0–0.3) | 0 |
| Lung adenocarcinoma | 1 (0.1)  0.1 (0.0–0.3) | 1 (0.1)  0.1 (0.0–0.3) | 0 |
| Ovarian cancer | 1 (0.1)  0.1 (0.0–0.3) | 1 (0.1)  0.1 (0.0–0.3) | 1 (0.3)  0.1 (0.0–0.6) |
| Renal cancer stage 1 | 1 (0.1)  0.1 (0.0–0.3) | 1 (0.1)  0.1 (0.0–0.3) | 0 |
| Bone cancer metastatic | 0 | 0 | 1 (0.3)  0.1 (0.0–0.6) |
| Endometrial cancer stage 1 | 0 | 0 | 1 (0.3)  0.1 (0.0–0.6) |
| Gastric cancer | 0 | 0 | 1 (0.3)  0.1 (0.0–0.6) |
| Gastric cancer recurrent | 0 | 0 | 1 (0.3)  0.1 (0.0–0.6) |
| Plasma cell myeloma | 0 | 0 | 1 (0.3)  0.1 (0.0–0.6) |
| Prostate cancer | 0 | 0 | 2 (0.5)  0.2 (0.0–0.7) |
| Skin cancer | 1 (0.1)  0.1 (0.0–0.3) | 1 (0.1)  0.1 (0.0–0.3) | 0 |
| Uterine cancer | 1 (0.1)  0.1 (0.0–0.3) | 1 (0.1)  0.1 (0.0–0.3) | 0 |

Safety set. **[a]** ‘n’ denotes the number of patients reporting at least one of the respective TEAE; **[b]** Bimekizumab Total group includes bimekizumab‑randomized patients and placebo-randomized patients that switched to bimekizumab at Week 16; includes events after switch only; **[c]** All Patients group includes all patients that had received at least 1 dose of bimekizumab, including patients that switched to bimekizumab from the reference arm (adalimumab) at Week 52; includes events after switch only. **bDMARD:** biologic disease‑modifying antirheumatic drug; **BKZ:** bimekizumab; **CI:** confidence interval; **EAIR:** exposure-adjusted incidence rate; **PBO:** placebo; **PY:** patient-years; **Q4W:** every four weeks; **TEAE:** treatment-emergent adverse event; **TNFi-IR:** prior inadequate response or intolerance to tumour necrosis factor inhibitors.

**Supplementary Table S5.** Adjudicated major adverse cardiovascular events to 3 years

|  | **BE OPTIMAL**  (bDMARD-naïve) | | **BE COMPLETE**  (TNFi-IR) |
| --- | --- | --- | --- |
| **n^a^ (%)**  **EAIR/100 PY (95% CI)** | **BKZ 160 mg Q4W  Total^b^  n=702**  **(1,794.3 PY)** | **BKZ 160 mg Q4W  All Patients^c^**  **N=823**  **(2,022.1 PY)** | **BKZ 160 mg Q4W  Total^b^**  **N=388  (985.3 PY)** |
| Any adjudicated MACE | 7 (1.0)  0.4 (0.2–0.8) | 9 (1.1)  0.5 (0.2–0.9) | 2 (0.5)  0.2 (0.0–0.7) |
| Acute myocardial infarction | 1 (0.1)  0.1 (0.0–0.3) | 2 (0.2)  0.1 (0.0–0.4) | 0 |
| Cardiac arrest | 1 (0.1)  0.1 (0.0–0.3) | 1 (0.1)  0.1 (0.0–0.3) | 0 |
| Cerebral infarction | 1 (0.1)  0.1 (0.0–0.3) | 1 (0.1)  0.1 (0.0–0.3) | 0 |
| Thrombotic cerebral infarction | 1 (0.1)  0.1 (0.0–0.3) | 1 (0.1)  0.1 (0.0–0.3) | 0 |
| Cerebral haemorrhage | 0 | 0 | 1 (0.3)  0.1 (0.0–0.6) |
| Cerebrovascular accident | 1 (0.1)  0.1 (0.0–0.3) | 1 (0.1)  0.1 (0.0–0.3) | 0 |
| Ischaemic stroke | 2 (0.3)  0.1 (0.0–0.4) | 3 (0.4)  0.2 (0.0–0.4) | 0 |
| Monoparesis | 1 (0.1)  0.1 (0.0–0.3) | 1 (0.1)  0.1 (0.0–0.3) | 0 |
| Myocardial infarction | 1 (0.1)  0.1 (0.0–0.3) | 1 (0.1)  0.1 (0.0–0.3) | 0 |
| Sudden death | 0 | 0 | 1 (0.3)  0.1 (0.0–0.6) |

Safety set. **[a]** ‘n’ denotes the number of patients reporting at least one of the respective TEAE; **[b]** Bimekizumab Total group includes bimekizumab‑randomized patients and placebo-randomized patients that switched to bimekizumab at Week 16; includes events after switch only; **[c]** All Patients group includes all patients that had received at least 1 dose of bimekizumab, including patients that switched to bimekizumab from the reference arm (adalimumab) at Week 52; includes events after switch only. **bDMARD:** biologic disease‑modifying antirheumatic drug; **BKZ:** bimekizumab; **CI:** confidence interval; **EAIR:** exposure-adjusted incidence rate; **MACE:** major adverse cardiac event; **PBO:** placebo; **PY:** patient‑years; **Q4W:** every four weeks; **TEAE:** treatment‑emergent adverse event; **TNFi-IR:** prior inadequate response or intolerance to tumour necrosis factor inhibitors.

**Supplementary Table S6.** Adjudicated suicidal ideation and behaviour events to 3 years

|  | **BE OPTIMAL**  (bDMARD-naïve) | | **BE COMPLETE**  (TNFi-IR) |
| --- | --- | --- | --- |
| **n^a^ (%)**  **EAIR/100 PY (95% CI)** | **BKZ 160 mg Q4W  Total^b^  n=702**  **(1,794.3 PY)** | **BKZ 160 mg Q4W  All Patients^c^**  **N=823**  **(2,022.1 PY)** | **BKZ 160 mg Q4W  Total^b^**  **N=388  (985.3 PY)** |
| Any adjudicated suicidal ideation and behaviour | 2 (0.3)  0.1 (0.0–0.4) | 2 (0.2)  0.1 (0.0–0.4) | 0 |
| Psychiatric evaluation abnormal | 1 (0.1)  0.1 (0.0–0.3) | 1 (0.1)  0.1 (0.0–0.3) | 0 |
| Suicidal behaviour^d^ | 1 (0.1)  0.1 (0.0–0.3) | 1 (0.1)  0.1 (0.0–0.3) | 0 |

Safety set. **[a]** ‘n’ denotes the number of patients reporting at least one of the respective TEAE; **[b]** Bimekizumab Total group includes bimekizumab‑randomized patients and placebo-randomized patients that switched to bimekizumab at Week 16; includes events after switch only; **[c]** All Patients group includes all patients that had received at least 1 dose of bimekizumab, including patients that switched to bimekizumab from the reference arm (adalimumab) at Week 52; includes events after switch only; **[d]** No cases of completed suicide. **bDMARD:** biologic disease-modifying antirheumatic drug; **BKZ:** bimekizumab; **CI:** confidence interval; **EAIR:** exposure‑adjusted incidence rate; **PBO:** placebo; **PY:** patient-years; **Q4W:** every four weeks; **TEAE:** treatment-emergent adverse event; **TNFi-IR:** prior inadequate response or intolerance to tumour necrosis factor inhibitors.

**Supplementary Figure S4.** ACR20 and PASI75 to Year 3 (mNRI, NRI, OC)


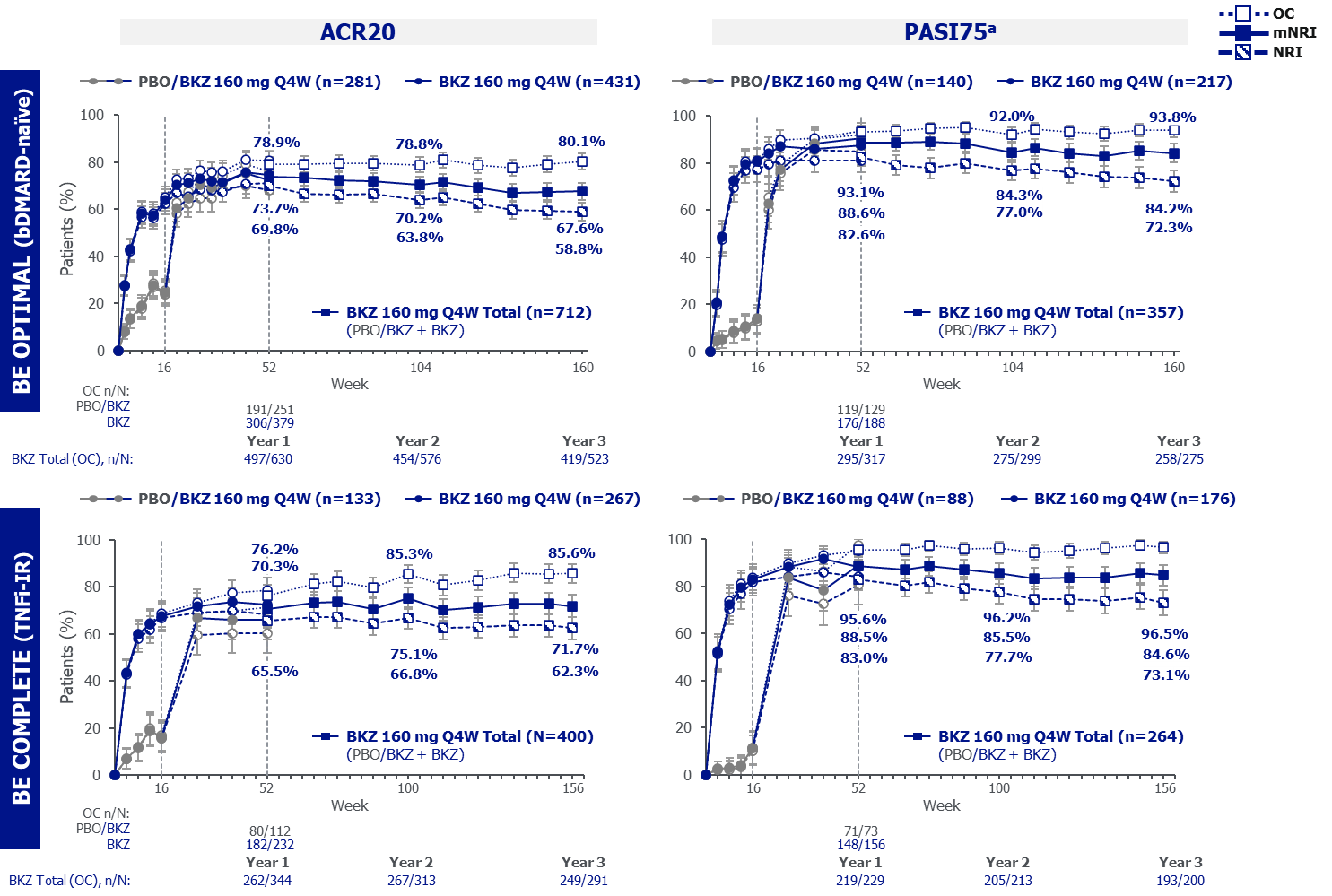


Randomized set. Bimekizumab Total group includes bimekizumab-randomized patients and placebo‑randomized patients that switched to bimekizumab at Week 16. Data reported to Year 3 (Week 160 in BE OPTIMAL and Week 156 in BE COMPLETE). mNRI considered all visits following discontinuation due to adverse event or lack of efficacy as non‑response; all other missing data were imputed with multiple imputation and the response derived from the imputed values. Error bars represent 95% CIs. **[a]** In patients with BSA ≥3% at baseline. **ACR20:** ≥20% improvement from baseline in American College of Rheumatology response criteria; **bDMARD:** biologic disease-modifying antirheumatic drug; **BKZ:** bimekizumab; **BSA:** body surface area; **CI:** confidence interval; **mNRI:** modified non-responder imputation; **NRI:** non-responder imputation; **OC:** observed case; **PASI75:** ≥75% improvement from baseline in Psoriasis Area and Severity Index; **PBO:** placebo; **Q4W:** every four weeks; **TNFi-IR:** prior inadequate response or intolerance to tumour necrosis factor inhibitors.

**Supplementary Figure S5.** LEI and LDI resolution to Year 3 (mNRI, NRI, OC)


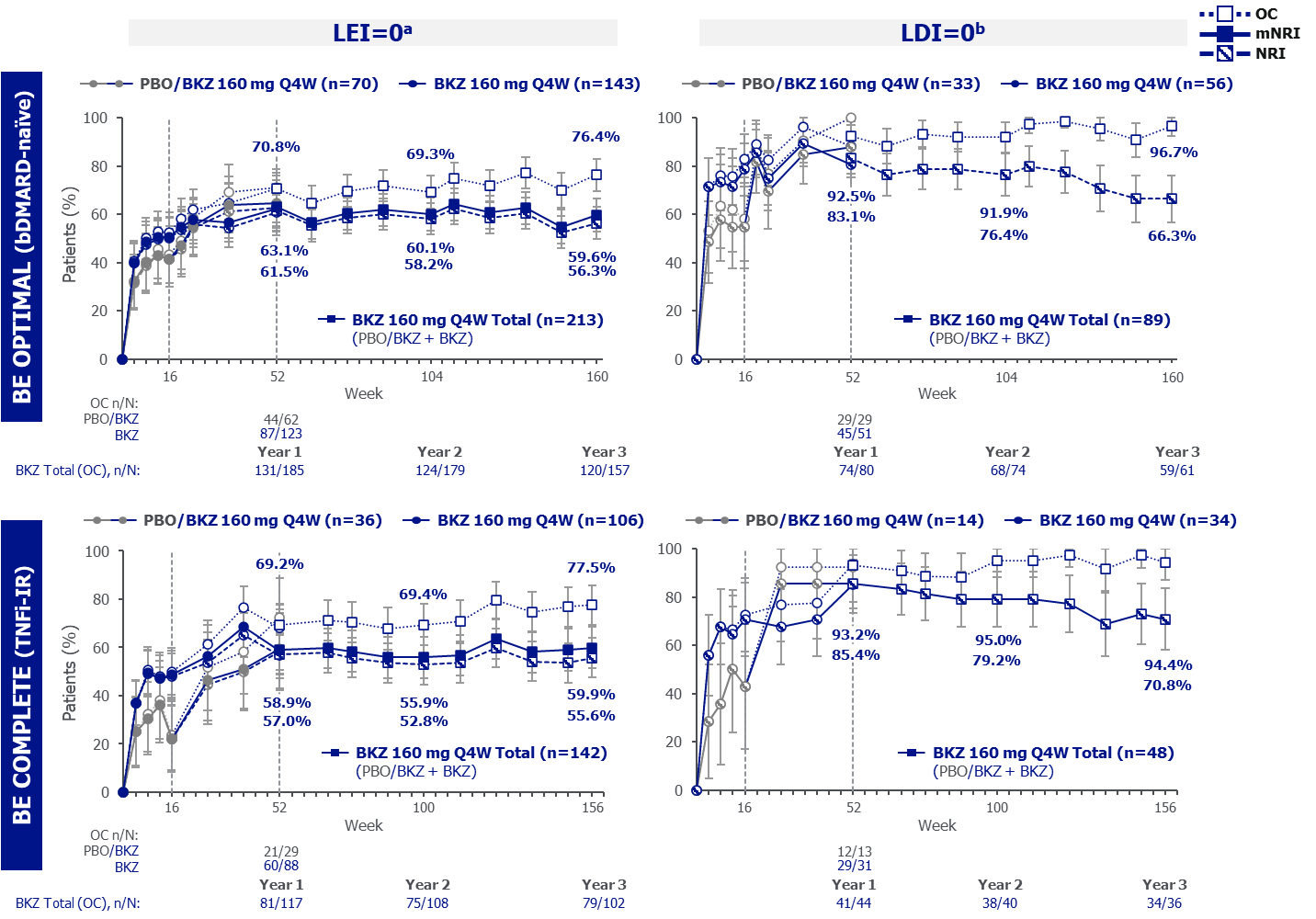


Randomized set. Bimekizumab Total group includes bimekizumab‑randomized patients and placebo‑randomized patients that switched to bimekizumab at Week 16. Data reported to Year 3 (Week 160 in BE OPTIMAL and Week 156 in BE COMPLETE). mNRI considered all visits following discontinuation due to AEs or lack of efficacy as non-response; all other missing data were imputed with multiple imputation and the response derived from the imputed values. Error bars represent 95% CIs. **[a]** In patients with enthesitis (LEI >0) at baseline; **[b]** In patients with dactylitis (LDI >0) at baseline; missing data imputed using NRI as MI did not converge and mNRI was not available. **AE:** adverse event; **bDMARD:** biologic disease-modifying antirheumatic drug; **BKZ:** bimekizumab; **CI:** confidence interval; **LDI:** Leeds Dactylitis Index; **LEI:** Leeds Enthesitis Index; **mNRI:** modified non-responder imputation; **NRI:** non-responder imputation; **OC:** observed case; **PBO:** placebo; **Q4W:** every four weeks; **TNFi‑IR:** prior inadequate response or intolerance to tumour necrosis factor inhibitors.

**Supplementary Figure S6.** DAPSA (A) and PASDAS (B) disease states to Year 3 (MI)

1. DAPSA^a^


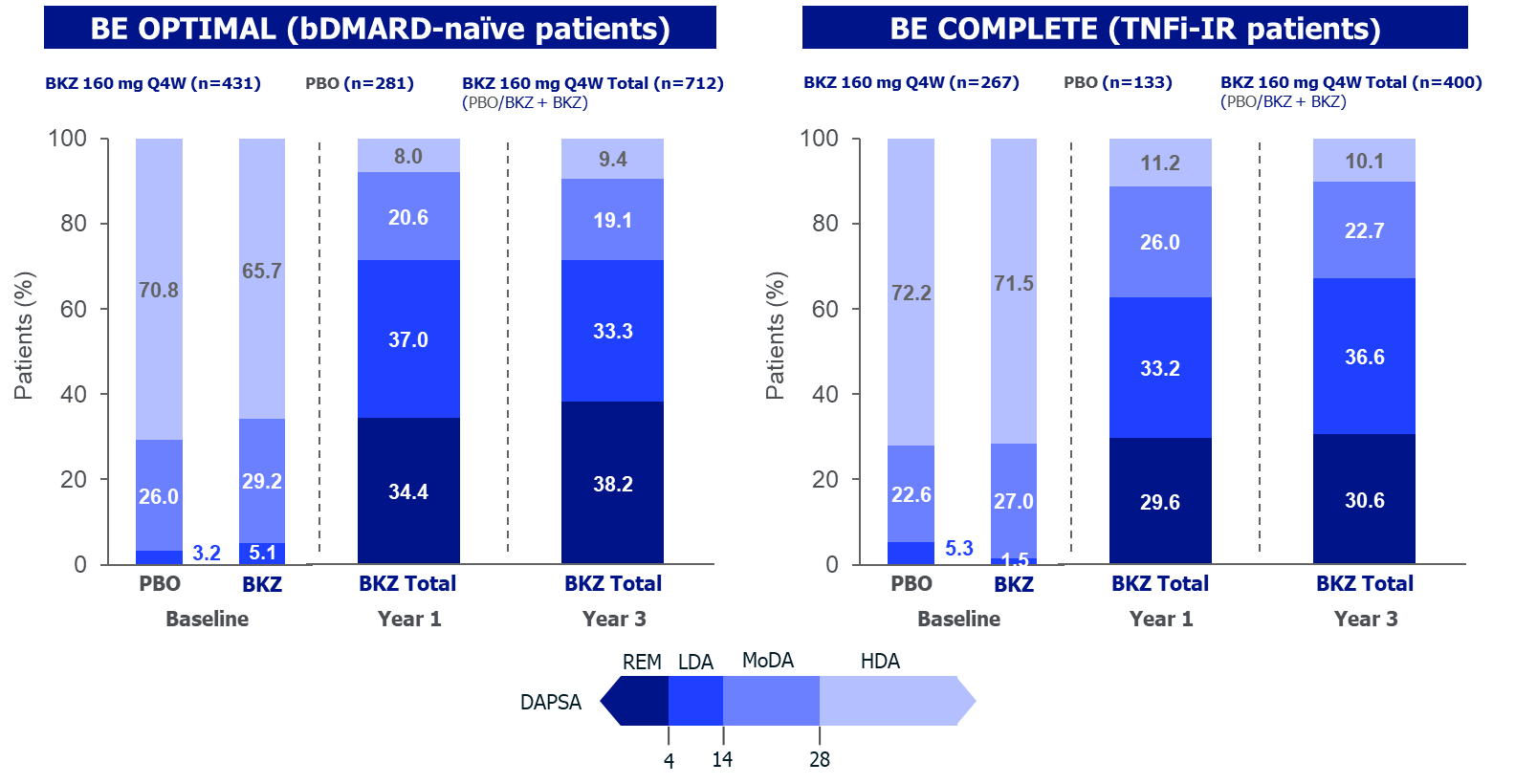


1. PASDAS^b^


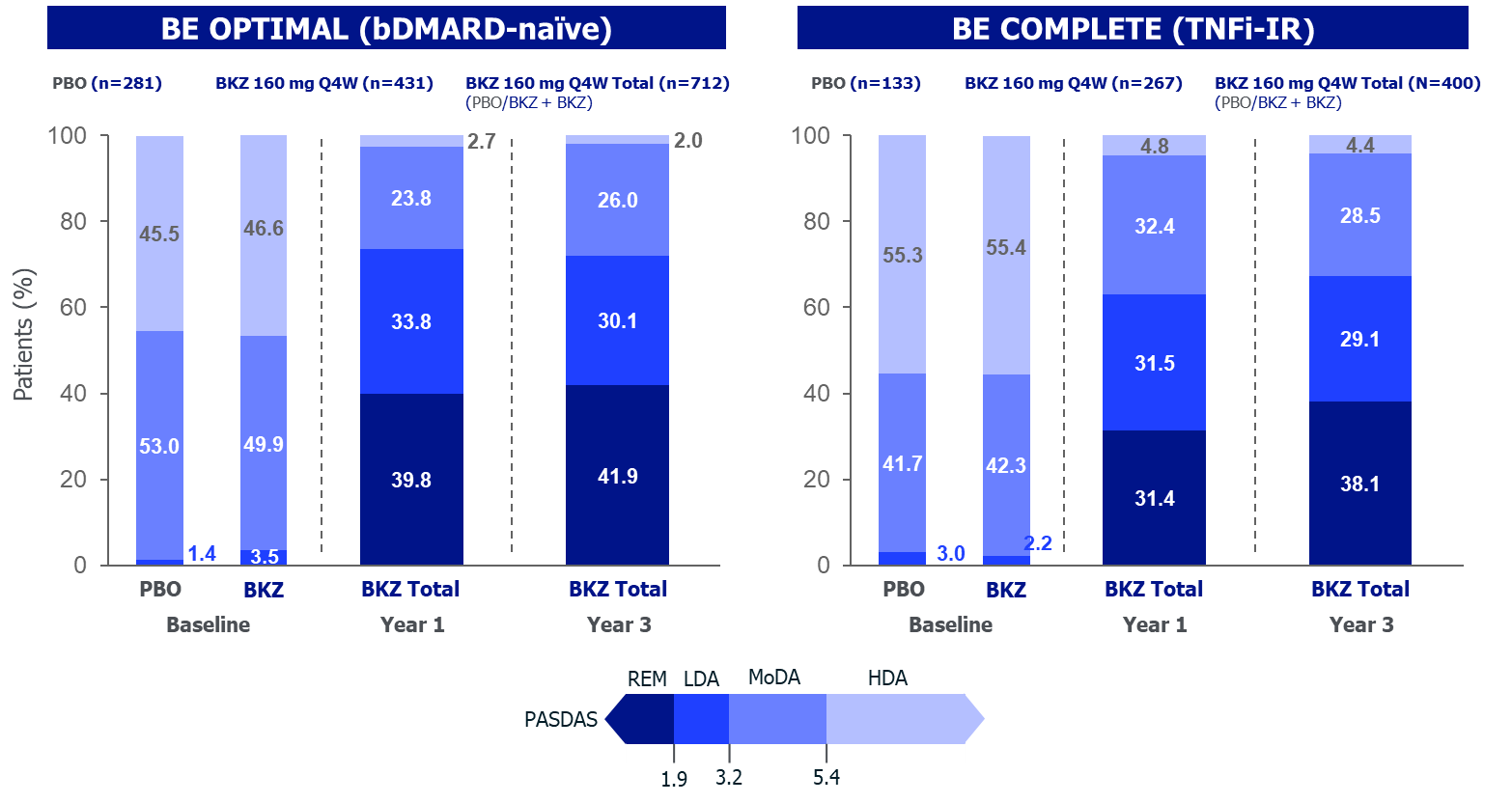


Randomized set. Bimekizumab Total group includes bimekizumab‑randomized patients and placebo‑randomized patients that switched to bimekizumab at Week 16. **[a]** Data reported at baseline, Year 1 (Week 52) and Year 3 (Week 160 in BE OPTIMAL and Week 156 in BE COMPLETE); **[b]** Data reported at baseline, Year 1 (Week 52 in BE OPTIMAL and Week 40 in BE COMPLETE) and Year 3 (Week 148 in BE OPTIMAL and Week 156 in BE COMPLETE). **bDMARD:**biologic disease-modifying antirheumatic drug; **BKZ:** bimekizumab; **DAPSA:** Disease Activity Index for Psoriatic Arthritis; **HDA:** high disease activity; **LDA:** low disease activity; **MI:** multiple imputation; **MoDA:** moderate disease activity; **PASDAS;** Psoriatic Arthritis Disease Activity Score; **PBO:** placebo; **Q4W:** every four weeks; **REM:** remission; **TNFi-IR:** prior inadequate response or intolerance to tumour necrosis factor inhibitors.

**Supplementary Figure S7.** Joint outcomes to Year 3 (NRI)


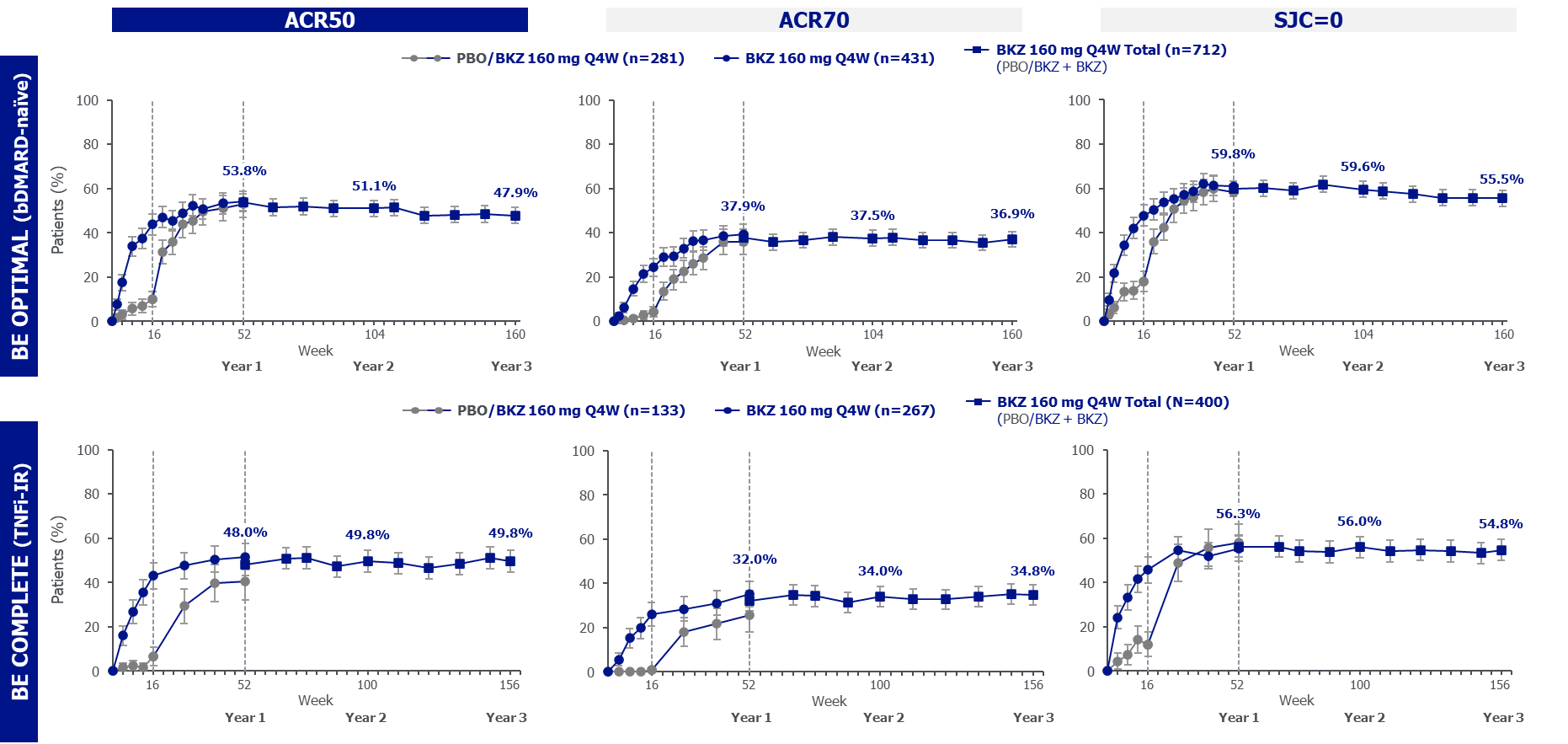


Randomized set. Bimekizumab Total group includes bimekizumab‑randomized patients and placebo-randomized patients that switched to bimekizumab at Week 16. Data reported to 3 years (Week 160 in BE OPTIMAL and Week 156 in BE COMPLETE). Error bars represent 95% CIs. **ACR50/70:** ≥50/70% improvement from baseline in American College of Rheumatology response criteria; **bDMARD:** biologic disease-modifying antirheumatic drug; **BKZ:** bimekizumab; **CI:** confidence interval; **NRI:** non-responder imputation; **PBO:** placebo; **Q4W:** every four weeks; **SJC:** swollen joint count; **TNFi-IR:** prior inadequate response or intolerance to tumour necrosis factor inhibitors.

**Supplementary Figure S8.** Skin and nail outcomes to Year 3 (NRI)


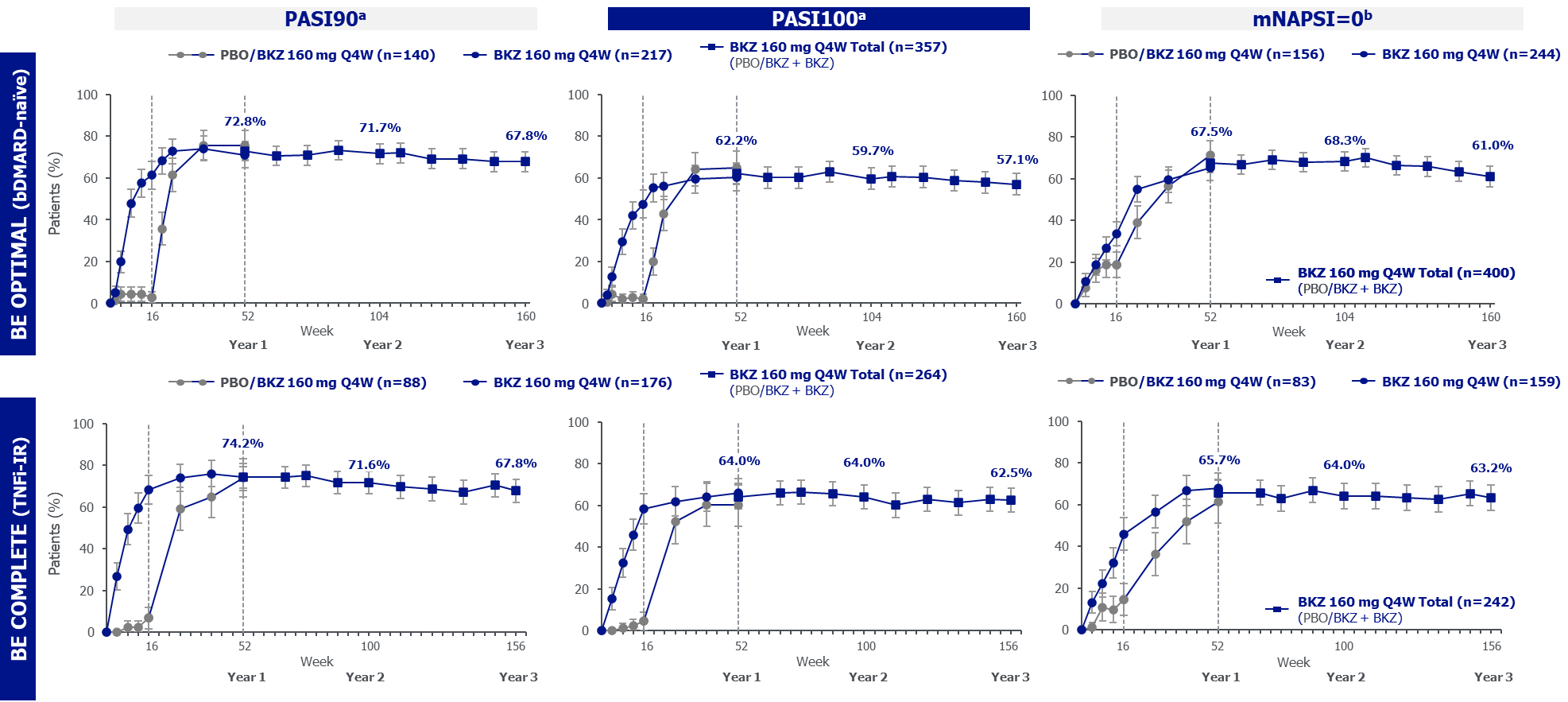


Randomized set. Bimekizumab Total group includes bimekizumab‑randomized patients and placebo-randomized patients that switched to bimekizumab at Week 16. Data reported to 3 years (Week 160 in BE OPTIMAL and Week 156 in BE COMPLETE). Error bars represent 95% CIs. **[a]** In patients with BSA ≥3% at baseline; **[b]** In patients with nail psoriasis (mNAPSI >0) at baseline. **bDMARD:** biologic disease-modifying antirheumatic drug; **BKZ:** bimekizumab; **BSA:** body surface area; **CI:** confidence interval; **mNAPSI:** modified Nail Psoriasis Severity Index; **NRI:** non-responder imputation; **PASI90/100:** ≥90/100% improvement from baseline in Psoriasis Area and Severity Index; **PBO:** placebo; **Q4W**: every four weeks; **TNFi-IR:** prior inadequate response or intolerance to tumour necrosis factor inhibitors.

**Supplementary Figure S9.** Composite outcomes to Year 3 (NRI)


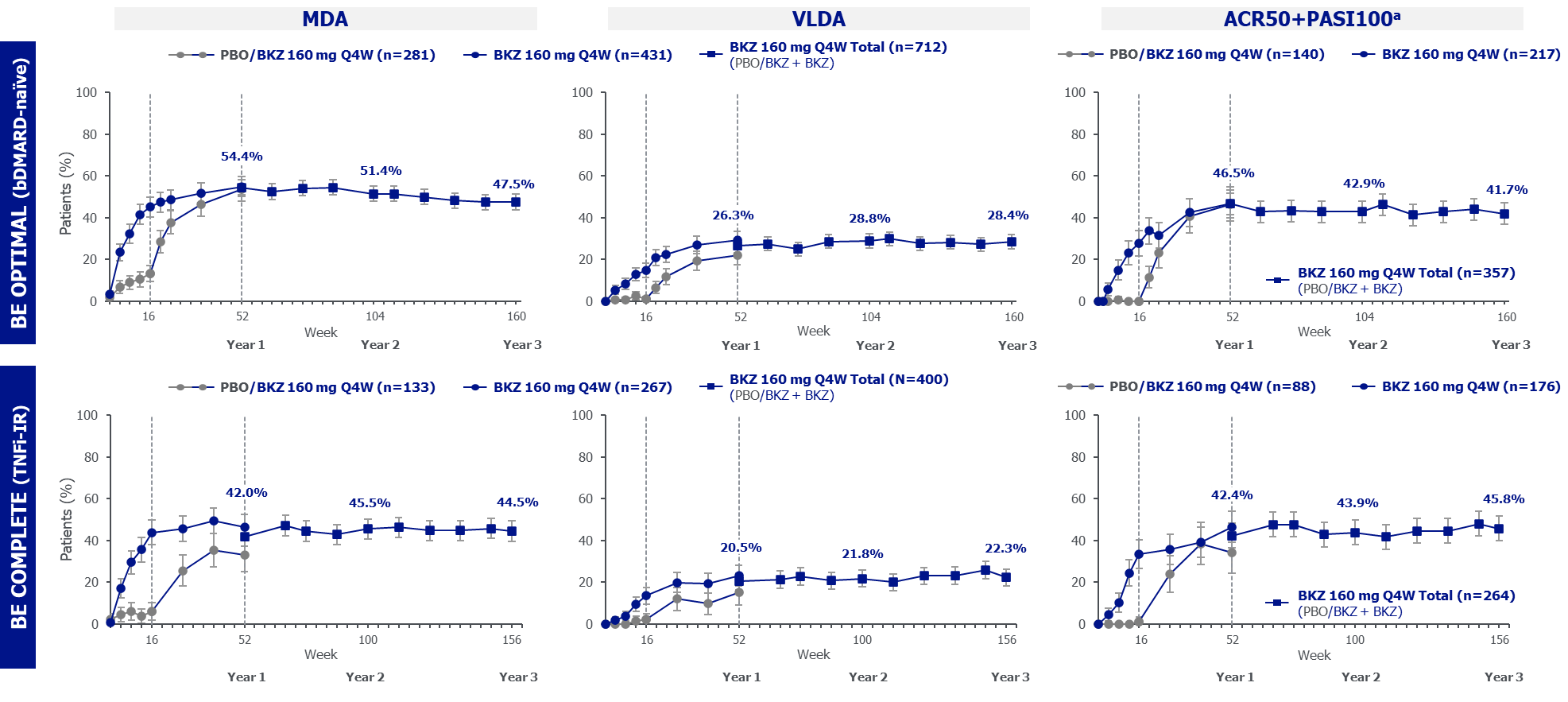


Randomized set. Bimekizumab Total group includes bimekizumab‑randomized patients and placebo-randomized patients that switched to bimekizumab at Week 16. Data reported to 3 years (Week 160 in BE OPTIMAL and Week 156 in BE COMPLETE). Error bars represent 95% CIs. **[a]** In patients with BSA ≥3% at baseline. **ACR50+PASI100:** ≥50% improvement from baseline in American College of Rheumatology response criteria + 100% improvement from baseline in Psoriasis Area and Severity Index; **bDMARD:** biologic disease-modifying antirheumatic drug; **BKZ:** bimekizumab; **CI:** confidence interval; **MDA:** minimal disease activity; **mNRI:** modified non‑responder imputation; **OC:** observed case; **PBO:** placebo; **Q4W:** every four weeks; **TNFi-IR:** prior inadequate response or intolerance to tumour necrosis factor inhibitors; **VLDA:** very low disease activity.

**Supplementary Figure S10.** Patient-Reported Outcomes to Year 3 (NRI)


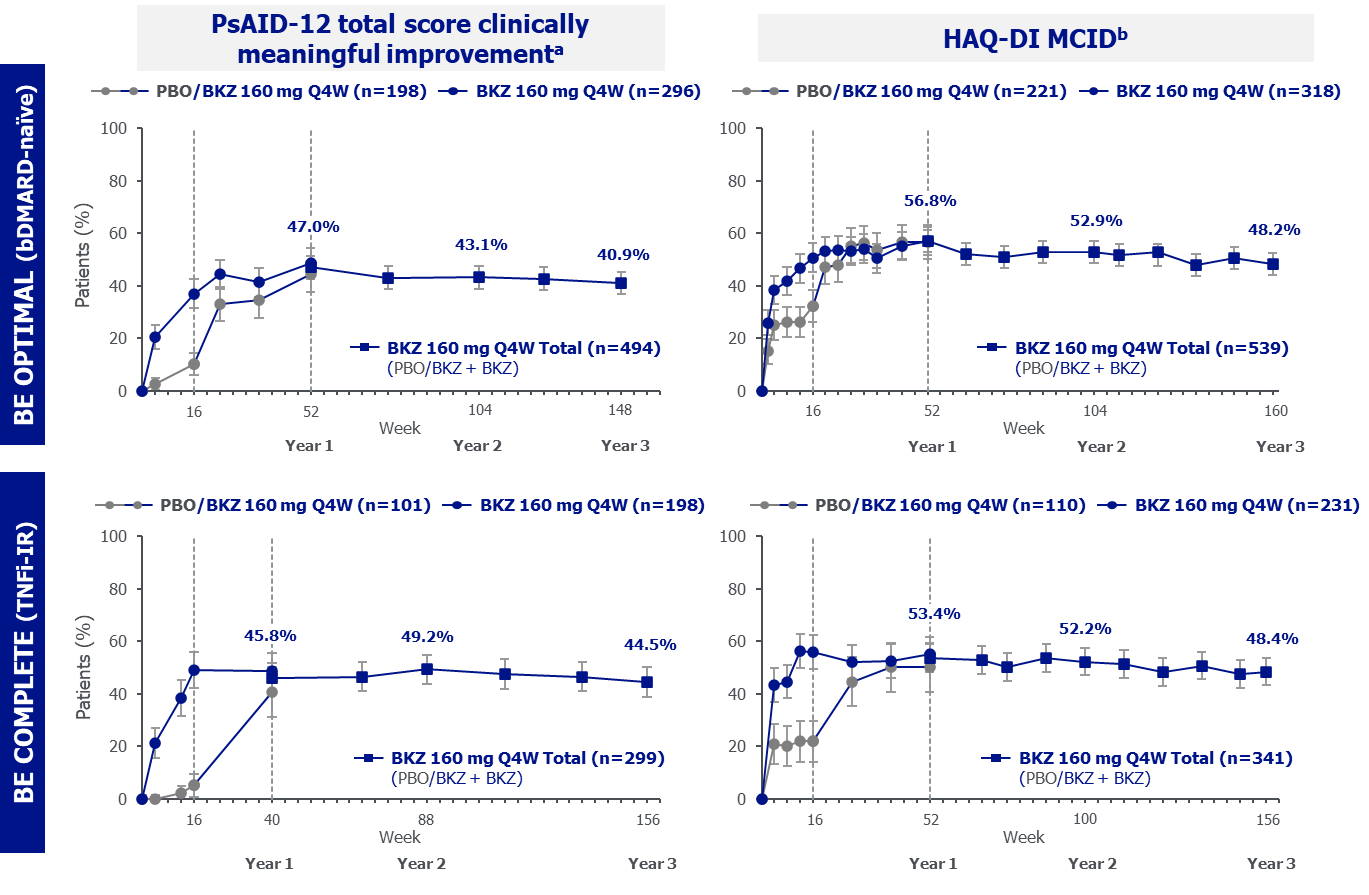


Randomized set. Bimekizumab Total group includes bimekizumab‑randomized patients and placebo-randomized patients that switched to bimekizumab at Week 16. Data reported to 3 years (Week 148 for PsAID-12 or Week 160 for HAQ-DI in BE OPTIMAL and Week 156 in BE COMPLETE). Error bars represent 95% CIs. **[a]** PsAID-12 clinically meaningful improvement defined as decrease from baseline ≥3 in patients with PsAID-12 ≥3 at baseline; Data reported at Week 40 (Year 1) and Week 88 (Year 2) for BE COMPLETE; **[b]** HAQ‑DI MCID defined as decrease from baseline ≥0.35 in patients with HAQ-DI ≥0.35 at baseline. **bDMARD:** biologic disease-modifying antirheumatic drug; **BKZ:** bimekizumab; **CI:** confidence interval; **HAQ-DI:** Health Assessment Questionnaire-Disability Index; **MCID:** minimal clinically important difference; **NRI:** non-responder imputation; **PBO:** placebo; **PsAID-12:** Psoriatic Arthritis Impact of Disease 12‑item questionnaire; **Q4W:** every four weeks; **TNFi‑IR:** prior inadequate response or intolerance to tumour necrosis factor inhibitors.

Supplementary Table S7. Key reference arm efficacy outcomes to 3 years (mNRI, NRI, OC)

|  | **BE OPTIMAL**  (bDMARD-naïve) | | | |
| --- | --- | --- | --- | --- |
|  | **ADA 40 mg Q2W  n=140** | | **ADA 40 mg Q2W → BKZ 160 mg Q4W**  **n=140** | |
|  | **Year 1** | | **Year 3** | |
|  | mNRI, % | OC, n/N (%) | mNRI, % | OC, n/N (%) |
| ACR20 responders | 74.8 | 102/123 (82.9) | 67.0 | 84/106 (79.2) |
| ACR50 responders | 51.0 | 70/123 (56.9) | 45.2 | 58/106 (54.7) |
| ACR70 responders | 38.2 | 53/123 (43.1) | 33.4 | 44/106 (41.5) |
| PASI75 responders^a^ | 67.9 | 45/60 (75.0) | 81.8 | 48/50 (96.0) |
| PASI90 responders^a^ | 61.1 | 41/60 (68.3) | 71.5 | 43/50 (86.0) |
| PASI100 responders^a^ | 48.9 | 33/60 (55.0) | 61.9 | 38/50 (76.0) |
| ACR50+PASI100 responders^a^ | 35.4 | 24/60 (40.0) | 43.0 | 28/51 (54.9) |
| MDA | 53.5 | 74/123 (60.2) | 49.0 | 64/106 (60.4) |
| VLDA | 28.5 | 39/122 (32.0) | 27.9 | 38/106 (35.8) |
| SJC=0 (of 66 joints) | 60.5 | 84/123 (68.3) | 59.1 | 77/106 (72.6) |
| TJC=0 (of 68 joints) | 38.2 | 53/123 (43.1) | 34.4 | 45/106 (42.5) |
| LEI=0^b^ | 58.3 | 21/31 (67.7) | 50.4 | 17/26 (65.4) |
| LDI=0^c^ | 72.7; NRI | 8/9 (88.9) | 72.7; NRI | 8/8 (100) |
| mNAPSI=0^d^ | 60.0 | 45/69 (65.2) | 74.4 | 55/64 (85.9) |
| DAPSA,  LDA+REM  REM | 69.8; MI  36.7; MI | 88/121 (72.7)  49/121 (40.5) | 70.5; MI  36.4; MI | 81/106 (76.4)  46/106 (43.4) |
| HAQ-DI MCID,^e^ | 60.6 | 68/101 (67.3) | 55.1 | 55/85 (64.7) |
| FACIT-Fatigue MCID^f,g^ | 48.5 | 62/116 (53.4) | 45.5 | 54/103 (52.4) |
| Pain VAS^h^ ≥50% improvement | 58.3 | 79/123 (64.2) | 52.9 | 67/106 (63.2) |
| PsAID-12 total score clinically meaningful improvement^f,i^ | 46.7 | 50/98 (51.0) | 47.2 | 48/88 (54.5) |

Randomized set. Data reported at Year 3 (Week 160 in BE OPTIMAL) in patients randomized to the reference arm. Reference arm patients switched to bimekizumab at Week 52 with no washout. mNRI and OC data reported unless otherwise stated. mNRI considered all visits following discontinuation due to AEs or lack of efficacy as non-response (n=18); all other missing data were imputed with multiple imputation and the response derived from the imputed values. In cases where MI did not converge and mNRI was not available, missing data were imputed using NRI. **[a]** In patients with psoriasis affecting ≥ 3% BSA at baseline (n=68); **[b]** In patients with enthesitis at baseline (LEI>0; n=36); **[c]** In patients with dactylitis at baseline (LDI>0; n=11); **[d]** In patients with nail psoriasis at baseline (mNAPSI>0; n=75); **[e]** HAQ-DI MCID defined as decrease from baseline ≥ 0.35 in patients with HAQ‑DI ≥ 0.35 at baseline (n=115); **[f]** Data reported to Week 148; **[g]** FACIT-Fatigue MCID defined as an increase from baseline of ≥4 in patients with FACIT-Fatigue subscale ≤48 at baseline (n=132); **[h]** Pain VAS assessed using the Patient’s Assessment of Arthritis Pain VAS which ranges from 0 to 100, 0 representing ‘no pain’ and 100 ‘most severe pain’: Pain VAS ≥50% represents a substantial improvement in patient-reported pain [28]; **[i]** PsAID-12 clinically meaningful improvement defined as decrease from baseline ≥3 in patients with PsAID-12 total score at ≥3 baseline (n=111). **ACR20/50/70:** ≥20/50/70% response in American College of Rheumatology response criteria; **ADA:** adalimumab; **AE:** adverse event; **bDMARD:** biologic disease-modifying antirheumatic drug; **BKZ:** bimekizumab; **DAPSA:** Disease Activity in Psoriatic Arthritis; **FACIT-Fatigue:** Functional Assessment of Chronic Illness Therapy-Fatigue; **HAQ-DI:** Health Assessment Questionnaire-Disability Index; **LDA:** low disease activity; **LDI:** Leeds Dactylitis Index; **LEI:** Leeds Enthesitis Index; **MCID:**minimal clinically important difference; **MDA:** minimal disease activity; **MI:** multiple imputation; **mNAPSI:** modified Nail Psoriasis Severity Index; **mNRI:** modified NRI; **NRI:** non-responder imputation; **OC:** observed case; **PASI75/90/100:** ≥75/90/100% response in Psoriasis Area and Severity Index; **PsAID-12:** Psoriatic Arthritis Impact of Disease 12-item questionnaire; **Q2W:** every two weeks; **Q4W:** every four weeks; **REM:** remission; **SJC:** swollen joint count; **TJC:** tender joint count; **VAS:** visual analogue scale; **VLDA:** very low disease activity.
