# Supplementary material for: Bimekizumab safety and efficacy in patients with psoriatic arthritis: 3-year results from two phase 3 studies
Source: Rheumatology (Oxford). 2026 Mar 16;65(5):keag118. doi: 10.1093/rheumatology/keag118 (PMC13198880; doi:10.1093/rheumatology/keag118)
Supplement: keag118_Supplementary_Data [file keag118_supplementary_data.zip › rhe-25-2713-File004.pdf]

British Society for Rheumatology

RHEUMATOLOGY

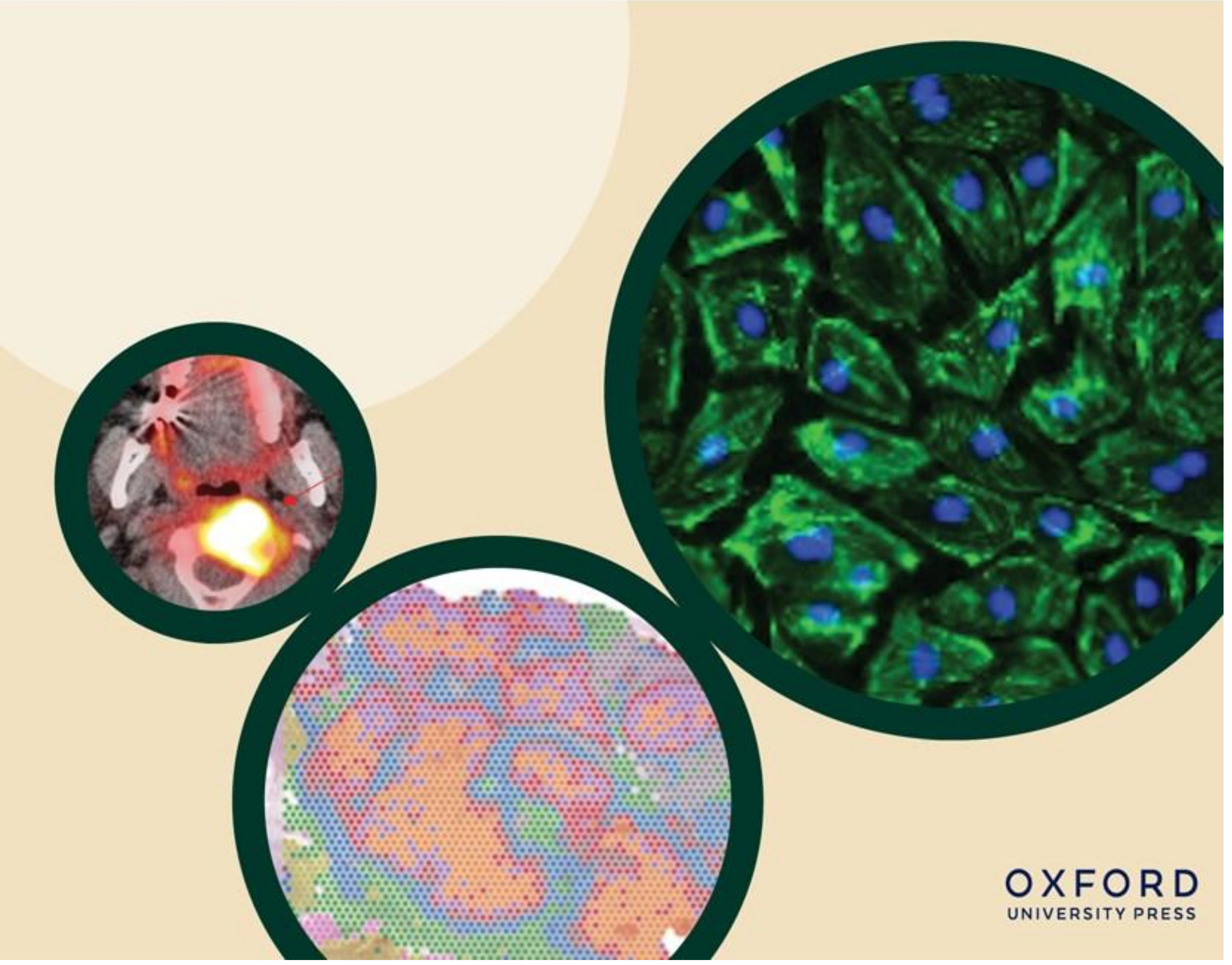

Gossec, L. et al. Bimekizumab Safety and Efficacy in Patients with Psoriatic Arthritis: 3-Year Results from Two Phase 3 Studies. *Rheumatology (Oxford)*.

SUPPLEMENTARY SLIDE DECK

These studies were funded by UCB. For a full list of declarations, including funding and author disclosure statements, and copyright information, please see the full text online.

Abbreviations

- **ACR50/70:** ≥50/70% improvement from baseline in American College of Rheumatology response criteria
- **ACR50+PASI100:** ≥50% improvement from baseline in American College of Rheumatology response criteria + 100% improvement from baseline in Psoriasis Area and Severity Index
- **AE:** adverse event
- **ALT:** alanine aminotransferase
- **AST:** aspartate aminotransferase
- **bDMARD:** biologic disease-modifying antirheumatic drug
- **BKZ:** bimekizumab
- **BSA:** body surface area
- **CI:** confidence interval
- **EAIR:** exposure-adjusted incidence rate
- **HAQ-DI:** Health Assessment Questionnaire-Disability Index
- **IBD:** inflammatory bowel disease
- **IL:** interleukin
- **MACE:** major adverse cardiac event
- **MCID:** minimal clinically important difference
- **MDA:** minimal disease activity
- **mNAPSI:** modified Nail Psoriasis Severity Index
- **mNRI:** modified non-responder imputation
- **MI:** multiple imputation
- **NEC:** not elsewhere classified
- **NRI:** non-responder imputation
- **OC:** observed case
- **PASI90/100:** ≥90/100% improvement from baseline in Psoriasis Area and Severity Index
- **PBO:** placebo
- **PsA:** psoriatic arthritis
- **PsAID-12:** PsA Impact of Disease 12-Item Questionnaire
- **PY:** patient-years
- **Q4W:** every four weeks
- **SJC:** swollen joint count
- **TEAE:** treatment-emergent adverse event
- **TNFi-IR:** prior inadequate response or intolerance to tumour necrosis factor inhibitors
- **VLDA:** very low disease activity
- **ULN:** upper limit of normal

Introduction

- Given the chronic nature of **psoriatic arthritis (PsA)**, an inflammatory disease that affects multiple domains including joints, skin, and nails,[1] it is important to report the **safety and efficacy of treatments over an extended length of time**.
- **Bimekizumab** is a monoclonal IgG1 antibody that selectively inhibits interleukin (IL)-17F in addition to IL-17A.[2]
- Bimekizumab treatment has demonstrated **tolerability and sustained clinical efficacy up to 2 years** in patients with PsA who were naïve to biologic disease-modifying antirheumatic drugs (bDMARDs) or had prior inadequate response or intolerance to tumour necrosis factor inhibitors (TNFi-IR).[3]

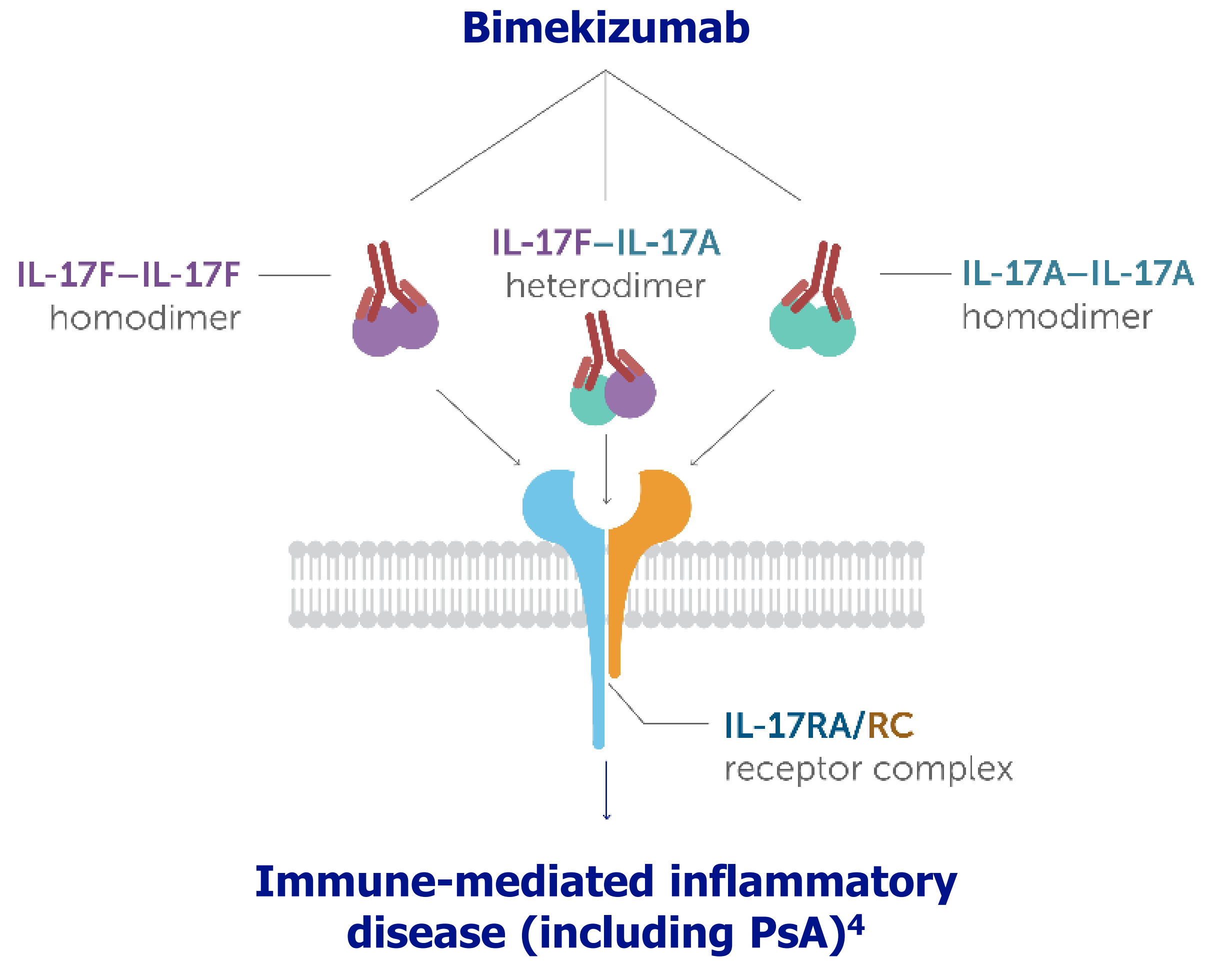

Objective

To assess the longer-term safety and clinical efficacy of bimekizumab up to 3 years in patients with active PsA who were bDMARD-naïve or TNFi-IR

1. Veale DJ. Lancet 2018;391:2273–2284; **2.** Adams R. Front Immunol 2020;11:1894; **3.** Mease PJ. Rheumatol Ther 2024;11(5):1363–82; **4.** Glatt S. Ann Rheum Dis 2018;77:523–32. **bDMARD:** biologic disease-modifying antirheumatic drug; **IL:** interleukin; **PsA:** psoriatic arthritis; **TNFi-IR:** prior inadequate response or intolerance to tumour necrosis factor inhibitors.

Methods (1/2)

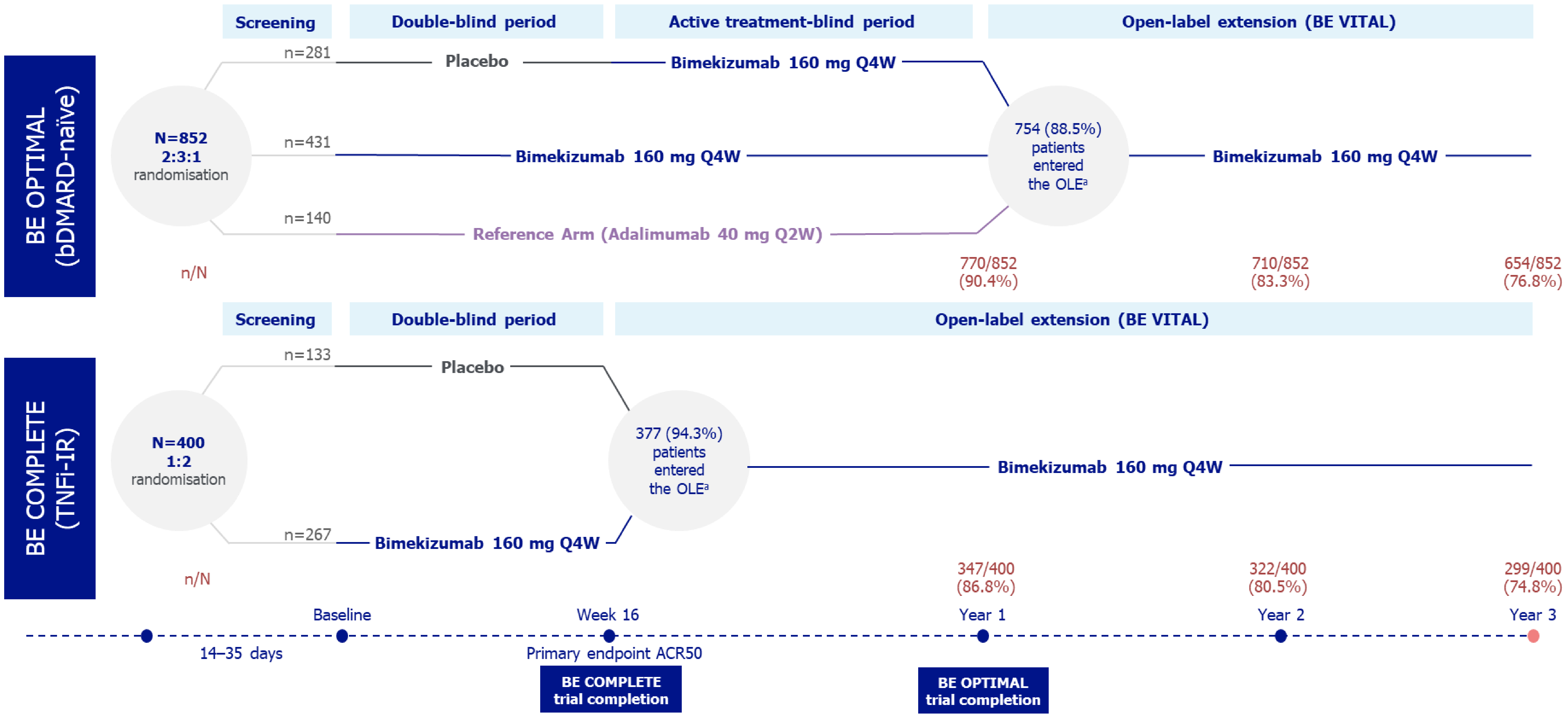

BE OPTIMAL (NCT03895203) and BE COMPLETE (NCT03896581) assessed subcutaneous bimekizumab 160 mg every 4 weeks (Q4W) in patients with PsA. BE OPTIMAL Week 52 and BE COMPLETE Week 16 completers were eligible for the BE VITAL (NCT04009499) open-label extension. For BE OPTIMAL, the ADA 40 mg Q2W treatment arm served as an active reference. The BE OPTIMAL study was not powered for statistical comparisons of ADA to BKZ or PBO. Completion rates include patients that completed to Week 52/104/160 in BE OPTIMAL and Week 52/100/156 in BE COMPLETE not on randomized treatment (BE OPTIMAL Week 52: n=9 [1.1%], Week 104: n=8 [0.9%], Week 160: n=10 [1.2%]; BE COMPLETE Week 52: n=4 [1.0%], Week 100: n=2 [0.5%], Week 156: n=4 [1.0%]). 2 patients in BE COMPLETE were classified as ongoing at Week 52 as they did not have a visit for Week 52 but no formal discontinuation reason was reported. Permitted concomitant medications included, and were not limited to, non-steroidal anti-inflammatory drugs and conventional synthetic DMARDs within protocol guidelines; changes to permitted concomitant medications were allowed at any time after enrolment in BE VITAL, if deemed appropriate by the investigator. **[a]** Safety follow-up visit 20 weeks after the last dose for patients not enrolling in the extension study. **ACR50:** ≥50% improvement from baseline in American College of Rheumatology response criteria; **ADA:** adalimumab; **bDMARD:** biological disease modifying anti-rheumatic drug; **BKZ:** bimekizumab; **OLE:** open-label extension; **PBO:** placebo; **Q2W:** every 2 weeks; **Q4W:** every 4 weeks; **TNFi-IR:** prior inadequate response or intolerance to tumour necrosis factor inhibitors.

Methods (2/2)

- Safety outcomes are reported for the BE OPTIMAL 'All Patients' group and the BE OPTIMAL and BE COMPLETE 'Bimekizumab Total' groups (bimekizumab-randomized patients and patients who switched from placebo to bimekizumab at Week 16 [placebo/bimekizumab]; this includes all patients in BE COMPLETE) up to 3 years from the baseline of the initial studies.
- Efficacy outcomes are reported for bimekizumab-randomized and placebo/bimekizumab patients separately up to Year 1 then for the Bimekizumab Total group (which includes bimekizumab-randomized and placebo/bimekizumab patients) up to Year 3 from the baseline of the initial studies.
- Modified non-responder (mNRI; binary), non-responder (NRI; binary), and multiple imputation (MI; continuous) were used to impute missing data.
  - mNRI considered all visits following discontinuation due to adverse event (AE) or lack of efficacy as non-response; all other missing data were imputed with MI and the response derived from the imputed values.[1]

1. DiRuggiero D. J Clin Aesthet Dermatol 2024;17:S15-s24. **AE:** adverse event; **MI:** multiple imputation; **mNRI:** modified NRI; **NRI:** non-responder imputation.

Safety to Year 3 (1/3)

- Bimekizumab was well tolerated in patients with PsA with a safety profile up to 3 years that was consistent with previous reports.

	BE OPTIMAL (bDMARD-naïve)		BE COMPLETE (TNFi-IR)
**n[a] (%)**<br>**EAIR/100 PY (95% CI)**	**BKZ 160 mg Q4W Total[b] n=702 (1,794.3 PY)**	**BKZ 160 mg Q4W All Patients[c] N=823 (2,022.1 PY)**	**BKZ 160 mg Q4W Total[b] N=388 (985.3 PY)**
Any TEAE	650 (92.6)<br>168.1 (155.5–181.6)	755 (91.7)<br>164.2 (152.7–176.3)	318 (82.0)<br>88.6 (79.1–98.9)
Serious TEAEs[d]	114 (16.2)<br>6.9 (5.7–8.3)	122 (14.8)<br>6.5 (5.4–7.8)	52 (13.4)<br>5.7 (4.2–7.4)
Study discontinuations due to TEAEs	60 (8.5)<br>3.4 (2.6–4.4)	65 (7.9)<br>3.3 (2.5–4.2)	27 (7.0)<br>2.8 (1.8–4.0)
Permanent treatment discontinuations due to TEAEs	65 (9.3)<br>3.7 (2.8–4.7)	70 (8.5)<br>3.5 (2.7–4.4)	29 (7.5)<br>3.0 (2.0–4.3)
Drug-related TEAEs	324 (46.2)<br>27.4 (24.5–30.5)	365 (44.3)<br>26.9 (24.2–29.8)	130 (33.5)<br>17.1 (14.3–20.3)
Severe TEAEs	61 (8.7)<br>3.5 (2.7–4.6)	66 (8.0)<br>3.4 (2.6–4.3)	35 (9.0)<br>3.7 (2.6–5.2)
Death	2 (0.3)[e,f]<br>0.1 (0.0–0.4)	3 (0.4)[e,f]<br>0.2 (0.0–0.4)	1 (0.3)[e,g]<br>0.1 (0.0–0.6)

	BE OPTIMAL (bDMARD-naïve)		BE COMPLETE (TNFi-IR)
**n[a] (%)**<br>**EAIR/100 PY (95% CI)**	**BKZ 160 mg Q4W Total[b] n=702 (1,794.3 PY)**	**BKZ 160 mg Q4W All Patients[c] N=823 (2,022.1 PY)**	**BKZ 160 mg Q4W Total[b] N=388 (985.3 PY)**
Most frequently reported TEAEs (five most common TEAEs in any BKZ-treated group at the Year 3 data cut)			
SARS-CoV-2 (COVID-19) infection[h]	205 (29.2)<br>13.3 (11.5–15.2)	240 (29.2)<br>13.9 (12.2–15.7)	72 (18.6)<br>8.1 (6.4–10.2)
Nasopharyngitis	125 (17.8)<br>8.0 (6.7–9.6)	139 (16.9)<br>7.8 (6.6–9.3)	44 (11.3)<br>4.8 (3.5–6.5)
Upper respiratory tract infection	102 (14.5)<br>6.2 (5.1–7.6)	113 (13.7)<br>6.1 (5.0–7.4)	38 (9.8)<br>4.1 (2.9–5.6)
Urinary tract infection	83 (11.8)<br>5.0 (4.0–6.2)	90 (10.9)<br>4.8 (3.8–5.9)	37 (9.5)<br>4.0 (2.8–5.6)
Oral candidiasis	73 (10.4)<br>4.3 (3.4–5.5)	82 (10.0)<br>4.3 (3.4–5.4)	34 (8.8)<br>3.6 (2.5–5.1)

Safety set. Data reported to 3 years (Week 156). **[a]** 'n' denotes the number of patients reporting at least one of the respective TEAE; **[b]** BKZ Total group includes BKZ-randomized patients and PBO patients that switched to BKZ at Week 16; includes events after switch only; **[c]** All Patients group includes all patients that had received at least one dose of BKZ, including patients that switched to BKZ from the reference arm (adalimumab) at Week 52; includes events after switch only; **[d]** Serious TEAEs met one or more of the following criteria: death, life-threatening event, significant or persistent disability/incapacity, congenital anomaly/birth defect (including in a foetus), important medical event, or initial inpatient hospitalization or prolonged hospitalization; **[e]** Considered not related to the study drug; **[f]** Earlier deaths as previously described;13 one additional death between Year 2 and Year 3 due to cardiac arrest in a 66-year old patient randomized to bimekizumab at baseline with a history of cardiovascular disease and multiple concomitant medications; **[g]** Sudden death in Year 1 as previously described;[1] **[h]** Specific terms for SARS-CoV-2 (COVID-19) infections were not available in the MedDRA v19.0; confirmed or suspected cases were identified using the preferred terms "corona virus infection" and "coronavirus test positive". 1. Mease PJ. Rheumatol Ther 2024;11(5):1363–82. **bDMARD:** biologic disease-modifying antirheumatic drug; **BKZ:** bimekizumab; **CI:** confidence interval; **EAIR:** exposure-adjusted incidence rate; **PBO:** placebo; **PY:** patient-years; **Q4W:** every four weeks; **TEAE:** treatment-emergent adverse event; **TNFi-IR:** prior inadequate response or intolerance to tumor necrosis factor inhibitors.

Safety to Year 3 (2/3)

- The majority of fungal infections were mild or moderate, none were systemic and the rate of discontinuations due to fungal infections was low.
- The incidence of fungal infections did not increase with treatment up to 3 years.

	BE OPTIMAL (bDMARD-naïve)		BE COMPLETE (TNFi-IR)
**n[a] (%)** **EAIR/100 PY (95% CI)**	**BKZ 160 mg Q4W Total[b] n=702 (1,794.3 PY)**	**BKZ 160 mg Q4W All Patients[c] N=823 (2,022.1 PY)**	**BKZ 160 mg Q4W Total[b] N=388 (985.3 PY)**
Safety topics of interest			
Serious infections	25 (3.6) 1.4 (0.9–2.1)	28 (3.4) 1.4 (0.9–2.0)	13 (3.4) 1.3 (0.7–2.3)
Opportunistic infections[d]	18 (2.6) 1.0 (0.6–1.6)	19 (2.3) 1.0 (0.6–1.5)	3 (0.8) 0.3 (0.1–0.9)
Active tuberculosis	0	0	0
Fungal infections	144 (20.5) 9.2 (7.8–10.9)	163 (19.8) 9.2 (7.9–10.8)	52 (13.4) 5.8 (4.3–7.6)
Candida infection	97 (13.8) 5.9 (4.8–7.2)	106 (12.9) 5.7 (4.7–6.9)	37 (9.5) 4.0 (2.8–5.5)
Oral candidiasis	73 (10.4) 4.3 (3.4–5.5)	82 (10.0) 4.3 (3.4–5.4)	34 (8.8) 3.6 (2.5–5.1)
Fungal infections NEC	61 (8.7) 3.6 (2.8–4.6)	71 (8.6) 3.7 (2.9–4.7)	18 (4.6) 1.9 (1.1–3.0)

Safety set. Data reported to 3 years (Week 156). **[a]** 'n' denotes the number of patients reporting at least one of the respective TEAE; **[b]** BKZ Total group includes BKZ-randomized patients and PBO patients that switched to BKZ at Week 16; includes events after switch only; **[c]** All Patients group includes all patients that had received at least one dose of BKZ, including patients that switched to BKZ from the reference arm (adalimumab) at Week 52; includes events after switch only; **[d]** No cases of histoplasmosis, blastomycosis or coccidioidomycosis were reported. **bDMARD:** biologic disease-modifying antirheumatic drug; **BKZ:** bimekizumab; **CI:** confidence interval; **EAIR:** exposure-adjusted incidence rate; **NEC:** not elsewhere classified; **PBO:** placebo; **PY:** patient-years; **Q4W:** every four weeks; **TEAE:** treatment-emergent adverse event; **TNFi-IR:** prior inadequate response or intolerance to tumor necrosis factor inhibitors.

Safety to Year 3 (3/3)

- Incidence rates for TEAEs of interest were low, such as neutropenia, major adverse cardiovascular events (MACE), suicidal ideation and behaviour, uveitis, and inflammatory bowel disease (IBD).
- The long-term safety profile was generally consistent between bDMARD-naïve and TNFi-IR patients.

n[a] (%)<br>EAIR/100 PY (95% CI)	BE OPTIMAL (bDMARD-naïve)		BE COMPLETE (TNFi-IR)
	**BKZ 160 mg Q4W Total[b] n=702 (1,794.3 PY)**	**BKZ 160 mg Q4W All Patients[c] N=823 (2,022.1 PY)**	**BKZ 160 mg Q4W Total[b] N=388 (985.3 PY)**
Safety topics of interest			
Any neutropenia	22 (3.1)[d]<br>1.3 (0.8–1.9)	22 (2.7)[d]<br>1.1 (0.7–1.7)	13 (3.4)[e]<br>1.4 (0.7–2.3)
Serious hypersensitivity reactions	0	0	1 (0.3)[f]<br>0.1 (0.0–0.6)
Any administration/injection site reaction[g]	24 (3.4)<br>1.4 (0.9–2.0)	28 (3.4)<br>1.4 (0.9–2.1)	8 (2.1)<br>0.8 (0.4–1.6)
Elevated liver enzymes, n/N (%)[h]	71/702 (10.1)<br>4.3 (3.3–5.4)	80/823 (9.7)<br>4.2 (3.4–5.3)	38/388 (9.8)<br>4.1 (2.9–5.7)
>3× ULN ALT/AST	34/701 (4.9)<br>2.0 (1.4–2.7)	39/822 (4.7)<br>2.0 (1.4–2.7)	17/388 (4.4)<br>1.8 (1.0–2.8)
>5× ULN ALT/AST	9/701 (1.3)<br>0.5 (0.2–1.0)	10/822 (1.2)<br>0.5 (0.2–0.9)	8/388 (2.1)<br>0.8 (0.4–1.6)
Adjudicated MACE	7 (1.0)<br>0.4 (0.2–0.8)	9 (1.1)<br>0.5 (0.2–0.9)	2 (0.5)<br>0.2 (0.0–0.7)
Malignancies,[i] excluding nonmelanoma skin cancer	9 (1.3)<br>0.5 (0.2–1.0)	9 (1.1)<br>0.5 (0.2–0.9)	10 (2.6)<br>1.0 (0.5–1.9)
Adjudicated suicidal ideation and behaviour[j]	2 (0.3)<br>0.1 (0.0–0.4)	2 (0.2)<br>0.1 (0.0–0.4)	0
Adjudicated IBD[k]	5 (0.7)[l]<br>0.3 (0.1–0.7)	7 (0.9)[l]<br>0.4 (0.1–0.7)	1 (0.3)[m]<br>0.1 (0.0–0.6)
Uveitis[n]	4 (0.6)<br>0.2 (0.1–0.6)	4 (0.5)<br>0.2 (0.1–0.5)	0

Safety set. Data reported to 3 years (Week 156). **[a]** 'n' denotes the number of patients reporting at least one of the respective TEAE; **[b]** BKZ Total group includes BKZ-randomized patients and PBO patients that switched to BKZ at Week 16; includes events after switch only; **[c]** All Patients group includes all patients that had received at least one dose of BKZ, including patients that switched to BKZ from the reference arm (adalimumab) at Week 52; includes events after switch only; **[d]** Includes 20 patients with neutropenia and 2 patients with neutrophil count decreased; **[e]** Includes 8 patients with neutropenia and 6 patients with neutrophil count decreased; **[f]** One case of dermatitis classed as serious due to the patient requiring hospitalization; **[g]** Identified using the high-level terms "administration site reactions NEC" and "injection site reactions"; **[h]** Elevated liver enzymes included the following preferred terms reported as adverse events: increased/abnormal levels of ALT, AST, blood bilirubin, gamma-glutamyltransferase, hepatic enzyme, liver function test, total bile acids or transaminases; **[i]** Malignancies were reported in an additional 5 patients in BE OPTIMAL and 1 patient in BE COMPLETE when including nonmelanoma skin cancer; **[j]** No cases of completed suicide reported; **[k]** Cases deemed definite or probable IBD by the investigator; **[l]** One patient with prior history of IBD; **[m]** Patient did not have prior history of IBD; **[n]** Uveitis TEAEs identified using the preferred terms "autoimmune uveitis", "iridocyclitis", "iritis", and "uveitis". **ALT:** alanine aminotransferase; **AST:** aspartate aminotransferase; **bDMARD:** biologic disease-modifying antirheumatic drug; **BKZ:** bimekizumab; **CI:** confidence interval; **EAIR:** exposure-adjusted incidence rate; **IBD:** inflammatory bowel disease; **MACE:** major adverse cardiac event; **PBO:** placebo; **PY:** patient-years; **Q4W:** every four weeks; **TEAE:** treatment-emergent adverse event; **TNFi-IR:** prior inadequate response or intolerance to tumor necrosis factor inhibitors; **ULN:** upper limit of normal.

Joint Outcomes to Year 3 (mNRI, OC)

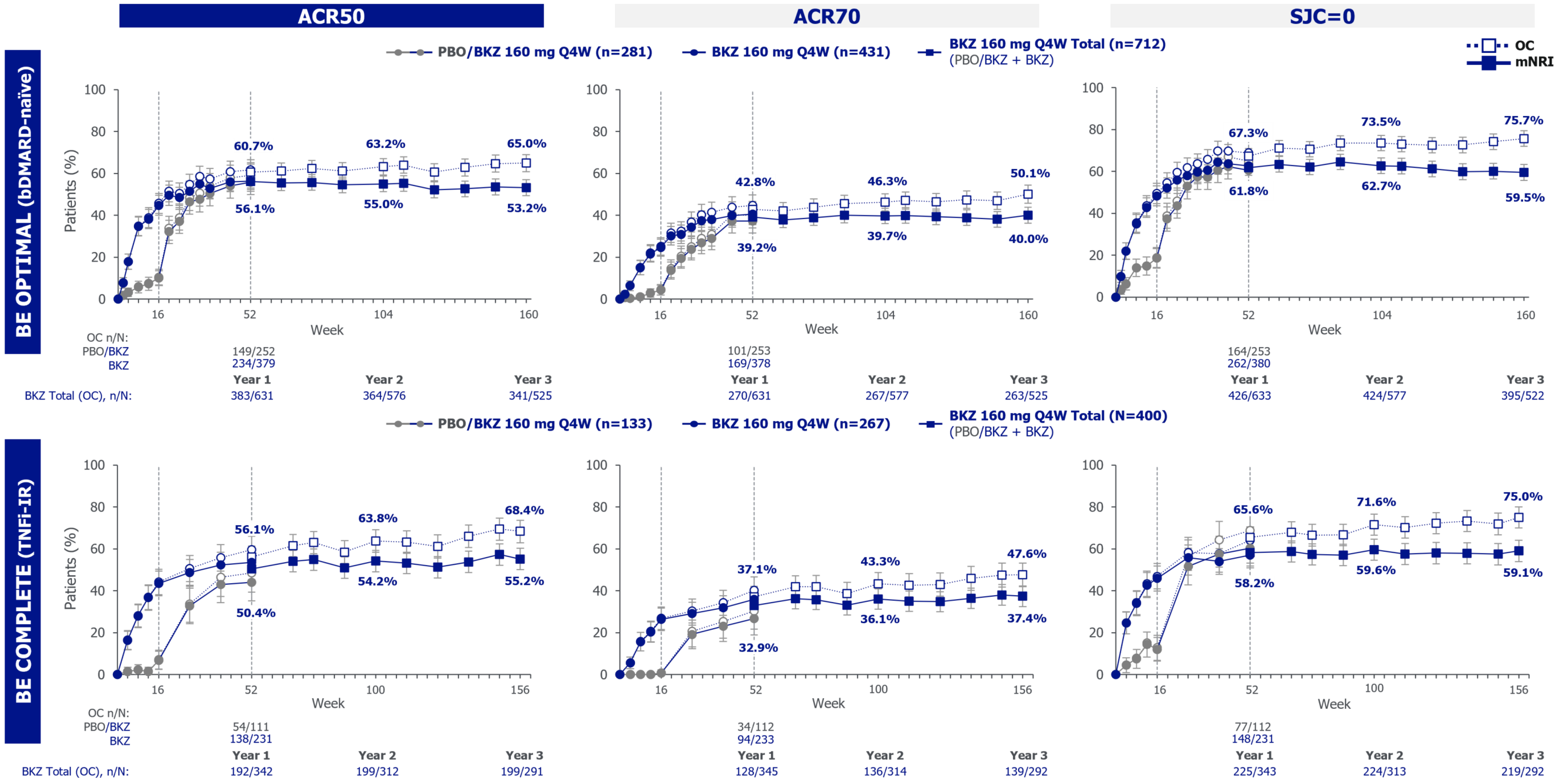

Randomized set. Bimekizumab Total group includes bimekizumab-randomized patients and placebo-randomized patients that switched to bimekizumab at Week 16. Data reported to 3 years (Week 160 in BE OPTIMAL and Week 156 in BE COMPLETE). mNRI considered all visits following discontinuation due to AEs or lack of efficacy as non-response; all other missing data were imputed with multiple imputation and the response derived from the imputed values. Error bars represent 95% CIs. **ACR50/70:** ≥50/70% improvement from baseline in American College of Rheumatology response criteria; **AE:** adverse event; **bDMARD:** biologic disease-modifying antirheumatic drug; **BKZ:** bimekizumab; **CI:** confidence interval; **mNRI:** modified non-responder imputation; **OC:** observed case; **PBO:** placebo; **Q4W:** every four weeks; **SJC:** swollen joint count; **TNFi-IR:** prior inadequate response or intolerance to tumor necrosis factor inhibitors.

Skin and Nail Outcomes to Year 3 (mNRI, OC)

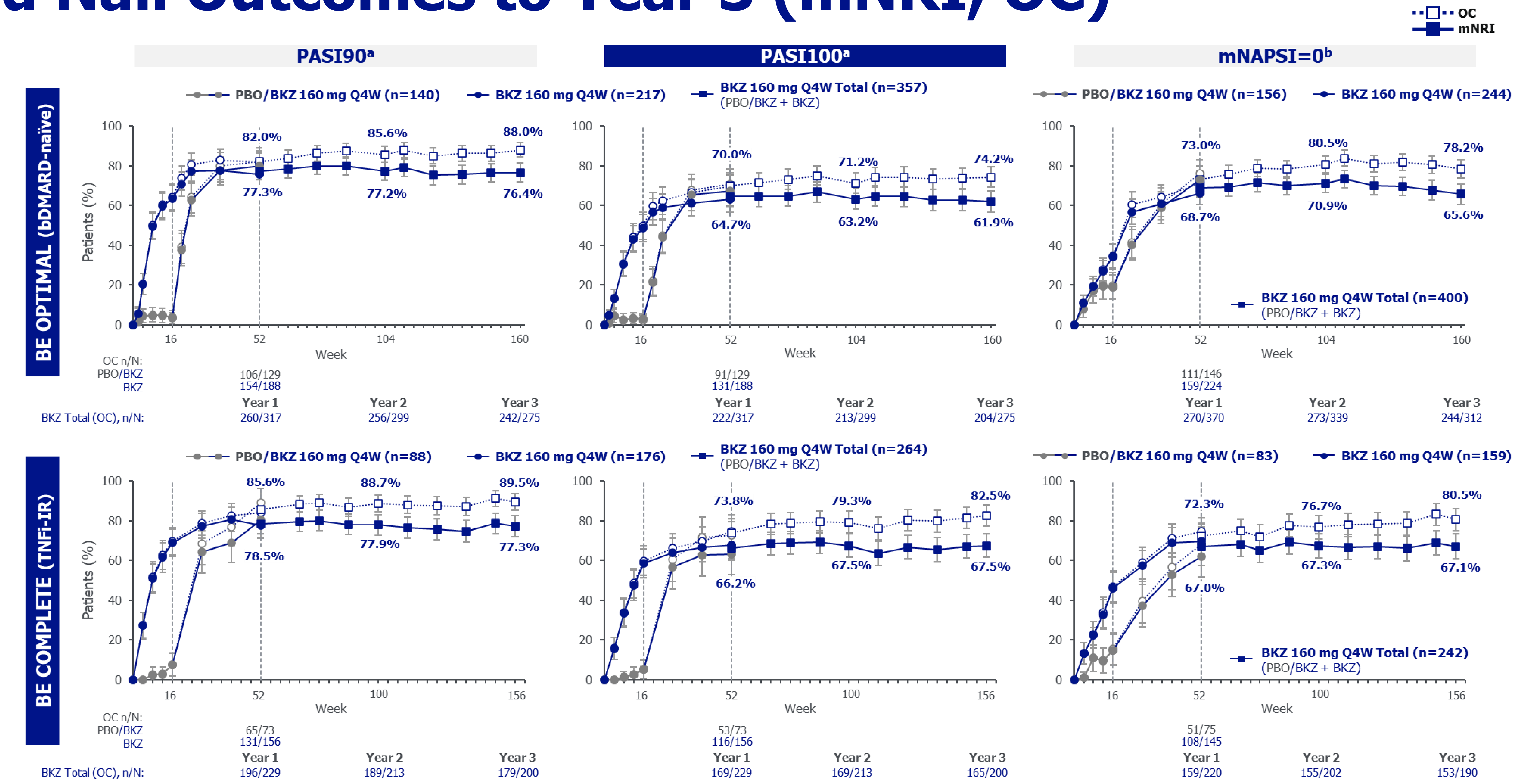

Randomized set. Bimekizumab Total group includes bimekizumab-randomized patients and placebo-randomized patients that switched to bimekizumab at Week 16. Data reported to 3 years (Week 160 in BE OPTIMAL and Week 156 in BE COMPLETE). mNRI considered all visits following discontinuation due to AEs or lack of efficacy as non-response; all other missing data were imputed with multiple imputation and the response derived from the imputed values. Error bars represent 95% CIs. **[a]** In patients with ≥3% BSA at baseline; **[b]** In patients with nail psoriasis (mNAPSI >0) at baseline. **AE:** adverse event; **bDMARD:** biologic disease-modifying antirheumatic drug; **BKZ:** bimekizumab; **BSA:** body surface area; **CI:** confidence interval; **mNAPSI:** modified Nail Psoriasis Severity Index; **mNRI:** modified non-responder imputation; **OC:** observed case; **PASI90/100:** ≥90/100% improvement from baseline in Psoriasis Area and Severity Index; **PBO:** placebo; **Q4W:** every four weeks; **TNFi-IR:** prior inadequate response or intolerance to tumour necrosis factor inhibitors.

Composite Outcomes to Year 3 (mNRI, OC)

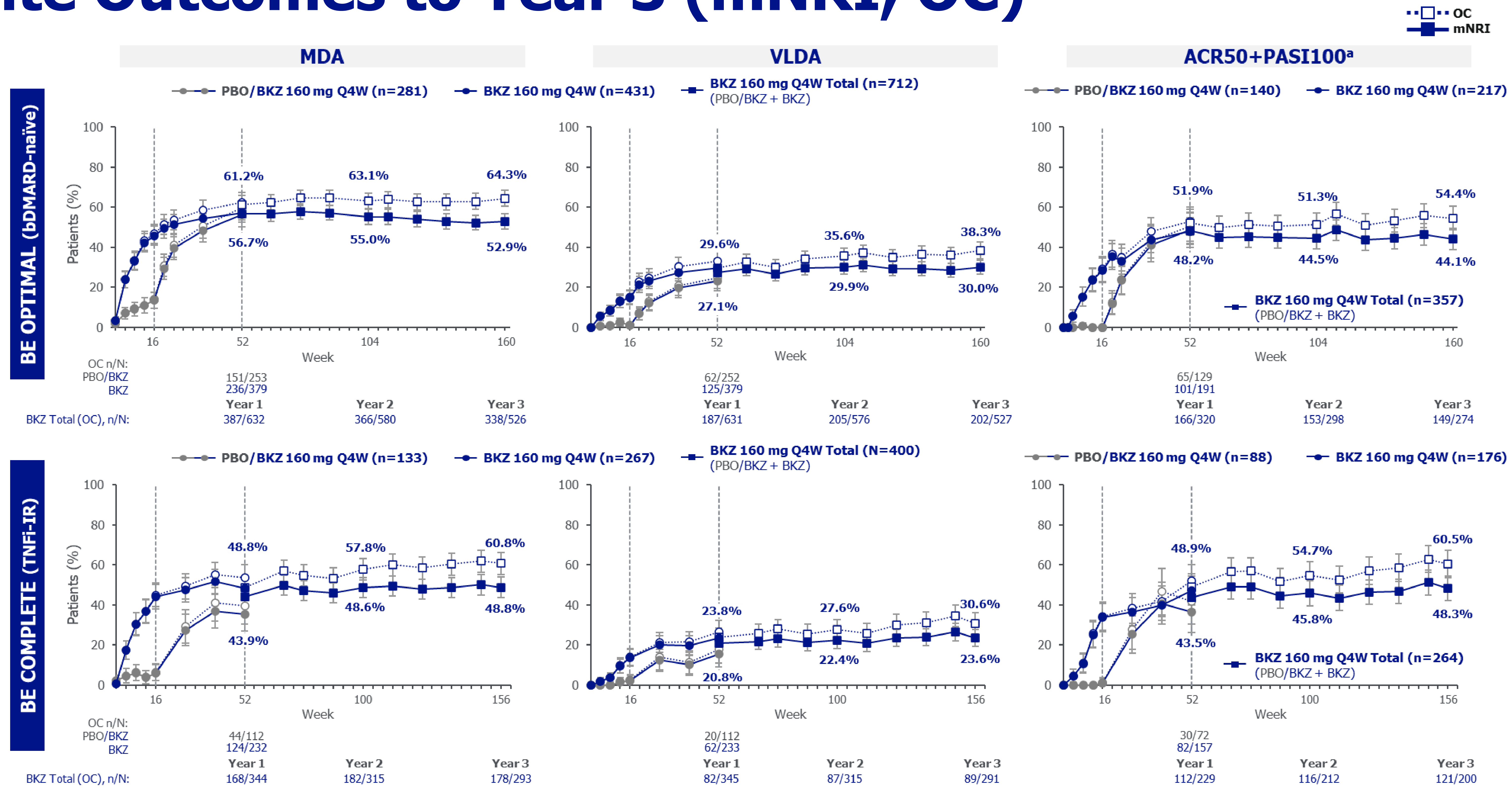

Randomized set. Bimekizumab Total group includes bimekizumab-randomized patients and placebo-randomized patients that switched to bimekizumab at Week 16. Data reported to 3 years (Week 160 in BE OPTIMAL and Week 156 in BE COMPLETE). mNRI considered all visits following discontinuation due to AEs or lack of efficacy as non-response; all other missing data were imputed with multiple imputation and the response derived from the imputed values. Error bars represent 95% CIs. **[a]** In patients with ≥3% BSA at baseline. **ACR50+PASI100:** ≥50% improvement from baseline in American College of Rheumatology response criteria + 100% improvement from baseline in Psoriasis Area and Severity Index; **AE:** adverse event; **bDMARD:** biologic disease-modifying antirheumatic drug; **BKZ:** bimekizumab; **BSA:** body surface area; **CI:** confidence interval; **MDA:** minimal disease activity; **mNRI:** modified non-responder imputation; **OC:** observed case; **PBO:** placebo; **Q4W:** every four weeks; **TNFi-IR:** prior inadequate response or intolerance to tumor necrosis factor inhibitors; **VLDA:** very low disease activity.

Patient-Reported Outcomes to Year 3 (mNRI, OC)

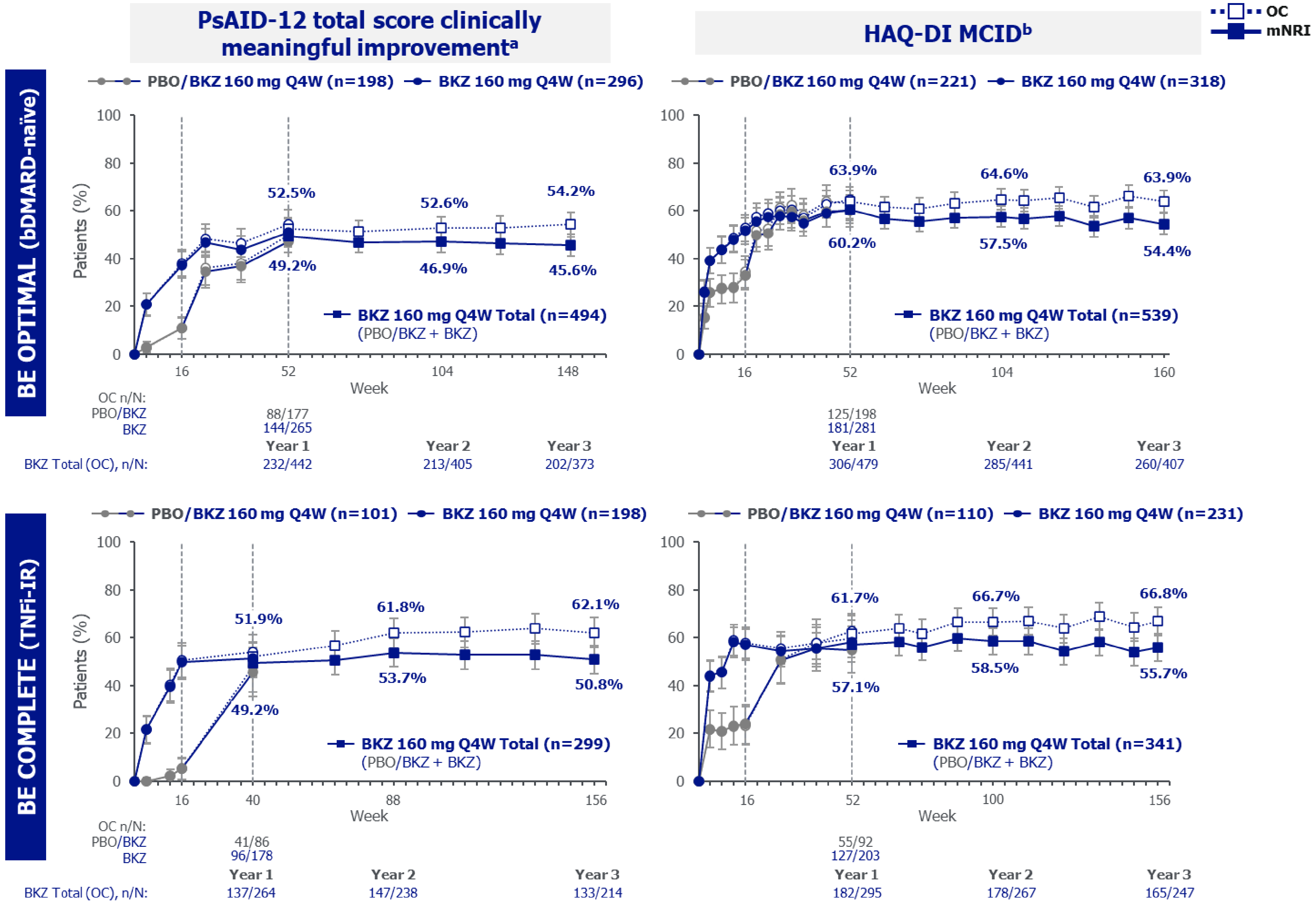

Randomized set. Bimekizumab Total group includes bimekizumab-randomized patients and placebo-randomized patients that switched to bimekizumab at Week 16. Data reported to 3 years (Week 148 for PsAID-12 or Week 160 for HAQ-DI in BE OPTIMAL and Week 156 in BE COMPLETE). mNRI considered all visits following discontinuation due to AEs or lack of efficacy as non-response; all other missing data were imputed with multiple imputation and the response derived from the imputed values. Error bars represent 95% CIs. **[a]** PsAID-12 clinically meaningful improvement defined as decrease from baseline ≥3 in patients with PsAID-12 ≥3 at baseline; Data reported at Week 40 (Year 1) and Week 88 (Year 2) for BE COMPLETE; **[b]** HAQ-DI MCID defined as decrease from baseline ≥0.35 in patients with HAQ-DI ≥0.35 at baseline; Data reported at Week 100 (Year 2) for BE COMPLETE. **AE:** adverse event; **bDMARD:** biologic disease-modifying antirheumatic drug; **BKZ:** bimekizumab; **CI:** confidence interval; **HAQ-DI:** Health Assessment Questionnaire-Disability Index; **MCID:** minimal clinically important difference; **mNRI:** modified non-responder imputation; **OC:** observed case; **PBO:** placebo; **PsAID-12:** Psoriatic Arthritis Impact of Disease 12-Item Questionnaire; **Q4W:** every four weeks; **TNFi-IR:** prior inadequate response or intolerance to tumour necrosis factor inhibitors.

Discussion & Conclusion

- Bimekizumab was **well tolerated** with longer-term treatment and had a safety profile consistent with that previously observed in studies of bimekizumab in PsA during phase 3 studies.[1]
- Bimekizumab treatment resulted in **sustained and consistent high levels of efficacy** across the full spectrum of disease.
- Consistent, sustained clinical efficacy was observed up to 3 years **in both bDMARD-naïve and TNFi-IR patients** with active PsA.
- These data support the suitability of bimekizumab for the **long-term treatment** of bDMARD-naïve and TNFi-IR patients with PsA.

1. Mease PJ. Rheumatol Ther 2024;11(5):1363–82. **bDMARD:** biologic disease-modifying antirheumatic drug; **PsA:** psoriatic arthritis; **TNFi-IR:** prior inadequate response or intolerance to tumour necrosis factor inhibitors.
