# Supplementary material for: Bimekizumab safety and efficacy in patients with psoriatic arthritis: 3-year results from two phase 3 studies
Source: Rheumatology (Oxford). 2026 Mar 16;65(5):keag118. doi: 10.1093/rheumatology/keag118 (PMC13198880; doi:10.1093/rheumatology/keag118)
Supplement: keag118_Supplementary_Data [file keag118_supplementary_data.zip › rhe-25-2713-File009.docx]

**Supplementary Data S1. Local institutional review board and independent ethics committee names for BE OPTIMAL and BE COMPLETE**

| **BE OPTIMAL** | **BE COMPLETE** |
| --- | --- |
| **Australia** | |
| Monash Medical Center  246 Clayton Road  Level 4 Block  3168 Clayton  Victoria  Australia  Site: 30002 | CALHN Research Office - SA Health Government  Level 3, Roma Mitchell House  136 North Terrace  South Australia  Site: 30006 |
| CALHN Research Office - SA Health Government  Level 3, Roma Mitchell House  136 North Terrace  South Australia  Site: 30006 | Bellberry Limited  123 Glen Osmond Road  Eastwood  5063 South Australia  Sites: 30007, 30005 |
| Bellberry Limited  123 Glen Osmond Road  Eastwood 5063  South Australia  Site 30003 | – |
| Bellberry Limited  123 Glen Osmond Road  Eastwood 5063  South Australia  Site 30005 | – |
| Bellberry Limited  123 Glen Osmond Road  Eastwood 5063  South Australia  Site 30007 | – |
| Bellberry Limited  123 Glen Osmond Road  Eastwood 5063  South Australia  Site 30008 | – |
| **Belgium** | |
| UZ Leuven - Campus Gasthuisberg  Ethische Commissie Onderzoek UZ/KU Leuven  Herestraat 49  3000 Leuven  Belgium  Site: 40002 | – |
| CHU Ambroise Paré  Comité d'éthique  Boulevard Kennedy 2  7000 Mons  Belgium  Site: 40059 | – |
| CHU de Liège – Sart Tilman  Comité d’éthique Hospitalo-Facultaire Universitaire de Liège  Domaine Universitaire du Sart Tilman, Bâtiment B35  4000 Liège  Belgium  Sites: 40060, 40003 | – |
| **Canada** | |
| Advarra  372 Hollandview Trail Suite 300  Ontario L4G 0A5 Aurora  Canada  Sites: 50041, 50042, 50044 | Advarra  372 Hollandview Trail Suite 300  Ontario L4G 0A5 Aurora  Canada  Sites: 50042, 50044 |
| Nova Scotia Health Authority Research Ethics Board  5790 University Avenue Room 118  Nova Scotia B3H 1V7 Halifax  Canada  Site: 50043 | Nova Scotia Health Authority Research Ethics Board  5790 University Avenue Room 118  Nova Scotia B3H 1V7 Halifax  Canada  Site: 50043 |
| **Czech Republic** | |
| CTCenter MaVe s.r.o.  Eticka komise  Na Sibeniku 914/1 779 00 Olomouc  Czech Republic  Site: 40064 | Fakultni nemocnice v Motole  Eticka komise (Central Ethic Committee)  V Uvalu 84 Praha 5 150 06 Praha 5  Czech Republic  Sites: 40009, 40012, 40063, 40066 |
| Fakultni nemocnice v Motole  Eticka komise  V Uvalu 84 Praha 5 150 06 Praha 5  Czech Republic  Sites: 40009, 40010, 40012, 40013, 40014, 40015, 40061, 40062, 40063, 40064, 40065, 40066 | Revmatologicky ustav  Eticka komise  Na Slupi 4 128 50 Praha 2  Czech Republic  Site: 40066 |
| Revmatologicky ustav  Eticka komise  Na Slupi 4 128 50 Praha 2  Czech Republic  Site: 40066 | – |
| **France** | |
| CPP Sud Est III  Bâtiment Pinel (Central Ethic Committee)  59 Boulevard Pinel  Bron 69500  France  Sites: 40019, 40067, 40068, 40069, 40070 | – |
| **Germany** | |
| Ethikkommission an der Medizinischen Fakultät der Universität Leipzig  Ethic Committee  Käthe-Kollwitz-Straße 82 Karl-Sudhoff-Institut Sachsen 4109 Leipzig  Germany  Site: 40078 | Ethikkommission an der Medizinischen Fakultät der  Universität Leipzig  Ethic Committee  Käthe-Kollwitz-Straße 82 Karl-Sudhoff-Institut Sachsen  4109 Leipzig  Germany  Site: 40078 |
| Ethikkommission der Ärztekammer Hamburg Ethic Committee  Weidestraße 122b Hamburg 22083 Hamburg  Germany  Sites: 40029, 40071 | Ethikkommission der Ärztekammer Hamburg Ethic  Committee  Weidestraße 122b Hamburg 22083 Hamburg  Germany  Sites: 40029, 40071 |
| Ethikkommission an der Medizinischen Fakultät Ernst-Moritz-Arndt-Universität Greifswald  Institut für Pharmakologie  Felix-Hausdorff-Str. 3 Mecklenburg-Vorpommern 17487 Greifswald  Germany  Site: 40074 | Ethikkommission der Ärztekammer Nordrhein Ethics  Committee  Tersteegenstraße 9 Nordrhein-Westfalen 40474 Düsseldorf  Germany  Site: 40026 |
| Ethikkommission der Ärztekammer Nordrhein Ethics Committee  Tersteegenstraße 9 Nordrhein-Westfalen 40474 Düsseldorf  Germany  Site: 40026  Ethikkommission der Ärztekammer Westfalen-Lippe und der medizinischen Fakultät der WWU Münster  Ethics Committee  Gartenstr. 210 - 214 Nordrhein-Westfalen 48147 Münster  Germany  Site: 40027 | Ethikkommission der Medizinischen Fakultät der  Friedrich-Alexander-Universität Erlangen-Nürnberg  Krankenhausstraße 12 Bayern 91054 Erlangen  Germany  Sites: 40023, Central EC |
| Ethikkommission der Ärztekammer Sachsen-Anhalt - IRB/IEC  IRB/IEC  Am Kirchtor 9 Sachsen-Anhalt 06108 Halle (Saale)  Germany  Site: 40348 | Ethikkommission der Landesärztekammer Brandenburg  Dreifertstraße 12 03044 Cottbus  Germany  Site: 40076 |
| Ethikkommission der Medizinischen Fakultät der Friedrich-Alexander-Universität Erlangen-Nürnberg  Krankenhausstraße 12 Bayern 91054 Erlangen  Germany  Site: 40023 (Central EC) | Ethikkommission des Fachbereichs Medizin der Goethe-  Universität  Ethic Committee  Theodor-Stern-Kai 7 Haus 1, 2. OG, Zi. 207 Hessen  60596 Frankfurt am Main  Germany  Site: 40117 |
| Ethikkommission der Landesärztekammer Brandenburg  Dreifertstraße 12 03044 Cottbus  Germany  Site: 40076 | – |
| Ethikkommission des Landes Berlin, Landesamt für Gesundheit und Soziales  Ethics Committe  Turmstraße 21 Haus A Berlin 10559 Berlin  Germany  Sites: 40025, 40028 | – |
| Ethikkommission des Fachbereichs Medizin der Goethe-Universität  Ethic Committee  Theodor-Stern-Kai 7 Haus 1, 2. OG, Zi. 207 Hessen 60596 Frankfurt am Main  Germany  Site: 40117 | – |
| **Hungary** | |
| Debreceni Egyetem Klinikai Központ, Regionális Intézményi és Kutatásetikai Bizottság  40332 Debrecen Nagyerdei krt. 98. Pf. 34.  Hungary  Site: 40032 | Csongrád Megyei Dr. Bugyi István Kórház Mozgasszervi  Rehabil. Osztaly  Sima F. u. 44-58 Csongrád 6600 Szentes  Hungary  Site: 40079 |
| Csongrád Megyei Dr. Bugyi István Kórház Mozgasszervi Rehabil. Osztaly  Sima F. u. 44-58 Csongrád 6600 Szentes  Hungary  Site: 40079 | Markhot Ferenc Oktatokorhaz es Rendelointezet -  Rheumatology  Rheumatology  Furdo u. 4. Heves 3300 Eger  Hungary  Site: 40030 |
| Markhot Ferenc Oktatokorhaz es Rendelointezet - Rheumatology  Rheumatology  Furdo u. 4. Heves 3300 Eger  Hungary  Site: 40030 | Pest Megyei Flór Ferenc Kórház Intézményi, Kutatásetikai  Bizottság2143, Kistarcsa Semmelweis tér 1.  Hungary  Site: 40082 |
| Vasútegészségügyi Kft. Intézeti Kutatásetikai Bizottság  1062 Budapest  Podmaniczky utca 109  Hungary  Site: 40080 | – |
| Fejér Megyei Szent György Egyetemi Oktató Kórház, Kutatásetikai Bizottság,  8000, Szekesfehervar  Seregelyesi ut 3  Hungary  Site: 40033 | – |
| Pest Megyei Flór Ferenc Kórház Intézményi, Kutatásetikai Bizottság 2143  Kistarcsa Semmelweis tér 1  Hungary  Site: 40082 | – |
| Medical Research Council Ethics Committee for Clinical Pharm  Alkotmány u. 25. Budapest  1054 Budapest  Hungary  Sites: 40081, 40083 | – |
| **Italy** | |
| Comitato Etico Catania 1 (Central Ethics Committee)  A.O.U. Policlinico Vittorio Emanuele Di Catania Via Santa Sofia, 78  95123 Catania  Italy  Site: 40084 | Comitato Etico Catania 1 (Central Ethic Committee)  A.O.U. Policlinico Vittorio Emanuele Di Catania Via  Santa Sofia, 78  95123 Catania  Italy  Site: 40084 |
| COMITATO ETICO DELL'AREA VASTA EMILIA NORD - Segreteria Locale di Reggio Emila  c/o AUSL – IRCCS di Reggio Emilia Edificio Spallanzani  Viale Umberto I, 50  42100 Reggio Emilia  Italy  Site: 40086 | COMITATO ETICO DELL'AREA VASTA EMILIA  NORD - Segreteria Locale di Reggio Emila  c/o AUSL – IRCCS di Reggio Emilia Edificio Spallanzani  Viale Umberto I, 50  42100 Reggio Emilia  Italy  Site: 40086 |
| Comitato Etico Area 1  ASST Fatebenefratelli Sacco  Via GB Grassi 74  20157 Milano  Italy  Site: 40087 | Comitato Etico Area 1  ASST Fatebenefratelli Sacco, Via GB Grassi 74  20157 Milano  Italy  Site: 40087 |
| **Japan** | |
| Hokkaido University Hospital Institutional Review Board  Kita 14, Nishi 5, Kita-ku  Sapporo, Hokkaido, 060-8648  Japan  Site: 20031 | Hokkaido University Hospital Institutional Review Board  Kita 14, Nishi 5, Kita-ku  Sapporo, Hokkaido, 060-8648  Japan  Site: 20031 |
| Nagoya City University Institutional Review Board  1 Kawasumi, Mizuhocho, Mizuho-ku  Nagoya, Aichi, 467-8602  Japan  Site: 20033 | Nagoya City University Institutional Review Board  1 Kawasumi, Mizuhocho, Mizuho-ku  Nagoya, Aichi, 467-8602,  Japan  Site: 20033 |
| Juntendo University Hospital Institutional Review Board  3-1-3, Hongo  Bunkyo-ku, Tokyo, 113-8431  Japan  Site: 20035 | Saitama medical University Hospital Institutional Review  Board  38 Morohongo, Moroyama-machi,  Iruma-gun, Saitama, 350-0495,  Japan  Site: 20039 |
| Kochi Medical School Hospital Institutional Review Board  185-1 Kohasu, Oko-cho  Nankoku-shi, Kochi, 783-8505  Japan  Site: 20038 | Kochi Medical School Hospital Institutional Review  Board  185-1 Kohasu, Oko-cho,  Nankoku-shi, Kochi, 783-8505  Japan  Site: 20038 |
| Kagawa University Hospital Institutional Review Board  1750-1 Ikenobe, Miki-cho  Kita-gun, Kagawa, 761-0793  Japan  Site: 20045 | Kagawa University Hospital Institutional Review Board  1750-1 Ikenobe, Miki-cho  Kita-gun, Kagawa, 761-0793  Japan  Site: 20045 |
| Jichi Medical University Saitama Medical Center Institutional Review Board  1-847, Amanumacho, Omiya-ku  Saitama, Saitama, 330-8503  Japan  Site: 20048 | Jichi Medical University Saitama Medical Center  Institutional Review Board  1-847, Amanumacho, Omiya-ku,  Saitama, Saitama, 330-8503,  Japan  Site: 20048 |
| Hospital of the University of Occupational and Environmental Health, Japan Institutional Review Board  1-1 Iseigaoka, Yahatanishi-ku  Kitakyushu, Fukuoka, 807-8556  Japan  Site: 20049 | Hospital of the University of Occupational and  Environmental Health, Japan Institutional Review Board  1-1 Iseigaoka, Yahatanishi-ku,  Kitakyushu, Fukuoka, 807-8556  Japan  Site: 20049 |
| St. Luke's International Hospital Institutional Review Board  9-1 Akashicho  Chuo-ku, Tokyo, 104-8560  Japan  Site: 20030 | St. Luke's International Hospital Institutional Review  Board  9-1 Akashicho,  Chuo-ku, Tokyo, 104-8560  Japan  Site: 20030 |
| Osaka University Hospital Institutional Review Board  2-15 Yamadaoka  Suita, Osaka, 565-0871  Japan  Site: 20032 | Osaka University Hospital Institutional Review Board  2-15 Yamadaoka,  Suita, Osaka, 565-0871,  Japan  Site: 20032 |
| National Hospital Organization Osaka Minami Medical Center Institutional Review Board  2-1 Kidohigashimachi  Kawachinagano, Osaka, 586-8521  Japan  Site: 20036 | National Hospital Organization Osaka Minami Medical  Center Institutional Review Board  2-1 Kidohigashimachi,  Kawachinagano, Osaka, 586-8521,  Japan  Site: 20036 |
| Osaka City University Hospital Institutional Review Board  1-5-7 Asahimachi, Abeno-ku  Osaka, Osaka, 545-8586  Japan  Site: 20041 | Osaka Metropolitan University Hospital Institutional  Review Board  1-5-7 Asahimachi, Abeno-ku  Osaka, Osaka, 545-8586,  Japan  Site: 20041 |
| Sasebo Chuo Hospital Institutional Review Board  15 Yamato-cho  Sasebo, Nagasaki, 857-1195  Japan  Site: 20042 | Sasebo Chuo Hospital Institutional Review Board  15 Yamato-cho,  Sasebo, Nagasaki, 857-1195,  Japan  Site: 20042 |
| Nihon University Hospital’s Joint Institutional Review Board  30-1 Oyaguchi, Kami-cho  Itabashi-ku, Tokyo, 173-8610  Japan  Site: 20043 | Nihon University Hospital’s Joint Institutional Review  Board  30-1 Oyaguchi, Kami-cho  Itabashi-ku, Tokyo, 173-8610,  Japan  Site: 20043 |
| The Jikei University Hospital Institutional Review Board  3-19-18, Nishi-Shinbashi,  Minato-ku, Tokyo, 105-8471  Japan  Site: 20044 | The Jikei University Hospital Institutional Review Board  3-19-18, Nishi-Shinbashi,  Minato-ku, Tokyo, 105-8471,  Japan  Site: 20044 |
| Nippon Life Hospital Institutional Review Board  2-1-54 Enokojima, Nishi-ku  Osaka, Osaka, 550-0006  Japan  Site: 20046 | Nippon Life Hospital Institutional Review Board  2-1-54 Enokojima, Nishi-ku,  Osaka, Osaka, 550-0006,  Japan  Site: 20046 |
| **Poland** | |
| Komisja Bioetyczna przy Okregowej Radzie Lekarskiej Wielkopolskiej Izby Lekarskiej  Ul. Nowowiejskiego 51  61-734 Poznan  Poland  Sites: 40037, 40038, 40039, 40041, 40042, 40043, 40044, 40088, 40090, 40091, 40092, 40093, 40094, 40095, 40096, 40097, 40098, 40118, 40119 | Komisja Bioetyczna przy Okregowej Radzie Lekarskiej  Wielkopolskiej Izby Lekarskiej  Ul. Nowowiejskiego 51  61-734 Poznan  Poland  Sites: 40037, 40038, 40039, 40041, 40043, 40044, 40090,  40091,40097, 40098, 40118, 40119 |
| **Russian Federation** | |
| Leningrad Regional Clinical Hospital  Ethics Committe  45-49 Prospect Lunacharskogo  194291 Saint-Petersburg  Russian Federation  Site: 20001 | Leningrad Regional Clinical Hospital  Ethics Committe  45-49 Prospect Lunacharskogo  194291 Saint-Petersburg  Russian Federation  Site: 20001 |
| Clinical Rheumatological Hospital #25  Ethics Committe  Liter A, 30 Bolshaya Podyacheskaya Ulitsa  190068 Saint-Petersburg  Russian Federation  Site: 20003 | Indepentent Ethics Committee "Pharmexpert"  8/2, prospekt Nauki  Sankt-Peterburg  195257 Saint-Petersburg  Russian Federation  Site: 20004 |
| Indepentent Ethics Committee "Pharmexpert"  8/2, prospekt Nauki  Sankt-Peterburg  195257 Saint-Petersburg  Russian Federation  Site: 20004 | LLC Family Outpatient Clinic 4  Local Ethic Committee  33, Stantsionnaya st  141060 Korolev  Russian Federation  Site: 20005 |
| LLC Family Outpatient Clinic 4  Local Ethic Committee  33, Stantsionnaya str  141060 Korolev  Russian Federation  Site: 20005 | LLC "BioMed" - Administration  Administration  6, Nikitina street 600005 Vladimir  Russian Federation  Site: 20006 |
| LLC "BioMed" - Administration  Administration  6, Nikitina street 600005 Vladimir  Russian Federation  Site: 20006 | LLC Clinic of private security guards and detectives  84, Borovaya str  Sankt-Peterburg  192007 Saint Petersburg  Russian Federation  Site: 20009 |
| LLC Clinic of private security guards and detectives  84, Borovaya str  Sankt-Peterburg  192007 Saint Petersburg  Russian Federation  Site: 20009 | City Clinical Hospital # 1 n.a. N.I. Pirogov  Ethics Committee  8, Leninsky Prospect  Moskva 119049  Moscow  Russian Federation  Site: 20010 |
| City Clinical Hospital # 1 n.a. N.I. Pirogov  Ethics Committee  8, Leninsky Prospect  Moskva 119049  Moscow  Russian Federation  Site: 20010 | Clinical Hospital Of Emergency Care N.V. Soloviev  Ethics Committe  11 Ulitsa Zagorodnyj Sad  150003 Yaroslavl  Russian Federation  Site: 20015 |
| Clinical Hospital of Emergency Care N.V. Soloviev Ethics Committe  11 Ulitsa Zagorodnyj Sad  150003 Yaroslavl  Russian Federation  Site: 20015 | Rheumatology Research Institute Of Russian Academy Of  Medica  Ethics Committe  Kashirskoe Shosse, 34  115522 Moscow  Russian Federation  Sites: 20002 |
| Rheumatology Research Institute of Russian Academy of Medica  Ethics Committe  Kashirskoe Shosse, 34  115522 Moscow  Russian Federation  Sites: 20002, 20017 | Saratov Regional Clinical Hospital  rheumatology  1, Smirnovskoe uschelye  Saratovskaya oblast'  410053 Saratov  Russian Federation  Site: 20007 |
| Saratov Regional Clinical Hospital  Rheumatology  1, Smirnovskoe uschelye  Saratovskaya oblast  410053 Saratov  Russian Federation  Site: 20007 | SBHI of Yaroslavl Region "Clinical Hospital n.a. N.A.  Semashko"  12, Gagarina street  Yaroslavskaya oblast'  150023 Yaroslavl  Russian Federation  Site: 20008 |
| SBHI of Yaroslavl Region "Clinical Hospital n.a. N.A. Semashko  12, Gagarina street  Yaroslavskaya oblast  150023 Yaroslavl  Russian Federation  Site: 20008 | The Republican Hospital N.A. V.A. Baranov  Ethics Committe  3 Ulitsa Pirogova  185019 Petrozavodsk  Russian Federation  Site: 20013 |
| Ryazan State Medical University I.P. Pavlov  Ethics Committe  9, Vysokovoltnaya Ulitsa  390026 Ryazan  Russian Federation  Site: 20012 | Ulyanovsk Regional Clinical Hospital  Rheumatology  7, Tretyego Internatsionala ul.  432063 Ulyanovsk  Russian Federation  Site: 20014 |
| The Republican Hospital N.A. V.A. Baranov  Ethics Committe  3 Ulitsa Pirogova  185019 Petrozavodsk  Russian Federation  Site: 20013 | – |
| Ulyanovsk Regional Clinical Hospital  Rheumatology  7, Tretyego Internatsionala ul.  432063 Ulyanovsk  Russian Federation  Site: 20014 | – |
| Ryazan Regional Clinical Cardiological Dispensary Local Ethics Committee  96 Stroikova Ulitsa  390026 Ryazan  Russian Federation  Site: 20016 | – |
| The Llc Institute of Medical Trials  Lec  25 Koli Tomchaka Ul. Liter A3  196084 Saint-Petersburg  Russian Federation  Site: 20083 | – |
| **Spain** | |
| CEIC Corporació Sanitària Parc Taulí Fundació Parc Taulí  Edifici Santa Fe Ala izquierda, 2ª planta C/ Parc Taulí, 1 Barcelona 08208  Sabadell  Spain  Sites: 40045, 40049, 40099, 40101, 40102, 40103, 40104, 40105, 40106 | – |
| **United Kingdom** | |
| New Cross hospital  Rheumatology  New Cross hospital  Wolverhampton WV10 0QP  Wolverhampton  United Kingdom  Site: 40107 | Nuffield Orthopaedic Centre  ND07, NDORMS Windmill Road, Headington  Oxfordshire  OX3 7HE  Oxford  United Kingdom  Site: 40109 |
| Nuffield Orthopaedic Centre  ND07, NDORMS Windmill Road, Headington Oxfordshire  OX3 7HE  Oxford  United Kingdom  Site: 40109 | Bradford Royal Infirmary  Rheumatology  Duckworth Lane  BD9 6RJ  Bradford  United Kingdom  Site: 40111 |
| Royal Cornwall Hospital  Treliske Truro  Cornwall  TR1 3LJ  Cornwall  United Kingdom  Site: 40112 | Stamford and Rutland hospital  Ryhall Road  PE9 1UA  Stamford  United Kingdom  Site: 40116 |
| Barnsley Hospital NHS Foundation Trust  Gawber Road  S75 2EP  Barnsley  United Kingdom  Site: 40115 | – |
| **United States** | |
| Advarra  IRB/IEC  6100 Merriweather Drive, Suite 600  Maryland 21044  Columbia  United States  Sites: 50001, 50002, 50004, 50006, 50007, 50008, 50009, 50012, 50015, 50016, 50017, 50020, 50028, 50029, 50033, 50035, 50036, 50037, 50039, 50040, 50049, 50050, 50051, 50125 | Advarra  IRB/IEC  6100 Merriweather Drive, Suite 600  Maryland 21044  Columbia  United States  Sites: 50001, 50002, 50004, 50005, 50006, 50008, 50009,  50011 50012, 50015, 50016, ,50017, 50019, 50020,  50021, 50024, 50026, 50028, 50029, 50031, 50033,  50034, 50035, 50036, 50037, 50039, 50040, ,50047  50050, 50064, 50125 |
| WCG IRB  IRB/EC  1019 39th Avenue SE Suite 120  Washington 98374  Puyallup  United States  Site: 50010 | WCG IRB  IRB/EC  1019 39th Avenue SE Suite 120  Washington 98374  Puyallup  United States  Site: 50010 |
| – | Ochsner Institutional Review Board  Institutional Review Board  1514 Jefferson Highway  Louisiana 70121  New Orleans  UNITED STATES  Site: 50023 |
